# miR-221 and -222 target CACNA1C and KCNJ5 leading to altered cardiac ion channel expression and current density

**DOI:** 10.1007/s00018-019-03217-y

**Published:** 2019-07-16

**Authors:** Stephanie Binas, Maria Knyrim, Julia Hupfeld, Udo Kloeckner, Sindy Rabe, Sigrid Mildenberger, Katja Quarch, Nicole Strätz, Danny Misiak, Michael Gekle, Claudia Grossmann, Barbara Schreier

**Affiliations:** 1 Julius-Bernstein-Institute of Physiology, Martin Luther University Halle-Wittenberg, Magdeburger Str. 6, 06110 Halle/Saale, Germany; 2 Institute of Molecular Medicine, Martin-Luther-University Halle-Wittenberg, Heinrich-Damerow-Str. 1, 06120 Halle/Saale, Germany

**Keywords:** Electrical remodeling, Cardiomyocytes, Angiotensin II, Heart hypertrophy

## Abstract

MicroRNAs (miRs) contribute to different aspects of cardiovascular pathology, among others cardiac hypertrophy and atrial fibrillation. The aim of our study was to evaluate the impact of miR-221/222 on cardiac electrical remodeling. Cardiac miR expression was analyzed in a mouse model with altered electrocardiography parameters and severe heart hypertrophy. Next generation sequencing revealed 14 differentially expressed miRs in hypertrophic hearts, with miR-221 and -222 being the strongest regulated miR-cluster. This increase was restricted to cardiomyocytes and not observed in cardiac fibroblasts. Additionally, we evaluated the change of miR-221/222 in vivo in two models of pharmacologically induced heart hypertrophy (angiotensin II, isoprenaline), thereby demonstrating a stimulus-induced increase in miR-221/222 in vivo by angiotensin II but not by isoprenaline. Whole transcriptome analysis by RNA-seq and qRT-PCR validation revealed an enriched number of downregulated mRNAs coding for proteins located in the T-tubule, which are also predicted targets for miR-221/222. Among those, mRNAs were the L-type Ca^2+^ channel subunits as well as potassium channel subunits. We confirmed that both miRs target the 3′-untranslated regions of Cacna1c and Kcnj5. Furthermore, enhanced expression of these miRs reduced L-type Ca^2+^ channel and Kcnj5 channel abundance and function, which was analyzed by whole-cell patch clamp recordings or Western blot and flux measurements, respectively. miR-221 and -222 contribute to the regulation of L-type Ca^2+^ channels as well as Kcnj5 channels and, therefore, potentially contribute to disturbed cardiac excitation generation and propagation. Future studies will have to evaluate the pathophysiological and clinical relevance of aberrant miR-221/222 expression for electrical remodeling.

**Electronic supplementary material:**

The online version of this article (10.1007/s00018-019-03217-y) contains supplementary material, which is available to authorized users.

## Introduction

According to the World Health Organization, cardiovascular diseases are the leading causes of death worldwide, with functional and structural heart changes (“remodeling”) playing a major role. Cardiac remodeling is defined as a group of molecular, cellular, and interstitial changes that manifest as changes in size, shape, and function of the heart, resulting from cardiac injury or stress [1]. The pathophysiological processes result in hypertrophy or atrophy, fibrosis, and inflammation as well as changes in electrophysiology, affecting generation, duration, and propagation of action potentials. Ultimately, these changes cause heart dysfunction [1]. However, the underlying mechanisms are not sufficiently understood. Remodeling is often associated with a dysfunction of cardiomyocytes, alterations in their ion handling, metabolism and gene expression, including ion channel genes.

Cardiomyocytes, although quantitatively being not the major cardiac cell type [2], they are functionally the most relevant cell type. To allow an appropriate temporal and spatial propagation of the action potential and thereby contraction, the interaction of several ion channels and transporters is needed. Among the ion channels influencing action potential generation, duration and propagation are the L-type Ca^2+^ channel (Cav1.2), the voltage-gated potassium channel Kv4.2 or the G-protein-activated inwardly rectifying potassium channel (GIRK1/4). Changes in expression pattern, channel density or conductance contribute to electrical remodeling.

MicroRNAs (miRs) are among the differentially expressed DNA transcripts during cardiac remodeling [3, 4]. miRs are short non-coding, conserved RNAs with a size of 20–22 nucleotides regulating gene expression by post-transcriptional processes [5]. They bind mainly to the 3′-untranslated region (3′-UTR) of target mRNAs, and thereby repress either translation or induce degradation of mRNA [6, 7]. miRs with the same seed sequence belong to the same miR family, although the targets of the family members might differ [7]. From the known miRs, at least 200 are expressed in the cardiovascular system [8]. As it has been proposed that each miR family has at least 300 targets [7], these small non-coding RNAs might have a major impact on the remodeling processes occurring during heart hypertrophy. The necessity of miR expression in the heart for proper heart development and function has been demonstrated by cardiac-specific deletion of the miR-processing enzyme Dicer. Animals that lack cardiac Dicer during embryogenesis die shortly after birth [9, 10]. Additionally, inducible deletion of Dicer in the adult heart leads to biventricular enlargement and myocyte hypertrophy [11].

The contribution of miRs to structural and electrical remodeling has been studied in animal models, revealing that among others, miR-1, -21, -26, -133, -208, and -499 are associated with cardiac remodeling [12, 13]. Additionally, some miRNAs are described to regulate ion channel subunits involved in arrhythmias, like miR-1, miR-26 or miR-328 [14–16]. Yet, in contrast to structural remodeling, miR-mediated alteration of cardiac electrophysiology has not been evaluated to a comparable breadth.

We analyzed miR expression in a genetic mouse model with severe cardiac hypertrophy [17] that is accompanied by electrophysiological changes [18]. The electrophysiological changes in this model are not due to increased fibrosis. Among the upregulated miRNAs, the miR 221/222 cluster was the most prominent. As an impact of this cluster on heart function has been suggested before [19], we decided to focus on these two miRNAs. Upregulation of miR-221 and -222 in the diseased hearts and cardiomyocytes was accompanied by the downregulation of predicted mRNA targets coding for proteins located in T-tubules. Further analysis revealed that miR-221 and -222 bind to the 3′-UTRs of the Cacna1c subunit of the L-type Ca^2+^ channel and of the Kcnj5 subunit of the GIRK1/4 channel. Finally, miRs-221/222 reduce the Cav1.2 expression and I_Ca,L_ current density as well as the GIRK4 protein content and ion flux through GIRK1/4 in HL-1 cells.

## Materials and methods

### Animal procedures

All mouse experiments described in this manuscript were approved by the local government (Landesverwaltungsamt Sachsen-Anhalt, Germany, permit number: 42502-2-1124 and -1201 MLU) and were performed according to the guidelines of the directive 2010/63/EU. Mice were kept in the facilities of the University of Halle-Wittenberg at a room temperature of 20 ± 1 °C and with a 12 h/12 h light/dark cycle. All animals were 6 month of age when included in the experiments. Generation, genotyping, and the cardiovascular phenotype of EGFR KO animals were described before [17]. Mice with a deletion of the EGFR in vascular smooth muscle cells and a strong reduction in cardiomyocytes are termed either EGFR^Δ/ΔVSMC&CM^ or knockout (KO). Electrocardiography recordings were obtained from isoflurane-anesthetized animals as described before [18].

For angiotensin II (AII, 1000 ng/kg BW/min over 3 weeks) or isoprenaline (iso, 30 mg/kg/day for 2 weeks) treatment, male animals were anesthetized with isoflurane (~ 2% v/v in 100% O_2_, 1 l/min) and Alzet minipumps (1004) were implanted subcutaneously in the back of the animals. 5–10 animals per group were included into the study. Carprofen (5–10 mg/kg BW, Rimadyl, Pfizer, New York, USA) was injected subcutaneously immediately before pump implantation. If necessary, pain relief was repeated every 8 h. Mice were sacrificed by cervical dislocation in isoflurane anesthesia. Hearts were removed and the weight was normalized to tibia length (HW/TL). Subsequently, the heart was divided for biochemical and histological analysis. Cardiomyocytes and cardiac fibroblasts were isolated as described before [20] from whole hearts. Fibroblasts were isolated by incubation of the supernatant from the cardiomyocyte isolation overnight in Petri dishes. The degree of interstitial fibrosis in hearts as well as cross-sectional diameter of cardiomyocytes was determined by evaluation of Sirius red or hematoxylin/eosin-stained slices as described before [21] from ventricular slices.

### Gene expression analysis

For all analyses, total RNA was isolated either from whole hearts or isolated cells using the InviTrap spin tissue RNA mini kit (STRATEC, Berlin, Germany) or the TRIzol Reagent (Invitrogen, Darmstadt, Germany). 1 µg of total RNA was treated with DNase I (RNase-free) (NEB, Frankfurt, Germany) and reverse transcription (RT) was performed with random primers using SuperScript II reverse transcriptase (Invitrogen, Darmstadt, Germany), according to the manufacturer’s instructions.

Gene expression was analyzed via real-time RT-PCR and mRNA amount was normalized to 18S rRNA or Gapdh. Sequence of primers, as well as annealing temperature and RefSeq accession number/id are given in Supplementary Table S1.

To determine the absolute copy number of RNA, droplet digital PCR (ddPCR) was performed using the QX200 system of BioRad (Munich, Germany). cDNA was prepared as described above and used in ddPCR at the same conditions as in real-time RT-PCR.

For TaqMan™ ddPCR, a primer pair and a FAM-labeled probe specific for either miR-221 or miR-222 were used simultaneously with a primer pair and a HEX-labeled probe specific for U6 (Applied Biosystems, Karlsruhe, Germany). The list of TaqMan™ assays purchased from Applied Biosystems is given in Supplementary Table S2.

### Next generation sequencing of mRNA and cluster analysis

Sequencing was performed with an Illumina HiScanSQ at the Core Unit DNA Technologies of the Medical Faculty, University Leipzig, Germany. Libraries were prepared with indexed adapters, and clusters were generated on the cluster flow cells. cDNA fragments were hybridized to the lawn of complementary primers followed by “bridge amplification”. Paired-end sequencing was performed by synthesis (SBS) via reversible terminator-based method. Deep sequencing data of 101 bp reads from each lane were de-multiplexed and data of each sample were analyzed using FastQC, cutadapt, TopHat2, samtools, featureCounts, TMM, FPM. Differential expression was tested by Poisson exact test [22]. Significant differential expression was determined by a significance level of 0.05 (FDR ≤ 0.05). mRNA enrichment analysis was performed by g:Profiler [23] and GOrilla [24].

### Next generation sequencing of microRNA

Sequencing was performed as described previously [25]. 500 ng of RNA from each sample was used with the TruSeq™ Small RNA sample prepkit v2 (Illumina). The barcoded libraries were size restricted between 140 and 165 base pairs (bp) for additional enrichment of miRs, purified and quantified using the Library Quantification Kit-Illumina/Universal (KAPA Biosystems, Woburn, USA). Sequencing of 50 bp was performed with an Illumina HighScan-SQ sequencer using version 3 chemistry and flow cell. All procedures were performed according to the instructions of the respective manufacturer. The R packages DESeq2 and EdgeR were used for normalization and to calculate differential expression of miRs.

### HL-1 cell line

HL-1 cells were maintained in Claycomb medium (Sigma, Munich, and Germany) with the following supplements: 10% FCS (Biochrom, Berlin, Germany), 2 mM l-glutamine (Sigma), 100 µM noradrenaline (Sigma), 100 µ/ml penicillin, and 100 µg/ml streptomycin (Sigma).

HL-1 cells were transfected with 30 nM of miRCURY LNA miR-221 or miR-222 mimics or mimic negative control (Exiqon, Vedbaek, Denmark) using 5 µl Lipofectamine (Thermo Fisher Scientific, Waltham, USA) in 1.5 ml DMEM (Biochrom, Berlin, Germany; without FCS) following manufacturer’s instructions. After 24 h, the medium was changed and cells were kept on Claycomb medium with supplements for further 48 h.

For Western blot analysis, HL-1 cells were washed with PBS and lysed in RIPA buffer and sonicated (UP100H; Hielscher, Teltow, Germany). Cell lysates were matched for protein content. After separation, the proteins were transferred to a PVDF membrane (Thermo Fisher Scientific, Waltham, USA) for p27 or a nitrocellulose membrane (GE Healthcare, Buckinghamshire, UK) for GIRK1, GIRK4, and HSP90 detection. The membrane was incubated with primary antibodies (p27: 1:500, ab137736, Abcam, Cambridge, UK; GIRK1: 1:1000, ab129182, Abcam, Cambridge, UK; GIRK4: 1:750, ab113699, Abcam, Cambridge, UK; HSP90: 1:1000, 4874, Cell Signaling Technology, Danvers, USA) at 4 °C overnight. The bound primary antibody was visualized using horseradish peroxidase-conjugated secondary IgG (anti-rabbit, 1:10,000 for p27, 1:20,000 for GIRK1/4 and HSP90, Rockland, Limerick, USA) and the ECL™ system (Amersham, Freiburg, Germany). Densitometry analysis was performed with Quantity One software (BioRad, Munich, Germany).

### Electrophysiology

Single HL-1 cells were plated for 24 h on gelatin/fibronectin-coated 35-mm Petri dishes in 2 ml of Claycomb medium. The cells were transfected with miR-221/222 mimics as described above. 48 h after transfection, current recordings were performed in the whole-cell configuration of the patchclamp technique using an Axopatch 200A patch-clamp amplifier (Axon Instruments, Inc., Burlingame, CA, USA). Patch pipettes were fabricated from thick wall (2-mm OD) borosilicate glass capillaries (Hilgenberg, Malsfeld, Germany) and filled with an internal solution of the following composition (in mmol/L): 130 CsCl, 20 TEACl, 10 EGTA, 5 Na_2_ATP, 6 MgCl_2_, 10 HEPES (pH was adjusted with CsOH to 7.2). Electrical resistances of the fire-polished electrodes were 3–4 MΩ when filled with internal solution. L-type Ca^2+^ currents were recorded in a Na^+^-free and K^+^-free bath solution containing (in mmol/L): 150 Tris–Cl, 10 CaCl_2_, 10 glucose, 10 HEPES (pH was adjusted with Tris–OH to 7.4). Current signals were sampled at 16–40 kHz and low pass filtered at 5 kHz with a four-pole Bessel filter and stored for off-line analysis (ISO2, MFK, Germany). Series resistance was partially compensated (> 70%). By integrating the capacitive current at the end of 10 ms long voltage step (− 80 to − 70 mV), the input capacitance of the cells was obtained. The peak amplitude of the inward current was normalized to the input capacitance to obtain the current density (pA/pF) to compensate for differences in cell size. All experiments were carried out at room temperature (20–24 °C). HL-1 cells express both T-type and L-type Ca^2+^ currents [26–28]. Since in this study we sought to investigate the effect of miR-221/222 solely on the activity of L-type Ca^2+^ channels, we used a voltage clamp protocol to separate the two currents from each other. T-type Ca^2+^ channels—recorded with 10 mmol/L Ca^2+^ as the charge carrier—were inactivated using a holding potential of − 35 mV without affecting the availability of L-type Ca^2+^ channels. Initially, we performed current density measurements by depolarizing the cells from a holding potential of − 40 or − 35 mV to various test potentials in 10 mV increments (from − 40 to 65 mV). As we observed a reduction in peak density but not a shift in current density–voltage relationship (Supplementary Figure S1), in further experiments only peak inward current was determined. To obtain the maximal peak inward current (peak I_Ca,L_), HL-1 cells were depolarized every 8 s for 100 ms from a holding potential of − 35 mV to various test potentials (15–30 mV in 5 mV increments).

### Dual luciferase reporter assay

Reporter constructs (pEZX-MT06 dual luciferase reporter) contained the 3′-UTRs of murine ion channel mRNAs listed in Supplementary Table S3 downstream of the firefly luciferase. 3′-UTRs longer than 3.5 kb were divided into fragments. The vectors were transfected into HEK293 cells, 10 ng each. Additionally, the cells were transfected either with 30 nM of miRCURY LNA miR-221 mimics, miR-222 mimics or mimic negative control using 1.5 µl Polyfect (Qiagen, Germantown, USA). After 24 h, the supernatant was removed, and after further 48 h, cells were lysed and the luciferase activity in the lysate was measured using the Dual Luciferase Assay System (Promega, Madison, USA). The firefly luciferase activity was normalized to the renilla luciferase activity. After that, values were normalized to mimic negative control as well as empty vector.

### FluxOR assay

HL-1 cells were transiently incubated with miR-221/222 mimics for 24 h as described above. 48 h after start of transfection, cells were seeded onto a 96-well plate and incubated for another 24 h. FluxOR™ II Green Potassium Ion Channel Assay (Invitrogen™) was performed according to the manufacturer’s instructions using the Operetta CLS High-Content Analysis System (Perkin Elmer, Krakow, Poland) with a thallium ion (Tl^+^) concentration of 1 mM. To confirm that carbachol-induced (CCH, 10 µM final concentration, Sigma-Aldrich, Munich, Germany) thallium ion flux was indeed carried out by GIRK4, we performed initial experiments with tertiapin q (TQ, 100 nM final concentration, Alomone Labs, Jerusalem, Israel), an inhibitor of GIRK1/4. After obtaining the baseline fluorescence (F0), a buffer containing Tl^+^ alone (control) or additionally carbachol with or without tertiapin q was added and the fluorescence was measured every 15 s (30 time points after stimulation, Supplementary Figure S2).

Fluorescence data were normalized to baseline fluorescence (F/F0). The time course of F/F0 was integrated to obtain the area under the curve (AUC). Carbachol effect (CE) was calculated as AUC (carbachol)-AUC (control) for each individual experiment and afterward normalized to the mean CE of the corresponding mimic control (scramble).

### Statistical analysis

Data are presented as mean ± standard error of mean (SEM). ANOVA followed by post hoc testing, Student’s *t* test or Mann–Whitney rank sum test were used as applicable according to pre-test data analysis by Sigma Plot 12.5. A *p* value < 0.05 was considered significant. Biometrical planning was performed with *α* = 0.05 and *β* = 0.8, resulting in sample sizes between five and 15 samples/group depending on the experimental setting. For next generation sequencing (NGS) either for miRNAs or mRNAs, DEseq analysis was performed. Graphics were prepared using Sigma Plot 12.5.

## Results

### Differential miR expression

To investigate the impact of miRNA on electrical remodeling, we analyzed differential miR expression (NGS; wild type and knockout animals from the EGFR^Δ/ΔVSMC&CM^ mouse line *N* = 6 per group) in hearts of a genetic model with extensive hypertrophy (HW/TL: WT 7.4 ± 0.3 mg/mm and KO 20.5 ± 1.6 mg/mm, *N* = 19–23 animals/group) without major signs of fibrosis or heart failure [17]. Previous ECG analysis of this mouse strain revealed that EGFR^Δ/ΔVSMC&CM^ mice showed prolonged *p*-duration (14 ± 1 ms in wild type versus 17 ± 1 ms in knockouts; *p* < 0.05; *N* = 8), QRS intervals (15 ± 1 ms in wild type versus 22 ± 2 ms in knockouts; *p* < 0.05; *N* = 8), as well as QTc intervals (61 ± 3 ms in wild type versus 82 ± 6 ms in knockouts; *p* < 0.05; *N* = 5) indicating disturbed excitation propagation [18]. Parameters for heart rate variability did not differ between the genotypes (SDNN: WT 10.9 ± 2.5 ms and KO 9.2 ± 1.5 ms; SDδNN: WT: 13.1 ± 4.2 ms and KO 9.0 ± 1.5 ms; RMSSD: WT: 13.1 ± 4.2 ms and KO 9.0 ± 1.5 ms, *N* = 6–8 animals/group).

**Fig. 1:**
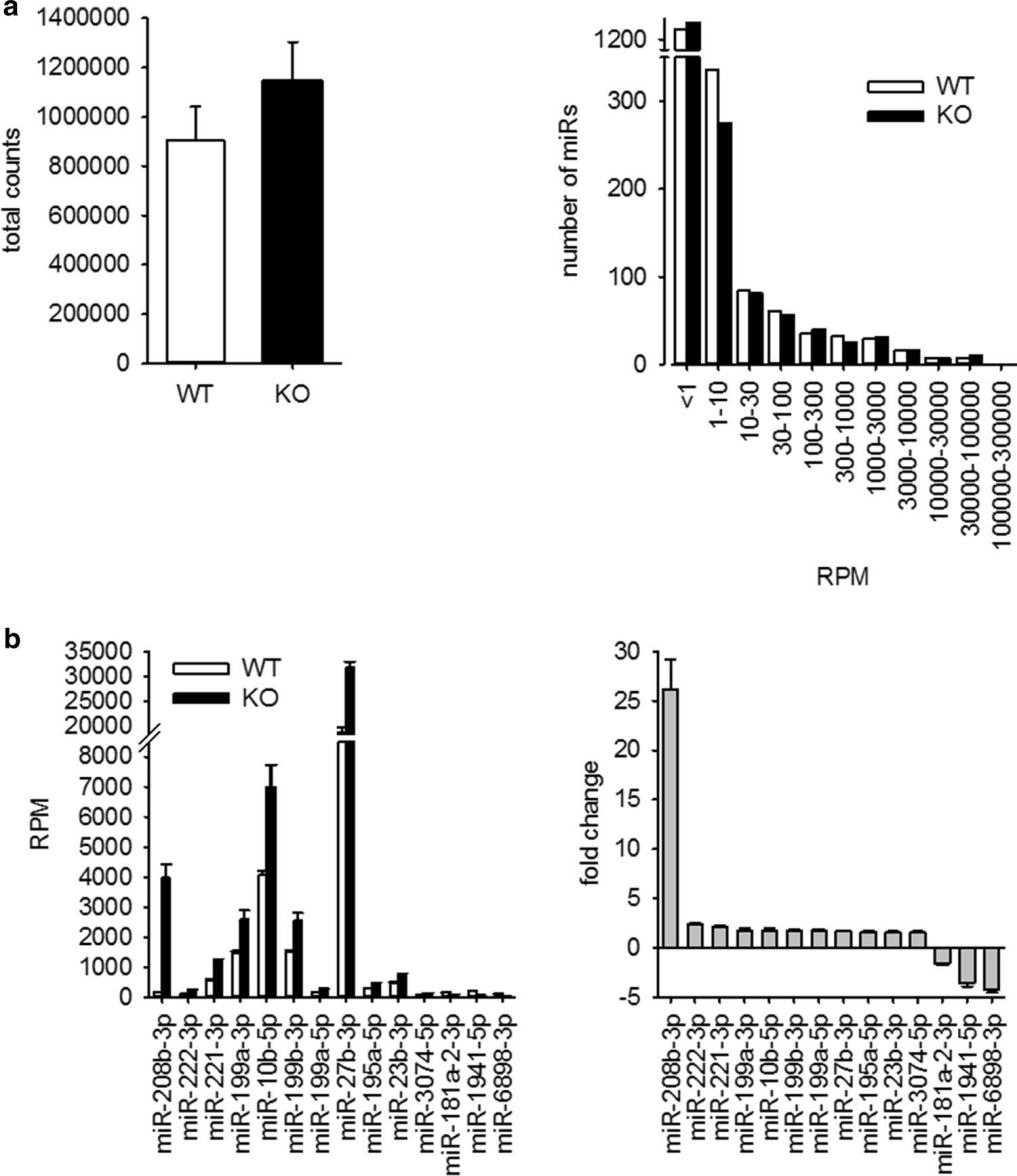
miRNA expression in hearts of mice with severe heart hypertrophy. **a** miRNA expression was analyzed by next generation sequencing in hearts of WT and KO mice. **b** In the hearts of KO animals, 14 miRNAs were differentially expressed compared to WT animals (thresholds: ≥ 100 RPM in WT for downregulated miR; ≥ 100 RPM in KO for upregulated miR; fold change ≥ |1.5|; *p* < 0.01, for detailed information see Supplementary File S1, *N* = 6 animals/group)

For NGS analysis of miRNAs, whole-heart samples where used. In a first step, we compared the reads per million between WT and KO animals. From 1908 miRs annotated in the mouse genome, only a minority showed an abundance of more than one read per million in WT (610) and KO (543) animals (Supplementary File S1). These miRNAs were allotted according to their reads per million to different groups. The distribution of miRNAs in these groups was evaluated. Because neither absolute counts nor gross distribution of miRs in the different groups was different between WT and KO animals (Fig. 1a), we excluded substantial alteration in overall miR generation and processing in the hypertrophied hearts. Supplementary File S1 shows the data for all miRs. For the analysis of differential expression of single miRNAs, we used the following thresholds: only miRNAs with a RPM ≥ 100 in WT for downregulated or ≥ 100 RPM in KO for upregulated miR were taken into account. miRNAs were considered to be significantly changed when the fold change was ≥ |1.5| and the *p* value between WT and KO samples was < 0.01. According to these parameters, 14 miRs were differentially expressed between WT or KO animals (Fig. 1b).

### Validation of differential microRNA expression

**Fig. 2:**
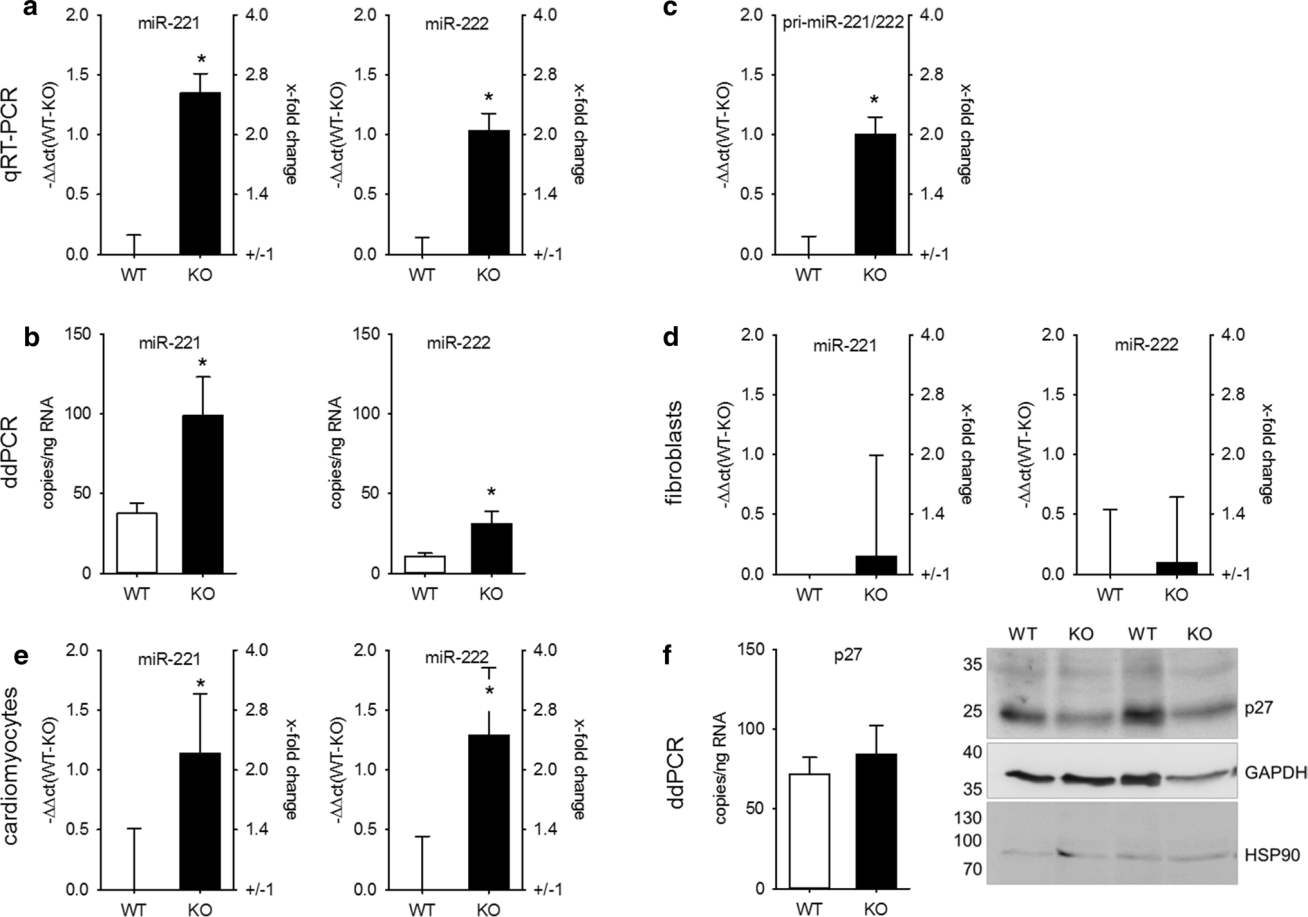
Altered miR-221/222 expression in hearts of mice with severe heart hypertrophy is due to enhanced miRNA expression in cardiomyocytes. miR-221 (right panels) and miR-222 (left panels) expression were evaluated by TaqMan qRT-PCR (**a**, relative change compared to WT) and droplet digital PCR (**b**) in hearts of wild type and knockout animals in an additional cohort. *N* = 30 animals/group. The increase in miR-221 and -222 could be observed in isolated cardiomyocytes (**c**, *N* = 18 animals/group relative change compared to WT). Pri-miR-221/222 was increased in mice with heart hypertrophy (**d**, whole heart samples *N* = 30 animals/group, relative change compared to WT). No significant difference in the amount of miR-221 and -222 could be detected in cardiac fibroblasts from wild type or knockout animals (**e**, *N* = 10 animals/group, relative change compared to WT). To evaluate if the increase in miR-221 and -222 expression correlates with the expression of a validated cardiac target for these miRNAs, we analyzed p27 mRNA amount (*N* = 30 animals/group, relative change compared to WT) and protein content in hearts from WT and KO animals (**f**, *N* = 19–20 animals/group). Panel f shows a representative Western blot image

As the impact of mmu-miR-208b-3p on structural and electrical heart remodeling [29] and as a marker for an increased risk for death after myocardial infarction [30] has already been reported, we focused on miR-221/222, the second and third most upregulated miR. The changes in miR content were validated via TaqMan qRT-PCR (Fig. 2a) and ddPCR (Fig. 2b) from a separate cohort of whole-heart samples from WT and KO mice and confirmed the data from NGS. As the fibroblasts outnumber the cardiomyocytes in the heart [2], freshly isolated cardiomyocytes from adult WT and KO mice were analyzed. Cardiomyocytes from hypertrophied hearts also showed an increased amount of miR-221 and miR-222 (Fig. 2c), corresponding to the results from whole hearts of adult mice. Additionally, we observed an increased expression of pri-miR-221/222 (Fig. 2d). Cardiac fibroblasts showed no change in miR-221/222 expression (Fig. 2e).

We also determined miR-221/222 expression in the hearts of newborn knockout animals (1 week of age) that displayed a 27% increase in heart weight compared to wild-type animals (6.2 ± 0.4 versus 8.0 ± 0.4 mg/g body weight; *N* = 8 animals/group), but observed no significant difference (− ΔΔct = 0.34 ± 0.16; *N* = 8 animals/group).

**Fig. 3:**
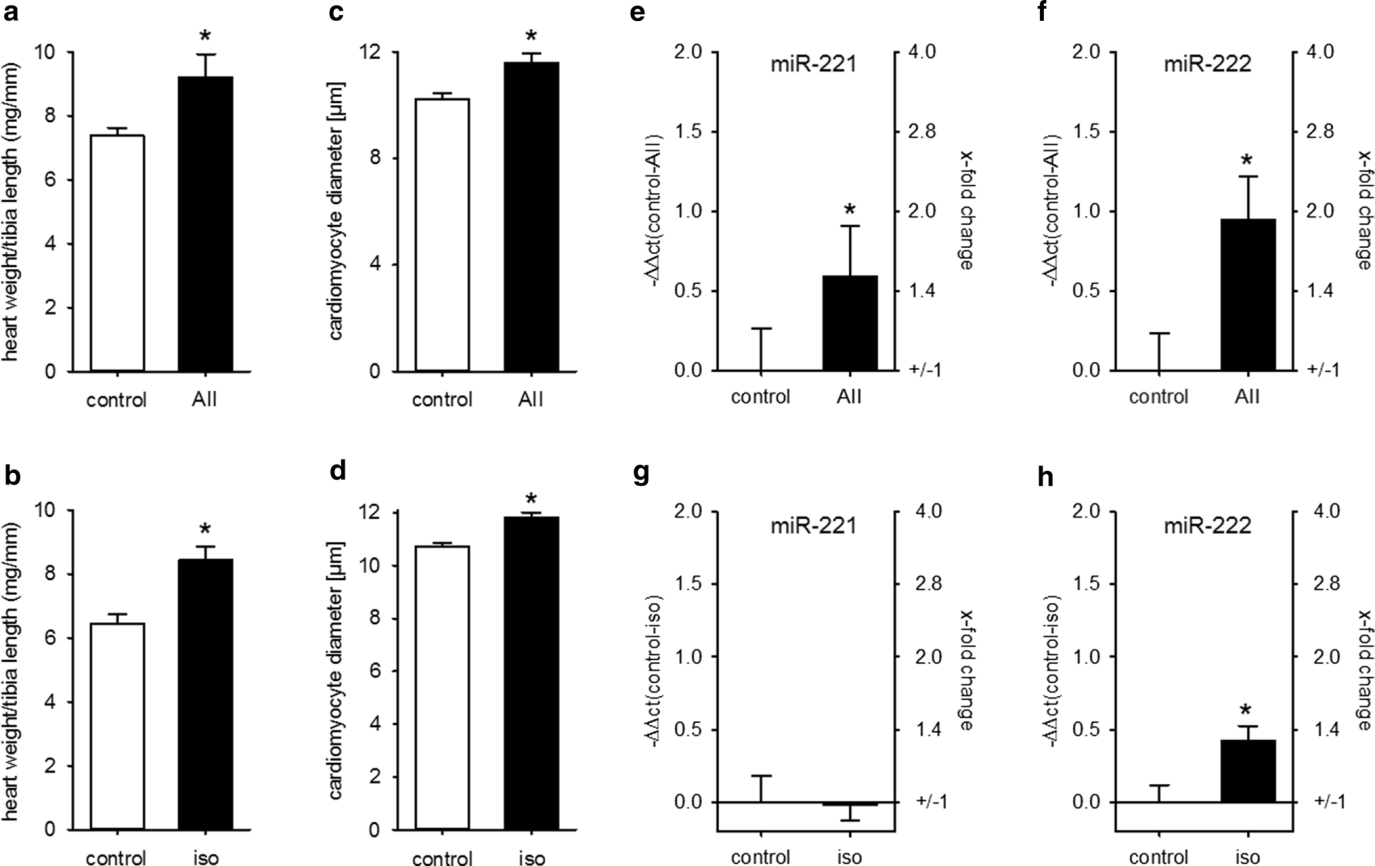
Heart hypertrophy alone is not sufficient to increase cardiac miR-221/222 expression. Infusion of AII or iso induced a comparable increase in HW/TL (**a**, **b**) and cardiomyocyte diameter (**c**, **d**) compared to control animals. While AII treatment increased miR-221 (**e**) and miR-222 (**f**) amount in the hearts of the animals, iso induced no biological relevant change in those miRNAs (**g**, **h**) (AII: *N* = 12–15 animals/group, iso: 5–6 animals/group, relative change compared to control)

We investigated miR-221/222 expression in two other models of pathological heart hypertrophy: AII infusion as a model for pressure overload-induced heart hypertrophy [31] and isoprenaline infusion as a model for ischemic heart failure [32]. Neither AII nor isoprenaline caused heart failure, as lung weight/tibia length was not altered by substance infusion. In contrast, while AII had no impact on interstitial fibrosis, the percentage of Sirius red-stained tissue was increased in isoprenaline-treated animals (Supplementary Figure S3). Infusion of AII as well as isoprenaline caused a significant and comparable increase in HW/TL and an increase in cardiomyocyte diameter (Fig. 3a–d). In contrast, while the expression of both miRs was elevated in the AII-treated animals, there was no biologically relevant increase in isoprenaline-treated mice (Fig. 3e–h).

### Assessment of reduced miR-221 target expression (p27)

Additionally, we analyzed if the increase in miR-221/222 resulted in the reduction of a validated target, p27 (cyclin-dependent kinase inhibitor 1B) [6]. There was no alteration of p27 mRNA in whole-heart lysates between WT and KO animals as determined by ddPCR, but a reduction in protein content (protein: WT: 100.00 ± 6.9%, KO: 78.52 ± 6.8% of WT, *p* < 0.05, *N* = 19–20 animals/group, Fig. 2f). Furthermore, comparison of miR-221/222 and p27 mRNA copy number (Fig. 2b, f) shows that the amount of both is in the same range; therefore, an impact of these two miRNAs on protein content without additional supporting factors seems to be reasonable. In summary, we conclude that miR-221 and miR-222 are upregulated in hypertrophied hearts of mice and that this upregulation leads (1) to a functionally relevant increase in the copy number of the miRs and (2) a reduction of a relevant, confirmed cardiac target of these miRs indicating a post-transcriptional regulation.

### Transcriptome determination and enrichment analysis

**Fig. 4:**
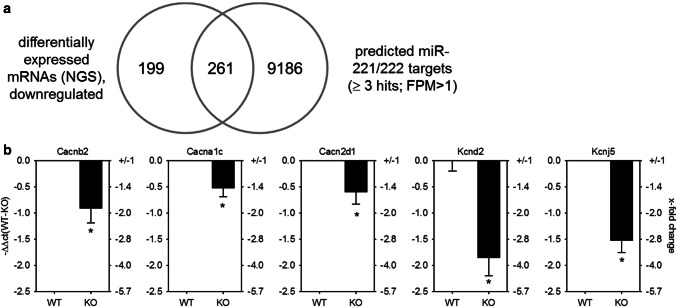
GO-term cluster analysis revealed an enrichment of mRNAs being downregulated and a target for miR-221/222 involved in cation channel complexes and T-tubule function. **a** Comparison of genes determined by whole transcriptome sequencing, being downregulated in mice with heart hypertrophy to target genes for miR-221/222 revealed an enrichment of genes involved in cardiac action potential (*p* = 5.30*E* − 8, g:Profiler, *N* = 6/group), with a threshold of *p* ≤ 0.01, FDR < 0.05. **b** Real-time qRT-PCR with heart samples from an additional cohort of mice w/o heart hypertrophy confirmed these findings (*N* = 30 animals/group, relative change compared to WT)

To identify potential targets that may be downregulated by miR-221/222 and contribute to electrical remodeling before heart failure in the hypertrophied heart, transcriptome analysis by RNA-seq of hearts from EGFR^Δ/ΔVSMC&CM^ and their wild-type littermates was performed and compared with predicted miR-221/222 targets (Fig. 4a). Of 22,026 annotated genes, 460 protein coding genes were detectable and downregulated (Supplementary File S2) in KO animals. We compared these genes with the target genes for miR-221/222 predicted by miRWalk 2.0. 261 genes were identified as miR-221/222 targets by ≥ 3 data bases (Supplementary File S2). Cluster analysis of those genes with g:Profiler and GOrilla revealed an enrichment of genes coding for proteins localized either to the T-tubule and/or involved in cation channel complex (Supplementary File S3). In Table 1, a list of genes included in the two clusters is given. For further analysis, we chose only genes with an FPM > 10 in wild-type animals, namely the three subunits of the L-type Ca^2+^ channel (Cacna1c, Cacnb2, Cacna2d1) as well as the potassium channel subunits, Kcnd2 and Kcnj5. The expression of these subunits was validated by qRT-PCR in whole-heart lysates from EGFR^Δ/ΔVSMC&CM^ mice. The downregulation of all three L-type Ca^2+^ channel subunits as well as the two potassium channel subunits on mRNA level could be confirmed in an additional animal cohort (Fig. 4b). To test the hypothesis that a ~ twofold miR-221/222 upregulation might impact mRNA amounts of the three ion channels, the copy numbers of Cacna1c, Kcnd2, and Kcnj5 mRNAs were analyzed by ddPCR. Analysis revealed a copy number of 399 ± 66 copies/ng RNA for Cacna1c, 43 ± 7 copies/ng RNA for Kcnd2, and 89 ± 13 copies/ng RNA for Kcnj5 (*N* = 9–10 animals/analysis). These copy numbers are in a similar range as miR-221/222 copy numbers (Fig. 2b).

**Table 1:** Differentially expressed genes being targets of miR-221/222 and belonging to the GO cluster “cation channel complex” and “T-tubule”

Gene name	RefSeq	Protein	Cation channel complex	T-tubule	WT mean	WT SD	KO mean	KO SD	LogFC_WT-KO	FDR_WT-KO
**Abcc9**	NM_021042	ATP-binding cassette, sub-family C (CFTR/MRP), member 9	x		324.8	22.0	205.2	24.8	− 0.66	9.06*E* − 07
**Adra1a**	NM_001271760	Adrenergic receptor, *α*1a		x	25.5	2.2	12.2	2.3	− 1.03	3.61*E* − 09
**Cacna1c**	NM_001256002	Calcium channel, voltage-dependent, L-type, *α*C subunit	x	x	297.3	24.4	215.0	20.8	− 0.46	1.39*E* − 03
**Cacna1** **h**	NM_001163691	Calcium channel, voltage-dependent, T-type, *α*1H subunit	x		7.2	1.6	2.6	0.8	− 1.50	5.68*E* − 07
**Cacna1** **s**	NM_014193	Calcium channel, voltage-dependent, L-type, *α*1S subunit	x	x	6.9	1.0	3.9	1.0	− 0.78	8.74*E* − 03
**Cacna2d1**	NM_001110844	Calcium channel, voltage-dependent, *α*2/δ subunit 1	x	x	55.9	3.3	40.6	5.6	− 0.46	1.10*E* − 02
**Cacnb2**	NM_001309519	Calcium channel, voltage-dependent, *β*2 subunit	x	x	64.9	9.0	40.3	5.2	− 0.69	7.26*E* − 05
**Cntn2**	NM_177129	Contactin-2	x		3.5	1.1	1.1	0.3	− 1.49	1.13*E* − 03
**Herpud1**	NM_022331	Homocysteine-inducible, endoplasmic reticulum stress-inducible, ubiquitin-like domain member 1	x		49.3	10.7	33.3	3.2	− 0.56	2.20*E* − 02
**Kcna1**	NM_010595	Potassium voltage-gated channel, shaker-related subfamily, member 1	x		2.2	0.8	0.7	0.3	− 1.63	1.20*E* − 02
**Kcnc3**	NM_001290682	Potassium voltage-gated channel, Shaw-related subfamily, member 3	x		2.8	0.7	1.2	0.6	− 1.02	2.66*E* − 02
**Kcnd2**	NM_019697	Potassium voltage-gated channel, Shal-related family, member 2	x	x	15.0	1.9	7.5	1.1	− 1.02	5.25*E* − 07
**Kcnip2**	NM_001276358	Kv channel-interacting protein 2	x		53.7	2.9	34.8	5.2	− 0.62	9.74*E* − 05
**Kcnj2**	NM_008425	Potassium inwardly-rectifying channel, subfamily J, member 2	x	x	25.0	5.8	15.8	1.9	− 0.64	1.86*E* − 02
**Kcnj3**	NM_008426	Potassium inwardly-rectifying channel, subfamily J, member 3	x	x	15.4	2.0	7.0	3.8	− 1.23	1.28*E* − 03
**Kcnj5**	NM_010605	Potassium inwardly-rectifying channel, subfamily J, member 5	x	x	23.8	4.4	12.3	3.0	− 0.96	1.30*E* − 04
**Scn4a**	NM_133199	Sodium channel, voltage-gated, type IV, *α*	x		24.3	2.6	12.9	2.6	− 0.95	1.24*E* − 06
**Shisa6**	NM_001034874	Shisa family member 6	x		2.1	1.4	0.3	0.3	− 2.35	1.61*E* − 03
**Ryr2**	NM_023868	Ryanodine receptor 2, cardiac	x		1685.2	156.6	1110.4	44.7	− 0.60	5.21*E* − 08

Among those, genes were the three subunits for the cardiac L-type Ca^2+^ channel (Cacnb2, Cacna1c, Cacna2d1), a voltage-gated potassium channel (Kcnd2), and both subunits of the acetylcholine-activated, inwardly-rectifying potassium channel (GIRK4, Kcnj5 and GIRK1, Kcnj3)

*N* = 6 animals/group, *WT* wild type, *KO* knockout, *SD* standard deviation, log*FC* logarithmic fold-change, *FDR* false discovery rate, amounts for WT and KO are given as fragments per million

### miR-221/222 leads to a downregulation of L-type Ca^2+^ channel and GIRK4

**Fig. 5:**
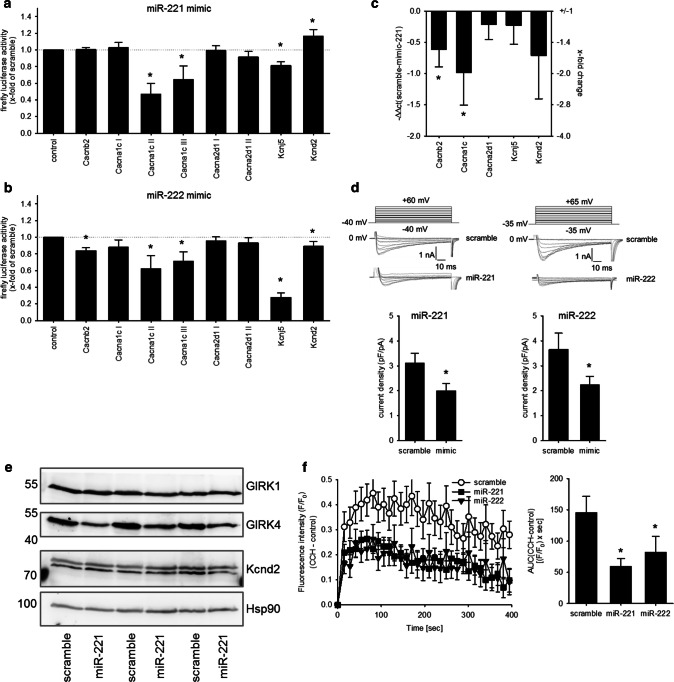
miR-221/222 reduce L-type Ca^2+^ channel current and Kcnj5 (GIRK4) protein amount by targeting Cacna1c or Kcnj5 3′-UTR. **a** To evaluate if the miRNAs bind to the 3′-UTR dual luciferase constructs containing the 3′-UTR from the L-type Ca^2+^ channel subunits, the potassium channel subunits, the seed sequence or an empty vector was transfected in HEK293 cells either with or without scramble or miR-221 mimic. miR-221 mimic reduced the luciferase activity of the Cacna1c-II, Cacna1c-III, and the Kcnj5 construct. **b** While miR-222 mimic reduced the luciferase activity for Cacnb2, Cacna1c-I, Cacna1c-III, Kcnj5, and Kcnd2 significantly (*N* = 4–9 experiments/group). **c** HL-1 cells were transfected either with scrambled or mimics for miR-221. After 48 h, the mRNA for Cacnb2 and Cacna1c was reduced (*N* = 5–6 wells/group, relative change compared to scramble). **d** To confirm the effect on L-type Ca^2+^ channel, we performed patch clamp analysis in HL-1 cells transfected with scramble, miR-221 or miR-222 mimics. miR-221 and -222 mimic reduced the I_Ca,L_ current (*n* = 19–46 cells/group, *N* = 3–5 experiments). Representative current tracings for control and mimic are given. **e** Western blot analysis for GIRK1, GIRK4, and Kcnd2 was performed in HL-1 cells treated either with scrambled (control) or miR-221 mimics (*N* = 3 per group). f HL-1 cells were transfected either with scramble, miR-221 or miR-222 mimics and GIRK4-dependent ion flux was measured by fluorescence changes of a thallium-sensitive dye. In HL-1 cells transfected with miR-221/222 mimics, the area under the curve and thereby the ion flux over time were significantly reduced compared to control cells. (*N* = 4 experiments, *n* = 3 wells)

**Fig. 6:**
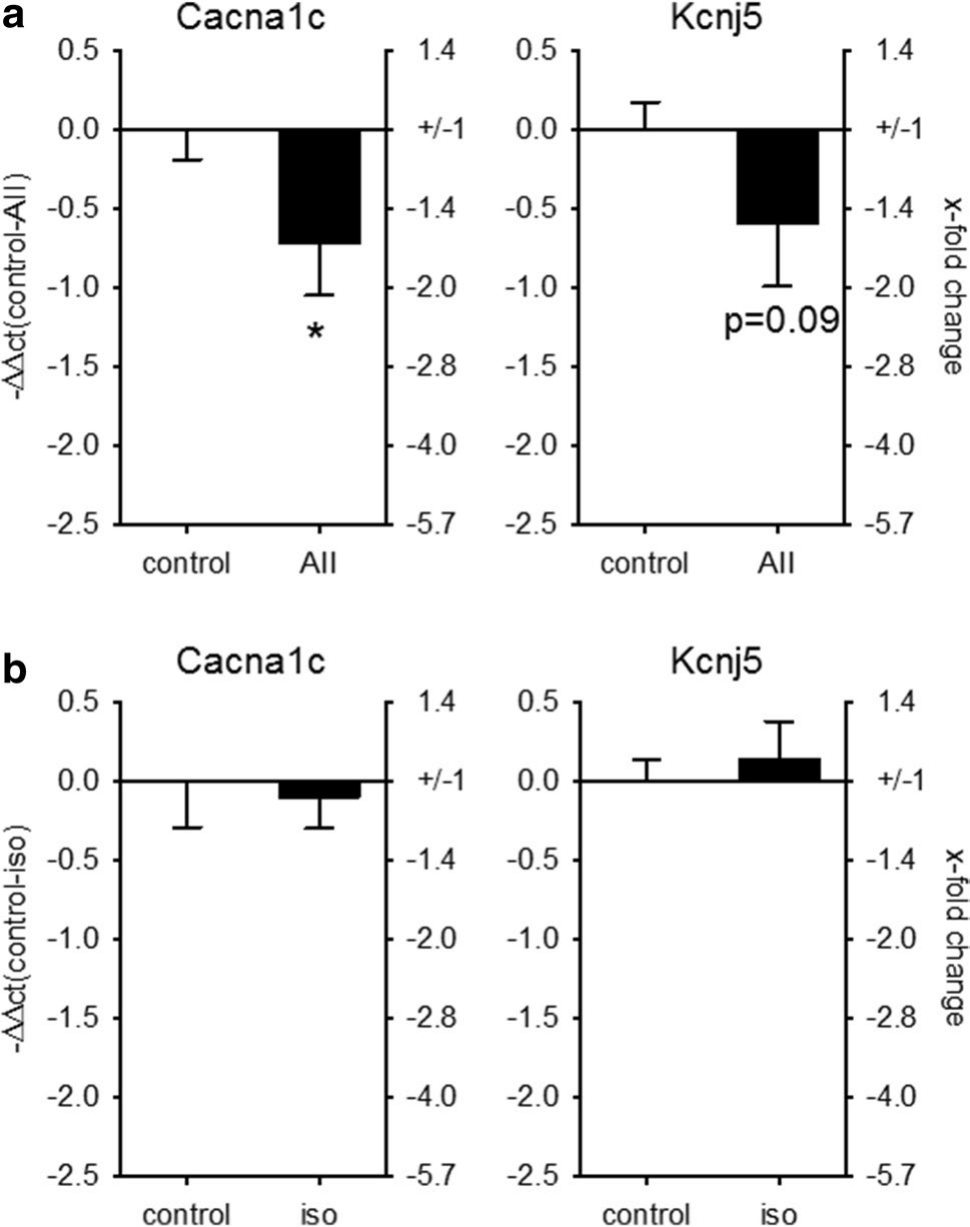
Cacna1c mRNA correlates with the change in miR-221/222 expression in AII-treated animals. To test if the increase in miRNAs is correlated to the changes in Cacna1c and Kcnj5 expression, we performed real-time qRT-PCR in the two mouse models with pharmacologically induced heart hypertrophy. While in AII-treated animals, Cacna1c was downregulated, there was no change in Cacna1c in isoprenaline-treated animals (**a**) AII: *N* = 12–15 animals/group, **b** isoprenaline: 5–6 animals/group (relative change compared to control)

To test if miR-221/-222 might directly target the five aforementioned ion channel subunits in the heart, 3′-UTR luciferase assays were performed. As isolated adult cardiomyocytes dedifferentiate rapidly in culture, further analyses of ion channel regulation by miR-221/222 were performed in HL-1 or HEK293 cells. Due to the length of the Cacna1c and the Cacna2d1 3′-UTR, the sequence was divided into three or two fragments, respectively. Figure 5a, b shows the effect of miR-221 or miR-222 on the luciferase activity of the 3′-UTR of Cacna1c, Cacnb2, Cacna2d1, Kcnd2, and Kcnj5 in HEK293 cells. Neither miR-221 nor miR-222 mimics reduced the luciferase activity of the 3′-UTRs from Cacna2d1, thereby indicating that these two miRs might not target this mRNA by direct binding to its 3′-UTR in the heart. miR-221 mimics reduced the luciferase activity of the 3′-UTR for Cacna1c and Kcnj5 (Fig. 5a), while miR-222 reduced the luciferase activity of the 3′-UTR of Cacna1c, Cacnb2, Kcnj5, and Kcnd2 (Fig. 5b). This indicates that the miR-221/222 cluster targets L-type Ca^2+^ channel subunits and potassium channel subunits, namely Cacna1c, Cacnb2, Kcnj5, and Kcnd2, via their 3′-UTR. As a proof of principle, we evaluated the effect of miR-221 mimics in HL-1 cardiomyocytes. While Cacna2d1 and Kcnj5 mRNA levels were not altered, miR-221 mimic reduced the mRNA amount of Cacnb2 and Cacna1c (Fig. 5c). To demonstrate that increased miR-221 and -222 amounts might have a functional impact, ion currents and protein content were analyzed. As the Cacna1c subunit represents the pore forming *α* subunit of the L-type Ca^2+^ channel, we analyzed the effect of miR-221 and -222 mimics on L-type Ca^2+^ current (I_Ca,L_) density by whole-cell patch clamp recording. Transfection with mimics for both miRNAs decreased the current density of the L-type Ca^2+^ channel in HL-1 cells significantly (Fig. 5d). Furthermore, miR-221 mimics reduced the protein expression of GIRK4 but not of GIRK1, determined by Western blot analysis (miR-221 mimic 64.2 ± 2.1% of control, *N* = 3 independent experiments, Fig. 5e). To determine if the change in GIRK4 protein content results in a reduced ion flux through GIRK1/4, we analyzed HL-1 cells transfected with either scrambled, miR-221 or -222 mimics with a digital high-content fluorescence microscope and a thallium-sensitive dye. Upon stimulation with carbachol, the increase in thallium-dependent fluorescence was reduced by miR-221 and -222 but not by control mimics (Fig. 5f). As this current is tertiapin q-sensitive (Supplementary Figure S2), we conclude that Kcnj5 or GIRK4 is a target for miR-221 and -222. Together, these data suggest that miR-221 and -222 may impair action potential generation in cardiomyocytes of the sinoatrial node, electromechanical coupling in the working myocardium, and reduce the influence of the parasympathetic nervous system on the heart rate. Additionally, we could confirm that in mice treated with AII, where cardiac miR-221 and -222 were upregulated, the mRNA for Cacna1c was reduced (Fig. 6a). In contrast, no change in Cacna1c or Kcnj5 mRNA could be observed in mice with heart hypertrophy but without upregulation of the two miRs (isoprenaline treatment, Fig. 6b).

## Discussion

Although an impact of miRNAs on cardiac excitation generation and propagation is widely accepted [29, 33], the knowledge is still incomplete. In the present study, we provide evidence for a role of the clustered miR-221 and miR-222 in cardiac electrophysiology with potential pathophysiological relevance. These miRs have been reported to be altered during cardiac diseases (for review see [19]) without further information regarding a potential causal role. In this study, we took advantage of a mouse model with severe heart hypertrophy but preserved ejection fraction. No signs of decompensation or ischemia were observed in these hearts [17], in contrast to studies that were performed at the stage of heart failure [9, 34].

The increase in miR-221 expression in our mouse model is in good agreement with data from human patients with hypertrophic cardiomyopathy after myocardiectomy, where miR-221 expression also increased twofold [6]. Additionally, we demonstrate that in the pathological highly relevant situation of an over-activated renin–angiotensin–aldosterone system (AII-induced heart hypertrophy), the increase in heart hypertrophy is associated with an increase in miR-221 and -222. In contrast, adrenergic stimulation and the subsequent increase in heart weight do not induce a substantial rise in miR-221/222 expression. Taken together, these data indicate that hypertrophy alone is not sufficient to increase miR-221/222 expression but that increased miR-221/222 levels are associated with certain forms of hypertrophy, e.g., an over-activated renin–angiotensin–aldosterone system. The molecular mechanisms leading to an increase in the expression of these miRs need to be evaluated in a further study. In the study of Verjans et al. [31], incubation of fibroblasts with TGF*β*1 decreases the expression of miR-221/222, while in tumor cells the EGFR seems to increase the expression of these miRNAs [35]. From our findings, we conclude, at the moment, that in cardiomyocytes, angiotensin II increases the expression of those miRNAs in an EGFR-independent way. If this increased expression of miR-221/222 is due to an alteration of TGF*β*1 by angiotensin II in fibroblasts needs to be evaluated in further studies.

Additionally, we were able to confirm that the observed increase in miR-221/222 amount is accompanied by reduced expression of a known target, namely p27 [6]. Although this does not prove a direct interaction, it supports the functional relevance of the miR-221/222 expression changes. Comparing the copy numbers of the miR-221/222, p27 as well as Cacna1c, Kcnd2, and Kcnj5 mRNA by ddPCR shows that the change in protein levels of the mentioned genes might be caused by those two miRNAs without supporting factors. But this has to be evaluated in more detail. Furthermore, the doubling in copy number for the miRs in the hypertrophied heart does not lead to a decreased copy number of p27 mRNA, arguing for a post-transcriptional mechanism leading to the decrease in p27 protein levels. Of note, the reduced amount of p27 could at least partially explain the cardiac hypertrophy, as it has been demonstrated that mice with deletion of p27 develop heart hypertrophy with increasing age [36]. And miR-221 as well as miR-222 has been reported to reduce cardiomyocyte autophagy by reduction of p27 [37, 38].

miR-221 and -222 share the same seed sequence and derive from a single pri-miR [39], but there are hints that the regulation of both miRs differs [40, 41]. miR-222 was described as mainly expressed in cardiac fibroblasts [31, 34]. In contrast to the above mentioned studies, we could not observe an increase in miR-221 or miR-222 in cardiac fibroblasts of mice with genetic heart hypertrophy. This might be due to the fact that in our mouse models, no signs of heart failure could be observed, e.g., lung weight per tibia length, a measure for lung congestion, was not altered [17, 42].

Comparison of downregulated mRNAs with predicted targets of miR-221 and -222 revealed 261 protein coding RNAs that are predicted as potential targets of miR-221 and -222. Of 460 downregulated protein-coding mRNAs, this would be ~ 57%, a very high fraction. To test if this might be an unspecific correlation, we tested how many of the upregulated, protein-coding RNAs are predicted targets of miR-221/222. This applies only for 24 out of 399 protein-coding RNAs, a fraction of about ~ 6%. Therefore, we think that our strategy can serve as a first approach to identify targets for further validation. Among the downregulated protein-coding RNAs, an enriched subset was related to excitation generation and conduction according to G:profiler and GOrilla. Included in the group of enriched genes were the subunits of the L-type Ca^2+^ channel (Cacna1c, Cacnb2, Cacna2d1), the G-protein-activated inwardly rectifying potassium channel 4 (Kcnj5), and a voltage-gated potassium channel (Kcnd2). These three ion channels are involved in the electrical potential generation and propagation of the heart. The L-type Ca^2+^ channel is important for electromechanical coupling in the working myocardium and underlies the slow upstroke of the sinoatrial node potential. A decrease in the expression of this ion channel, most probably, would reduce the heart rate, cardiac force development, and action potential duration. Kcnj5 belongs to the G-protein-activated inwardly rectifying potassium channel family and mediates the parasympathetic stimulation via the muscarinergic M2-receptor in the electrical conduction system of the heart, and thereby a reduction in heart frequency. Kcnd2 is part of the potassium voltage-gated channel subfamily D. It contributes to the early repolarization of the action potential in the working myocardium. A reduction in the current would most probably increase action potential duration. However, one has to take into account, as the heart beat is of viable importance, compensatory mechanisms will most probably mask the pure electrophysiological properties of the reduced ion channel currents, therefore making it highly complicated to deduce ion currents from ECG recordings. As our mice showed alterations in the ECG, we chose to evaluate this subgroup of genes. While miR-221 binds to the 3′-UTR of Cacna1c and Kcnj5, miR-222 reduces the luciferase activity for the reporter plasmids for Cacna1c, Cacnb2, and Kcnj5. The L-type Ca^2+^ channel is the main Ca^2+^ channel of the conduction system and the working myocardium. This current is responsible for the slow upstroke in the sinoatrial node and the generation of the plateau in the working myocardium [43, 44]. In the heart, this channel is composed of three subunits: (1) the pore-forming subunit *α*1, which regulates the main biophysical and pharmacological properties and is encoded by the Cacna1c gene, and two auxiliary subunits, including (2) a cytoplasmic *β* subunit, encoded by Cacnb2 and (3) *α*2*δ* encoded by Cacna2d [43]. From the *β*-subunits, the *β*2 isoform, encoded by Cacnb2 is the dominant one in the heart [45]. The *β* subunit as well as the *α*2*δ* subunit is required for anchoring, trafficking, and regulatory functions [43]. We were able to demonstrate that both miRNAs bind to the 3′-UTR of the Cacna1c subunit and lead to a reduced L-type Ca^2+^ current density in cardiomyocytes as demonstrated in HL-1 cells by whole-cell patch clamping. Additionally, in the hearts of EGFR^Δ/ΔVSMC&CM^, mice the mRNA amount for Kcnip2 (NGS) is downregulated. It has been demonstrated that the corresponding protein increases the L-type Ca^2+^ channel density [46] in murine cardiomyocytes. As this mRNA is also a predicted target for miR-221/222, future studies will have to detect a possible molecular interaction.

Activation of the L-type Ca^2+^ channel enhances Ca^2+^ influx from the extracellular space supporting the action potential generation in the conduction system of the heart [44]. Antagonizing the L-type Ca^2+^ channel has been shown to prevent pathological cardiac remodeling and hypertrophy in animal models [47, 48]. But a reduction of I_Ca,L_ can also be detrimental. Homozygous deletion of the *α*1 subunit of the L-type Ca^2+^ channel causes embryonic death before day 14.5 in mice [49] and even heterozygous deletion results in cardiac hypertrophy and ventricular dilatation by pathological or physiological cardiovascular stress [44, 50]. There is increasing evidence that miRs might be involved in the regulation of this ion channel. It has been suggested that besides miR-221 and -222, also miR-208b [29], miR-29a-3p [51], and miR-21 [52] bind to the 3′-UTR of Cacna1c, and thereby reduce I_Ca,L_. From these miRs, only miR-208b was altered in EGFR^Δ/ΔVSMC&CM^ mice. Additionally, Cacnb2 is a confirmed target for miR-21 [52], miR-132, and miR-222 [53]. As the dysfunction of the L-type Ca^2+^ channel is involved in different human forms of arrhythmia, like the Brugada syndrome with or without short QT interval, Timothy syndrome and even in arrhythmias of patients with myotonic dystrophy one and two [44, 54], the regulation of its subunits by miRs is of clinical importance. As mentioned in “Materials and methods” section, after verifying that miR-221 does not change the current density–voltage relationship, we obtained maximum peak inward current by stepping from a holding potential to various test potentials (15–30 mV). To increase the current amplitude and to facilitate patch formation, we raised the extracellular calcium concentration in our experiments to 10 mM. As a consequence of the high extracellular calcium concentration, the maximum peak inward current is shifted from 0 mV to the potential range identified by us in former experiments (15–30 mV). Similar observations have been published in the literature, e.g., [27, 55]. Since we were only interested in the maximal peak I_Ca,L_ to confirm the functional relevance of reduced channel expression and not primarily in discrete alterations of the voltage dependence, we restricted our current measurements mainly to this potential range. In our hands, HL-1 cells do not tolerate repeated depolarizations very well, therefore we reduced the “normal” I–V curve, starting at about − 20 mV, to four depolarizations between 15 and 30 mV as described. We abstained from using nifedipine as L-type channel blocker, because at 10 mM [Ca^2+^]_o_, a high concentration of nifedipine would have to be applied leading to non-specific effects of the drug [56, 57].

Kcnj5 provides one subunit of the G-protein-activated inwardly rectifying potassium channel GIRK1/4 or I_K,ACh_ channel (Kir3.1/3.4). The Kir3.x ion channel family consists of four members: Kcnj3 (GIRK1), Kcnj5 (GIRK4), Kcnj6, and Kcnj9 [58]. They form homo- or hetero-tetramers in various combinations. All combinations are activated by *βγ*-subunits of G-proteins. In the heart, the main hetero-tetramer is GIRK1/4 but also homo-tetramers of GIRK4 have been described in the atria [59]. Without GIRK4 in the heart, a functional I_K,Ach_ channel cannot be built [60, 61]. Because the open probability of this channel is increased by binding of acetylcholine to the muscarinergic M2-receptor, it mediates a part of the parasympathetic effects in the heart. Mice with deletion of GIRK4 lose the parasympathetic induced heart rate variability [62]. Herein, we demonstrate that miR-221/222 target the Kcnj5 3′-UTR, and thereby reduce the protein amount and the ion current in HL-1 cells. GIRK1 (Kcnj3) is also a predicted target of miR-221/222, but we were not able to induce a protein reduction of this ion channel by transfection of HL-1 cells with miR-221. But further studies with, e.g., longer incubation periods have to validate this finding.

In summary, alteration of miR-221 and -222 expression can contribute to changed L-type Ca^2+^ channel density, GIRK1/4 density, and, perhaps, Kir2.1 density resulting in slower excitation propagation and possibly disturbed electromechanical coupling, prolonging the QT interval, and thereby making the heart more vulnerable to arrhythmias. We do not provide direct evidence for the alteration of the electrical properties of the heart by miR-221/222, but with the in vitro data from the HL-1 cells, we demonstrate that miR-221/222 impact the ion channels in the cardiomyocytes. As these miRNAs are mainly produced in the fibroblasts [31, 34], the question remains if the miRNAs from the fibroblasts, as described for miR-21-3p by the working group of Thomas Thum [63], impact the ion channels in the cardiomyocytes. In the genetic model for heart hypertrophy, we did show that in the isolated adult cardiomyocytes, the miRNAs were increased but not in the fibroblasts. If this is also true for model of pressure overload induced by angiotensin II needs to be analyzed further. Verjans et al. [31] did show that in their model of pressure overload, heart fibrosis and heart failure were induced and that miR-221/222 in fibroblasts increased the expression of mRNAs leading to cardiac fibrosis. In our model, we used a reduced amount of angiotensin II and a shorter time period to prevent heart failure. Therefore, it is possible that in the non-failing heart, miR-221/222 promote electrical remodeling, inhibit cardiac fibrosis by influencing the protein expression in cardiomyocytes, and thereby might preserve cardiac function. This has to be evaluated in further studies. As well as the overall effect of miR-221/222 on action potential generation and propagation in cardiomyocytes with respect to the integrated reduction of at least L-type Ca^2+^ channel and GIRK4 have to be further studied. One of the limitations of our study is the lack of action potential recordings in HL-1 cells. In our hands, this approach encounters major difficulties for three reasons: (1) only a subpopulation of HL-1 cells are continuously beating, and therefore spontaneous action potential recordings can be measured only from a fraction of cells. (2) L-type Ca^2+^ channel is heterogeneously expressed in HL-1 cells. (3) Only about 10–20% of HL-1 cells express Kcnj5 channels. Therefore, we believe that HL-1 cells are not a suitable tool to record action potentials and electromechanical coupling. We are currently establishing the neonatal cardiomyocyte preparation of mice and will in the future work with freshly isolated cardiomyocytes from adult mice treated with antagomirs.

In summary, our findings and the findings from Verjans et al. [31] as well as Su et al. [37, 38] indicate an autophagy- and electrical remodeling-supporting effect in cardiomyocytes but fibrosis-inhibiting effect in fibroblasts of miR-221/222, whereby the effect could be overall supportive for heart function, as left ventricular dysfunction and dilatation are aggravated in the angiotensin II model by inhibiting the miRNAs.

### Electronic supplementary material

Below is the link to the electronic supplementary material.

Supplementary material 1 (DOCX 124 kb)

Supplementary material 2 (PDF 473 kb)

Supplementary material 3 (XLSX 8703 kb)

Supplementary material 4 (XLSX 111 kb)

## Abbreviations

ANOVA: Analysis of variance
AUC: Area under the curve
AII: Angiotensin II
Bp: Base pair
BW: Body weight
Cacna1C: Calcium voltage-gated channel subunit *α*1 C, L-type
Cacnb2: Calcium voltage-gated channel auxiliary subunit *β*2
Cacna2d1: Voltage-dependent calcium channel subunit *α*2/*δ*1
Cav1.2: L-type Ca^2+^ channel
CCH: Carbachol, acetylcholine analog
cDNA: Complementary DNA
CE: Carbachol effect
CM: Cardiomyocyte
ddPCR: Droplet digital PCR
DMEM: Dulbecco’s modified Eagle medium
ECG: Electrocardiography
EGFR: Epidermal growth factor receptor
FAM: Fluorescein amidite
FCS: Fetal calf serum
FPM: Fragments per million
Gapdh: Glyceraldehyde 3-phosphate dehydrogenase
GIRK1: G-protein inwardly rectifying potassium channel 1
GIRK4: G-protein inwardly rectifying potassium channel 4
HEK293: Human embryonic kidney cells
HEX: Hexachloro-fluorescein
HL-1: Atrial tumor-derived, cardiomyocyte cell line
HSP90: Heat shock protein 90
HW: Heart weight
I_Ca,L_: L-type Ca^2+^ channel current
IgG: Immunoglobulin G
I_KACh_: Acetylcholine-induced potassium current
iso: Isoprenaline
Kcnd2: Potassium voltage-gated channel subfamily D member 2
Kcnj3: Potassium voltage-gated channel subfamily J member 3
Kcnj5: Potassium voltage-gated channel subfamily J member 5
Kcnj6: Potassium voltage-gated channel subfamily J member 6
Kcnj9: Potassium voltage-gated channel subfamily J member 9
KO: Knockout
Kir3.1/3.4: G-protein-activated inwardly rectifying potassium channel Kir3.1 and 3.4
Kv4.2: Voltage-gated potassium channel
mRNA: Messenger RNA
miR: MicroRNA
M2: Muscarinergic receptor type 2
NGS: Next generation sequencing
NN: Normal-to-normal intervals
pAT1R: Human angiotensin II receptor type 1 containing plasmid
PBS: Phosphate buffered saline
PVDF: Polyvinylidenfluorid
p27: Cyclin-dependent kinase inhibitor 1B
qRT-PCR: Quantitative reverse transcription PCR
RNA-seq: RNA-sequencing
RT: Reverse transcription
RMSSD: Square root of the mean of the sum of the squares of differences between adjacent NN intervals
SDδ: Standard deviation of averages of normal R–R intervals
SEM: Standard error of mean
SDNN: Standard deviation of R–R interval of normal-to-normal
TGFβ1: Transforming growth factor *β*1
TI^+^: Thallium ion
TL: Tibia length
TQ: Tertiapin Q
VSMC: Vascular smooth muscle cell
WT: Wild type
18S: 18S ribosomal RNA
3′-UTR: 3′-untranslated region
