# Supplementary material for: miR-221 and -222 target CACNA1C and KCNJ5 leading to altered cardiac ion channel expression and current density
Source: Cell Mol Life Sci. 2019 Jul 16;77(5):903–18. doi: 10.1007/s00018-019-03217-y (PMC7058603; doi:10.1007/s00018-019-03217-y)
Supplement: Supplementary file 1 — Supplementary material 1 (DOCX 124 kb) [file 18_2019_3217_MOESM1_ESM.docx]

**Supplementary Data**

Stephanie Binas^1§^, Maria Knyrim^1§^, Julia Hupfeld^1^, Udo Kloeckner^1^, Sindy Rabe^1^, Sigrid Mildenberger^1^, Katja Quarch^1^, Nicole Strätz^1^, Danny Misiak^2^, Michael Gekle^1#^, Claudia Grossmann^1#^, Barbara Schreier^1#*^

**MIR-221 AND -222 TARGET CACNA1C AND KCNJ5 LEADING TO ALTERED CARDIAC ION CHANNEL EXPRESSION AND CURRENT DENSITY**

^1^Julius-Bernstein-Institute of Physiology, Martin Luther University Halle-Wittenberg, Magdeburger Str. 6, 06110 Halle/Saale, Germany,

^2^Institute of Molecular Medicine, Martin-Luther-University Halle-Wittenberg, Heinrich-Damerow-Str. 1, 06120 Halle/Saale, Germany

§ Stephanie Binas and Maria Knyrim contributed equally to this work.

# Barbara Schreier, Claudia Grossmann & Michael Gekle contributed equally to this work.

Short Title: miR-221/222 and cardiac ion channels

*Corresponding Author

Barbara Schreier

Martin Luther University Halle-Wittenberg

Julius-Bernstein-Institute of Physiology

Magdeburger Str. 6

06110 Halle/Saale

Germany

Tel: ++493455571886

Fax: ++493455574019

E-mail: barbara.schreier@medizin.uni-halle.de

**Supplementary Table S1**: Primers used for qPCR.

| **Gen** | **Protein** | **Ref. Seq.** | **Sense Primer (5’-3’)** | **Antisense Primer(5’-3’)** | **Annealing Temp**  **[°C]** | **Product [bp]** |
| --- | --- | --- | --- | --- | --- | --- |
| 18 S | - | NR_003278.3 | GTAACCCGTTGAACCCCATT | CCATCCAATCGGTAGTAGCG | 62 | 150 |
| Gapdh | GAPDH | NM_002046.4 | AAGGTGAAGGTCGGAGTCAA | AATGAAGGGGTCATTGATGG | 60 | 107 |
| Cdkn1B | Cyclin-dependent kinase inhibitor 1B | NM_009875 | GCGGTGCCTTTAATTGGGTC | GTTCGGGGAACCGTCTGAAA | 57 | 198 |
| EGFR | epidermal growth factor receptor | NM_207655.2 | GACCTTCACATCCTGCCAGT | GCATGGAGGTCAGTCCAGTT | 63 | 155 |
| Cacnb2 | L-type Ca^2+^ channel, subunit β2 | NM_001252533.1 | GCGAGGCAAAGCCACCTTTA | CTGTTCGTGCTGTAGCCTCA | 60 | 492 |
| Cacna1c | L-type Ca^2+^ channel, subunit α1 | NM_009781.4 | CCTGCTGGTGGTTAGCGTG | TCTGCCTCCGTCTGTTTAGAA | 60 | 285 |
| Cacna2d1 | L-type Ca^2+^ channel, subunit 2Δ1 | NM_001110843.1 | GGCCAGATATTCAGAAACCCTGA | TCTCCGGCCTCTTTGGGATA | 63 | 327 |
| Kcnd2 | Kv4.2 | NM_012281.2 | CACTCTCAAGGGCTGCGTAT | TCGTTTGTCTGCTCGTTGGT | 60 | 351 |
| Kcnj5 | GIRK4 (Kir3.4) | NM_010605.4 | ATCTCCAGAAGTTAGCCCCAA | CATGCTCCCAAGTACACCCT | 60 | 105 |

**Supplementary Table S2**: Taqman^TM^ Assays used for miRNA detection.

| **Taqman Assay** | **Order ID** | **miRBase Accession Nr** |
| --- | --- | --- |
| hsa-miR-221 | 000524 | MIMAT0000278 |
| mmu-miR-221* | 464489 | MIMAT0017060 |
| hsa-miR-222 | 002276 | MIMAT0000670 |
| U6 snRNA | 001973 |  |

**Supplementary Table S3: Dual Luciferase reporter gene constructs obtained from GeneCopoeia containing the 3’-UTRs of the L-type Ca^2+^ channel subunits with the respective RefSeq gene accession numbers.**

| **Reporter construct** | **Length 3´-UTR** | **RefSeq** |
| --- | --- | --- |
| pEZX-MT06-Cacnb2 | 1832 bp | NM_001252533.1 |
| pEZX-MT06-Cacna1c_I | 2228 bp ( -16 - 2211 bp ) | NM_001159533.2 |
| pEZX-MT06-Cacna1c_II | 2322 bp ( 2143 - 4464 bp ) | NM_001159533.2 |
| pEZX-MT06-Cacna1c_III | 2299 bp ( 4393 - 6691 bp ) | NM_001159533.2 |
| pEZX-MT06-Cacna2d1_I | 1894 bp ( -1 - 1892 bp ) | NM_001110843.1 |
| pEZX-MT06-Cacna2d1_II | 2000 bp ( 1844 - 3843 bp ) | NM_001110843.1 |
| pEZX-Kcnd2 | 2462 bp | NM_019697.3 |
| pEZX-Kcnj5 | 2797 bp | NM_010605.4 |

**Supplementary Figure S1: Current density curve for miR-221, miR-222 and scramble transfected HL-1 cells**. **a)** HL-1 cells were transfected either with miR‑221 or scramble-miRNA (10 µg/ml, N = 4-5 cells/group) or **b)** miR-222 or scramble-miRNA (10 µg/ml N = 8-9 cells/group) for 48h before current recordings was performed in the whole cell configuration of the patch clamp technique. By integrating the capacitive current at the end of a 10 ms long voltage step (-80 mV to -70 mV) the input capacitance of the cells was obtained. This value was normalized to the peak amplitude of the inward current to obtain the current density (pA/pF) in order to compensate for differences in cell size. To measure solely L-Type Ca^2+^-currents we used a holding potential of -40 mV (miR-221 and corresponding scramble transfected cells) or -35 mV (miR-222 and corresponding scramble transfected cells). HL-1 cells were depolarized every 8 s for 100 ms from the holding potential to various test potentials (-30 mV to 60 mV in 10 mV increments). As the current density for L-Type Ca^2+^ channel was reduced by miR-221 and miR-222 in HL-1 compared to scramble, but the current density – voltage relationship was the same, in following experiments only the peak current was determined. N =4-5 cells/group, *p<0.05 miR-221 compared to scramble.


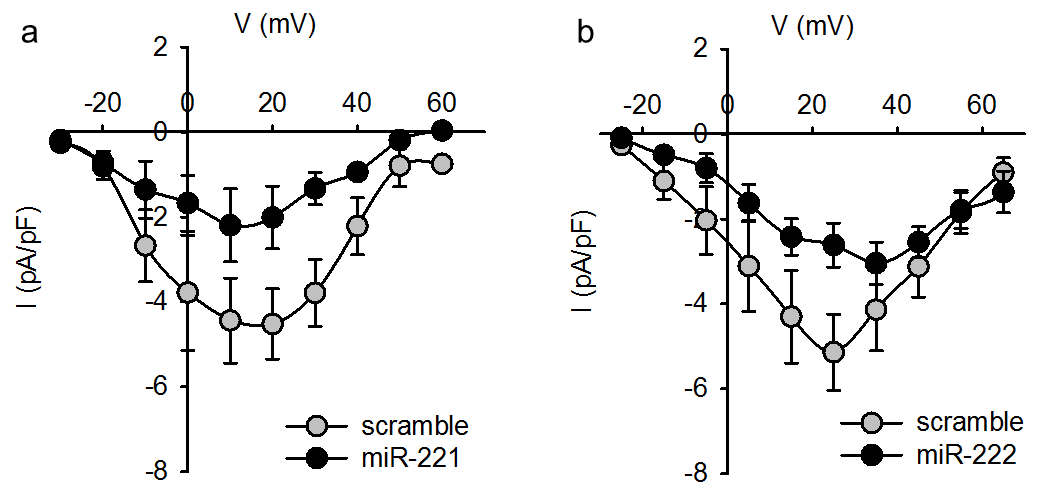


**Supplementary Figure S2: Validation of Fluxor Assay for GIRK1/4.** Thallium ion flux was measured in HL-1 cells with or without carbachol (10µM) and tertiapin q (100 nM). Fluorescence intensity was normalized to time point 0 (A) and to the mean fluorescence ratio (F/F_0_) of the controls (B), thereby demonstrating that the carbachol induced thallium flux is tertiapin q sensitive and therefore GIRK dependent. (N=4 experiments/group, n=3 wells/experiment)

**Supplementary Figure S3: Lung weight/tibia length and interstitial fibrosis in the pharmacological models.** Lung weight/ tibia length was not different in angiotensin II (AII, a) or isoprenaline (Iso, b) treated animals compared to respective untreated animals. While in AII treated animals no increase in Sirius red stained tissue (% interstitial fibrosis, c) could be observed, there was a significant increase due to isoprenaline treatment (d). (AII: N= 12-15 animals/group, iso: 5-6 animals/group)


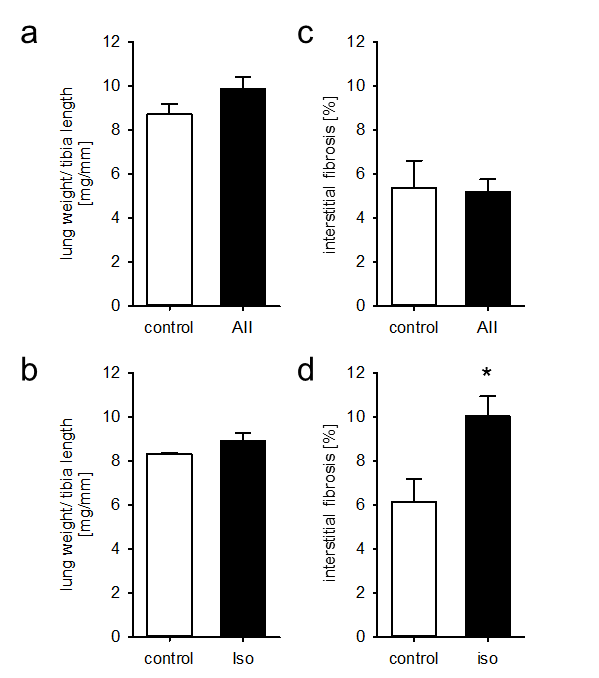


**Extended**

**Material and Methods**

**Ethics statement**

All mouse experiments described in this manuscript were approved by the local government (Landesverwaltungsamt Sachsen-Anhalt, Germany, permit number:42502-2-1124 and -1201 MLU) and were performed according to the guidelines of the directive 2010/63/EU of the European Parliament on the protection of animals used for scientific purposes.

**Animal models**

Mice were kept in the facilities of the University of Halle-Wittenberg at a room temperature of 20 ± 1°C and with a 12 h/12 h light/dark cycle. Generation, genotyping and the cardiovascular phenotype of EGFR KO animals were described before [5]. Mice with a deletion of the EGFR in VSMC and a strong reduction in cardiomyocytes are termed either EGFR^Δ/ΔVSMC&CM^ or knockout [KO]. Prior to angiotensin II [AII] or isoprenaline [iso] treatment, animals where observed for 7 days and weight was measured daily.

For angiotensin II (AII, 1000 ng/kg BW/min over three weeks) or isoprenaline (iso, 30 mg/kg/day or two weeks) treatment animals were anaesthetized with isoflurane (~2% v/v in 100% O2, 1 l/min) and Alzet minipumps (1004) were implanted subcutaneously in the back of the animals. 5-10 animals per group were included into the study. Carprofen (5-10 mg/kg BW, Rimadyl, Pfizer, New York, USA) was injected subcutaneously immediately before pump implantation. If necessary, pain relief was repeated every twelve hours. Mice were sacrificed by cervical dislocation in isoflurane anesthesia.

**Harvesting of organs, cardiomyocytes and cardiac fibroblasts**

Mice were sacrificed after anesthesia with isoflurane as described above*.* Immediately after death heart, aorta, lung and kidney were removed, cleaned from connective tissue and weighed. Tibia length was measured for normalization of organ weights. Furthermore, organs were immediately frozen in liquid nitrogen and a part of the heart was stored in 4.5% paraformaldehyde for fixation. Hearts selected for preparation of cardiomyocytes and cardiac fibroblasts where perfused with digestion buffers as described before [3]. Freshly isolated cardiomyocytes were directly frozen in liquid nitrogen and cardiac fibroblasts were cultured to increase the amount of cells.

**Microscopic analysis of the hearts**

The degree of interstitial fibrosis in hearts was determined by evaluation of Sirius Red stained area from at least 10 microscopic fields per animal, utilizing a point counting technique [6]. To analyze cardiomyocyte diameter hematoxylin/eosin stained cross-sections of the left chamber were used. At least 100 cardiomyocytes were analyzed per mouse.

**Electrocardiography**

Animals were anesthetized with isoflurane 2% (1L/min O2) and electrocardiography recordings were obtained for at least ten minutes employing AD Instruments LabChart equipment and an Einthoven I limb lead recording. The following parameters were analyzed for standard ECG evaluation: PR interval (measured from the beginning of the P wave to the start of the chamber complex, a measure for the conduction time), P duration (time from start to end of the P wave, a measure for the dispersion of the excitation in the atria), QRS interval (time from start to end of the chamber excitation, a measure for the dispersion of the excitation in the chambers) and QTc interval (time from start of the chamber excitation to the end of the chamber repolarization, heart rate corrected according to Bazette, QTc = QT interval/√RR-interval). Heart rate variability was analyzed in the above mentioned ECG as described before [7] with ΔNN=6ms. The following parameters were analyzed: SDNN (standard deviation of RR interval of normal-to-normal intervals, a representation of overall heart rate variability), SD delta NN (standard deviation of averages of normal R-R intervals), RMSSD (Square root of the mean of the sum of the squares of differences between adjacent NN intervals).

**RNA isolation and concentration determination**

Total RNA was isolated using the InviTrap spin tissue RNA mini kit (STRATEC Molecular GmbH, Berlin, Germany) by following their instructions. RNA concentration was measured by NanoVue Plus spectrophotometer (GE Healthcare, Berlin Germany). For RNA seq, total RNA was isolated by TRIzol Reagent (Invitrogen Life Technologies, Darmstadt, Germany). RNA was precipitated twice with ethanol and the quality was analyzed on a 2100 Bioanalyzer (Agilent). All samples had a RNA Integrity Number (RIN) above 7 (RIN 10 indicates maximum quality of the samples).

**cDNA preparation**

To remove genomic DNA contamination, one µg of total RNA was treated with 2.8 units of DNase I (RNase-free) (New England Biolabs, Frankfurt am Main, Germany) at 37°C for one hour, followed by enzyme inactivation at 75°C for 10 min. Reverse transcription (RT) reaction of DNase I-treated total RNA was performed with random primers using SuperScript II reverse transcriptase (Invitrogen, Life Technologies, Darmstadt, Germany), according to the kit’s manual.

**Real-time RT-PCR**

To confirm differential expression of genes 1 µl cDNA was used in real-time RT-PCR (CFX96 Touch Real-Time PCR Detection System, BioRad, Munich, Germany or 7900HT Fast Real-time PCR system, Applied Biosystems, via Thermo Fisher Scientific, Karlsruhe, Germany). After initial denaturing at 95°C for 10 min, cDNA was amplified by 40 cycles of 95°C for 15 sec, 60°C for 30 sec, 72°C for 20 sec, followed by 72°C for 5 min to allow complete extension. Gene expression was normalized to the expression levels of eukaryotic 18S rRNA or Gapdh. Sequence of primers, as well as annealing temperature and RefSeq accession number/id are given in Supplementary table S1.

**Droplet Digital PCR**

To determine the absolute amount of RNA, Droplet Digital PCR (ddPCR) was performed using the QX200 system of BioRad (Munich, Germany). cDNA was prepared as described above and used in ddPCR at the same conditions as in real-time RT-PCR.

**TaqMan PCR**

For the TaqMan PCR, cDNA was prepared as above. A primer pair and a FAM-labeled probe specific for either miR-221 or miR-222 was used simultaneously with a primer pair and a HEX-labeled probe specific for U6 and TaqMan™ Universal PCR Master Mix, no AmpErase™ UNG (Applied Biosystems, via Thermo Fisher Scientific, Karlsruhe, Germany). Thermal conditions: initial 95°C for 10 min followed by 40 cycles of 95°C for 15 sec and 59°C for 60 sec. The list of TaqMan™ assays purchased from Applied Biosystems is given in supplementary table S2.

**Next generation sequencing of mRNA and cluster analysis**

Total RNA was isolated as described above. Sequencing was performed with an Illumina HiScanSQ at the Core Unit DNA Technologies of the Medical Faculty, University Leipzig (Liebigstraße 21, 04103 Leipzig, Germany). Libraries were prepared with indexed adapters, and clusters were generated on the cluster flow cells. cDNA fragments were hybridized to the lawn of complementary primers followed by „bridge amplification“. Paired-end sequencing was performed by synthesis (SBS) via reversible terminator-based method. A quality check was performed on raw data (fastQC) before quality trimming (cutadapt) and adapter clipping. Mapping was performed by TopHat2, using Bowtie2 and Samtools. Subsequently, quality was checked again prior to counting and annotation (R).

In order to analyze the data, we created two-dimensional groups of significant changes according to fold change FC and abundance (fragments per million; FPM) for upregulated and downregulated genes. mRNA enrichment analysis was performed by g:Profiler [4] and Gorilla [2]. Additional analysis of TF-site enrichment within promotors of DE mRNA groups (regions -950 to +50 and -450 to +50) was performed with Pscan [9]. Analysis was corrected for multiple testing as described for the corresponding tools.

**Next generation sequencing of microRNA**

Total RNA was isolated as described above. Sequencing was performed as described previously [1] at the Core Unit DNA Technologies of the Medical Faculty, University Leipzig (Liebigstraße 21, 04103 Leipzig, Germany). 500 ng of RNA from each sample was used in the small RNA protocol with the TruSeq™ Small RNA sample prepkit v2 (Illumina) following the instructions of the manufacturer. The barcoded libraries were size restricted between 140 and 165 base pairs for additional enrichment of miRs, purified and quantified using the Library Quantification Kit-Illumina/Universal (KAPA Biosystems, Woburn, MA, USA) according to the instructions of the manufacturer. Sequencing of 50 base pairs was performed with an Illumina HighScan-SQ sequencer using version 3 chemistry and flow cell following the instructions of the manufacturer. The R packages DESeq2 and EdgeR were used for normalization and to calculate differential expression of miRs.

**HL1 cell line**

HL-1 cells were maintained in Claycomb medium (Sigma, Munich, Germany) with the following supplements: 10% FCS (Biochrom, Berlin, Germany), 2 mM L-glutamine (Sigma ), 100 µM noradrenaline (Sigma), 100 U/ml penicillin and 100 µg/ml streptomycin (Sigma). Cells were sub cultured at confluency and medium was changed twice a week.

**Transfection**

HL-1 cells were transfected with 30 nM of miRCURY LNA miR-221 mimics or mimic negative control (Exiqon) using 5 µl Lipofectamine (Thermo Fisher Scientific) in 1.5 ml DMEM (Biochrom; without FCS) following manufacturer´s instructions. After 24 h the medium was changed and cells were kept on Claycomb medium with supplements for further 48 h.

**Western Blot**

For Western Blot analysis HL-1 cells were washed with PBS and lysed in RIPA buffer and sonicated (UP100H; Hielscher, Teltow, Germany). Cell lysates were matched for protein content. After separation the proteins were transferred to a PVDF membrane (Thermo Fisher Scientific, Waltham, USA) for p27 or a nitrocellulose membrane (GE Healthcare, Buckinghamshire, UK) for GIRK1, GIRK4, and HSP90. The membrane was incubated with primary antibodies (p27: 1:500, ab137736, Abcam, Cambridge, UK; GIRK1: 1:1000, ab129182, Abcam, Cambridge, UK; GIRK4: 1:750, ab113699, Abcam, Cambridge, UK; HSP90: 1:1000, 4874, Cell Signaling Technology, Danvers, USA) at 4°C overnight. The bound primary antibody was visualized using horseradish peroxidase-conjugated secondary IgG (anti-rabbit, 1:10,000/1:20,000 for GIRK1/4 & HSP90, Rockland, Limerick, USA) and the ECL system (Amersham, Freiburg, Germany). Densitometry analysis was performed with Quantity One software (Biorad, Munich, Germany).

**Electrophysiology**

Single HL-1 cells were plated for 24 hours on gelatin/fibronectin-coated 35 mm Petri dishes in 2 ml of Claycomb medium. The cells were then serum starved for one day before miR‑221/-222 (10 µg/ml) was added to the medium. 48h after drug application current recordings were performed in the whole-cell configuration of the patch-clamp technique using an Axopatch 200A patch-clamp amplifier (Axon Instruments, Inc., Burlingame, CA, USA). Patch pipettes were fabricated from thick wall (2 mm OD) borosilicate glass capillaries (Hilgenberg, Malsfeld, Germany) and filled with an internal solution of the following composition (in mmol/L): 130 CsCl, 20 TEACl, 10 EGTA, 5 Na_2_ATP, 6 MgCl_2_, 10 HEPES (pH was adjusted with CsOH to 7.2). Electrical resistances of the fire polished electrodes were 3‑4 MΩ when filled with internal solution. L-type Ca^2+^ currents were recorded in a Na^+^- free and K^+^-free bath solution containing (in mmol/L): 150 Tris-Cl, 10 CaCl_2_, 10 glucose, 10 HEPES (pH was adjusted with Tris-OH to 7.4). Current signals were sampled at 16 to 40 kHz and low pass filtered at 5 kHz with a four-pole Bessel filter and stored for off-line analysis (ISO2, MFK, Germany). Series resistance was partially compensated (>70%). By integrating the capacitive current at the end of a 10 ms long voltage step (-80 mV to -70 mV) the input capacitance of the cells was obtained. This value was normalized to the peak amplitude of the inward current to obtain the current density (pA/pF) in order to compensate for differences in cell size. All experiments were carried out at room temperature (20-24°C). HL-1 cells express both T-type and L-type Ca^2+^ currents [8]. Since in this study we sought to investigate the effect of miR-221/-222 solely on the activity of L-type Ca^2+^ channels, we used a voltage clamp protocol to separate the two currents from each other. T-type Ca^2+^-channels - recorded with 10 mmol/L Ca^2+^ as the charge carrier- were inactivated by using a holding potential of -35 mV without affecting the availability of L-type Ca^2+^channels. In order to obtain the maximal peak inward current (peakI_Ca,L_), HL-1 cells were depolarized every 8 s for 100 ms from a holding potential of -35 mV to various test potentials (+15 mV to 30 mV in 5 mV increments).

**Incubation with angiotensin II**

HEK-293 cells were transfected on 10mm Petri dishes with 2 µg pAT1R (human angiotensin II receptor type 1) (or empty vector control) for 24h. After transfection cells were splitted (to three 3.5mm Petri dishes each) and incubated with 100 nM angiotensin II (Sigma) or control for further 48h. miR expression was analyzed by qRT-PCR using Taqman microRNA Assays as described above.

**Statistical analysis**

Data are presented as mean ± standard error of mean. Anova followed by post hoc testing, Student´s T-Test or Mann-Whitney Rank Sum Test were used as applicable according to pre-test data analysis by SigmaPlot 12.5. A p-value <0.05 was considered significant. Biometrical planning was performed with α = 0.05 and β = 0.8, resulting in sample sizes between 5 and 15 samples/group depending on the experimental setting.

Reference List

1. Braun J, Misiak D, Busch B, Krohn K, Hüttelmaier S (2014) Rapid identification of regulatory microRNAs by miTRAP (miRNA trapping by RNA in vitro affinity purification). Nucleic Acids Res 42:e66

2. Eden E, Navon R, Steinfeld I, Lipson D, Yakhini Z (2009) GOrilla: a tool for discovery and visualization of enriched GO terms in ranked gene lists. BMC Bioinformatics 10:48

3. Kamkin A, Kiseleva I, Isenberg G (2003) Ion selectivity of stretch-activated cation currents in mouse ventricular myocytes. Pflugers Arch 446:220-231

4. Reimand J, Arak T, Adler P, Kolberg L, Reisberg S, Peterson H, Vilo J (2016) g:Profiler-a web server for functional interpretation of gene lists (2016 update). (2016 update). Nucleic Acids Res 44:W83-W89

5. Schreier B, Rabe S, Schneider B, Bretschneider M, Rupp S, Ruhs S, Neumann J, Rueckschloss U, Sibilia M, Gotthardt M, Grossmann C, Gekle M (2013) Loss of Epidermal Growth Factor Receptor in Vascular Smooth Muscle Cells and Cardiomyocytes Causes Arterial Hypotension and Cardiac Hypertrophy. Hypertension 61:333-340

6. Schreier B, Rabe S, Schneider B, Ruhs S, Grossmann C, Hauptmann S, Blessing M, Neumann J, Gekle M (2011) Aldosterone/NaCl-induced renal and cardiac fibrosis is modulated by TGF-beta responsiveness of T cells. Hypertens Res 34:623-629

7. Schreier B, Rabe S, Winter S, Ruhs S, Mildenberger S, Schneider B, Sibilia M, Gotthardt M, Kempe S, Mader K, Grossmann C, Gekle M (2014) Moderate inappropriately high aldosterone/NaCl constellation in mice: cardiovascular effects and the role of cardiovascular epidermal growth factor receptor. Sci Rep 4:7430

8. Xia M, Salata JJ, Figueroa DJ, Lawlor AM, Liang HA, Liu Y, Connolly TM (2004) Functional expression of L- and T-type Ca2+ channels in murine HL-1 cells. J Mol Cell Cardiol 36:111-119

9. Zambelli F, Pesole G, Pavesi G (2009) Pscan: finding over-represented transcription factor binding site motifs in sequences from co-regulated or co-expressed genes. Nucleic Acids Res 37:W247-W252
