# Supplementary material for: miR-221 and -222 target CACNA1C and KCNJ5 leading to altered cardiac ion channel expression and current density
Source: Cell Mol Life Sci. 2019 Jul 16;77(5):903–18. doi: 10.1007/s00018-019-03217-y (PMC7058603; doi:10.1007/s00018-019-03217-y)
Supplement: Supplementary file 2 — Supplementary material 2 (PDF 473 kb) [file 18_2019_3217_MOESM2_ESM.pdf]

NGS miR (WT = 1,3,5,7,9,11; KO = 2,4,6,8,10,12)

	BS_1	BS_3	BS_5	BS_7	BS_9	BS_11	BS_2	BS_4	BS_6	BS_8	BS_10	BS_12
mmu-miR-221-3p	542	663	538	602	533	567	1005	1295	1120	1350	1191	1338
mmu-miR-222-3p	106	124	89	76	90	102	204	234	219	225	236	284
mmu-miR-6898-3p	90	104	117	82	136	112	41	16	15	21	27	30
mmu-miR-27b-3p	22801	19788	19360	19157	16990	15230	35758	27502	34480	31955	31829	29562
mmu-miR-208b-3p	163	244	94	120	139	150	4879	2646	5873	2996	3741	3673
mmu-miR-1941-5p	143	190	192	189	269	197	76	16	62	38	73	62
mmu-miR-27b-5p	25	18	20	25	31	25	39	38	39	41	52	46
mmu-miR-201-5p	2	5	3	0	7	3	12	10	14	14	9	12
mmu-miR-30c-1-3p	98	88	99	101	93	105	76	82	80	71	78	84
mmu-let-7d-3p	415	355	425	376	358	420	322	284	233	263	288	305
mmu-miR-490-3p	305	311	303	363	299	315	240	242	287	242	245	239
mmu-miR-23b-3p	532	452	405	536	510	437	867	556	718	859	721	735
mmu-miR-218-5p	35	58	43	47	31	49	19	20	30	17	16	27
mmu-miR-455-3p	14	9	11	18	6	12	31	24	35	22	30	17
mmu-miR-214-5p	6	3	4	2	6	1	12	8	10	12	8	7
mmu-miR-195a-5p	326	317	242	311	290	237	506	360	564	480	418	379
mmu-miR-3084-5p	22	22	34	22	55	46	10	4	8	12	10	4
mmu-miR-31-3p	0	0	0	0	0	0	1	1	1	1	3	1
mmu-miR-8094	1	1	1	1	0	1	0	0	0	0	0	0
mmu-miR-9-5p	54	66	67	100	77	102	36	40	49	30	36	59
mmu-miR-7669-5p	1	1	1	0	1	1	1	0	0	0	0	0
mmu-miR-547-3p	6	5	4	8	0	3	19	8	13	12	13	8
mmu-miR-7688-5p	5	1	5	7	1	4	5	8	13	16	15	12
mmu-miR-690	25	32	48	26	61	69	8	2	17	14	21	18
mmu-miR-6971-3p	1	1	2	2	3	3	1	1	0	1	0	1
mmu-miR-199b-3p	1326	1611	1415	1696	1376	1545	1759	2405	2858	3752	2248	2265
mmu-miR-3074-5p	81	94	77	70	83	88	131	85	125	106	140	164
mmu-miR-10b-5p	3785	4460	3747	3648	4060	4650	4644	5077	8489	6936	6963	9884
mmu-miR-181a-2-3p	132	142	183	100	166	162	77	111	97	45	98	116
mmu-miR-483-3p	1	1	0	1	1	1	0	1	0	0	0	0
mmu-miR-3073b-3p	2	3	2	2	3	0	1	1	0	1	0	0
mmu-miR-199a-3p	1430	1557	1370	1694	1481	1405	1850	2178	3139	3915	2431	2071
mmu-miR-214-3p	49	29	36	35	15	22	62	35	66	68	59	41
mmu-miR-199a-5p	229	157	155	159	118	97	378	205	268	258	242	202
mmu-miR-6346	1	0	3	2	3	1	0	1	0	0	1	0
mmu-miR-219b-3p	0	1	0	0	1	0	2	2	0	2	2	2
mmu-miR-671-3p	27	27	35	33	34	18	31	54	52	50	33	47
mmu-miR-5123	0	1	0	0	0	0	1	1	1	1	1	0
mmu-miR-30e-3p	3647	4439	4446	3575	4914	5690	2590	3967	2683	1980	3101	3818
mmu-miR-509-3p	1	1	1	1	1	3	0	1	1	0	0	0
mmu-miR-1247-5p	4	2	2	3	3	1	6	5	7	14	9	4
mmu-miR-30d-3p	65	74	60	65	50	69	32	57	60	38	49	41
mmu-miR-3066-3p	35	56	80	53	174	130	16	6	17	18	33	28
mmu-miR-328-3p	482	556	478	462	432	458	440	460	367	355	377	417
mmu-miR-7225-5p	4	1	5	2	3	7	1	2	1	0	0	0
mmu-miR-30e-5p	15274	16493	15928	16835	12319	11332	12113	13279	12300	11165	10111	9471
mmu-miR-135b-3p	12	13	19	20	30	24	7	12	11	11	12	14
mmu-miR-3091-3p	0	0	0	0	0	0	1	1	0	1	1	0
mmu-miR-6914-3p	4	1	3	1	0	1	0	0	0	0	0	0
mmu-miR-700-3p	2	3	3	4	3	1	8	4	6	8	6	2
mmu-miR-204-5p	173	174	142	211	157	143	174	195	208	266	209	221
mmu-miR-322-3p	96	83	82	101	86	65	111	99	93	119	106	94
mmu-miR-3065-3p	1	1	3	2	4	3	1	2	1	1	0	1
mmu-miR-188-3p	0	0	0	0	0	0	0	1	1	1	1	0
mmu-miR-425-5p	131	164	123	177	160	178	199	172	221	250	172	179
mmu-miR-3098-5p	1	1	2	0	1	3	0	0	1	0	0	0
mmu-miR-6939-5p	5	8	11	9	24	13	6	1	3	3	7	4
mmu-miR-351-5p	168	139	118	102	80	71	182	166	169	170	139	130
mmu-miR-28a-5p	243	268	238	286	231	274	256	329	399	414	300	284
mmu-miR-208b-5p	0	1	0	0	0	0	2	0	4	1	2	5
mmu-miR-1839-5p	151	189	170	205	185	222	169	269	236	332	232	271
mmu-miR-3569-5p	1	1	4	3	4	7	1	1	1	1	2	0
mmu-miR-107-3p	142	193	166	213	211	212	115	195	125	141	111	164
mmu-miR-193b-3p	32	19	17	24	13	12	37	30	34	26	31	22
mmu-miR-497-5p	72	67	71	63	59	59	121	79	118	130	65	69
mmu-miR-125b-1-3p	25	22	10	22	25	18	32	24	28	38	38	21
mmu-miR-301a-3p	66	81	84	76	64	74	68	100	105	128	82	101
mmu-miR-7578	2	1	2	2	1	0	0	1	0	1	0	1
mmu-miR-7214-5p	4	2	2	2	4	3	1	1	3	1	1	2
mmu-miR-7667-5p	36	16	26	21	34	37	14	9	14	20	25	22
mmu-miR-344d-1-5p	1	1	2	0	0	1	1	0	0	0	0	0
mmu-miR-669a-5p	2	3	1	4	3	4	2	1	3	1	1	0
mmu-miR-335-5p	98	79	59	75	49	37	141	88	129	150	67	80
mmu-miR-7046-3p	0	1	0	0	0	0	1	1	0	1	3	1
mmu-miR-128-1-5p	2	1	2	2	1	0	3	1	6	2	4	4
mmu-miR-148b-3p	1805	2260	1713	1773	2091	2756	2115	3264	2816	2082	3032	3616
mmu-miR-24-3p	2253	1902	1645	1878	1269	1152	3276	1979	2652	2354	2142	1802
mmu-miR-30d-5p	14830	16771	15289	16403	12051	11388	13101	12808	12542	11915	10668	10929
mmu-miR-708-3p	2	4	3	4	4	0	1	3	1	0	1	0
mmu-miR-1306-5p	1	2	3	2	4	3	2	1	3	0	1	0
mmu-miR-3962	0	2	3	2	4	3	2	1	0	1	1	0
mmu-miR-344d-3-5p	6	9	6	5	33	25	2	2	3	2	4	3
mmu-miR-320-5p	1	0	1	1	0	1	0	0	0	0	1	0
mmu-miR-6412	9	2	7	2	6	6	0	4	1	1	5	0
mmu-miR-6960-5p	7	6	9	15	4	16	5	6	4	6	7	2
mmu-miR-129-2-3p	4	1	2	2	1	0	2	2	4	2	4	5
mmu-miR-199b-5p	27	17	18	19	12	15	42	22	38	29	21	18
mmu-miR-7a-1-3p	9	7	9	4	7	7	13	13	10	8	12	7
mmu-miR-582-5p	1	1	1	2	6	3	4	3	4	10	4	7
mmu-miR-466g	1	2	2	2	0	0	0	1	0	0	0	0
mmu-miR-7062-5p	1	1	0	1	0	1	1	0	0	0	0	0
mmu-miR-7655-3p	2	1	1	0	1	1	0	1	0	1	0	0
mmu-miR-490-5p	4	3	2	2	0	1	2	1	0	1	1	0
mmu-miR-122-3p	1	3	3	2	1	1	2	2	6	4	3	3
mmu-miR-429-3p	1	1	1	0	1	0	1	0	0	0	0	0
mmu-miR-194-5p	65	72	71	79	52	66	65	63	48	62	53	46

NGS miR (WT = 1,3,5,7,9,11; KO = 2,4,6,8,10,12)

mmu-miR-6906-3p	1	1	0	1	0	0	0	0	0	0	0	0
mmu-miR-195a-3p	15	19	15	13	28	13	12	30	27	31	22	37
mmu-miR-7116-3p	5	1	3	1	3	4	1	1	0	0	3	2
mmu-miR-378a-5p	142	191	177	191	186	186	167	173	159	116	142	163
mmu-miR-6377	1	0	0	1	1	0	0	0	0	0	0	0
mmu-miR-29b-2-5p	2	4	3	2	6	10	2	1	1	2	3	2
mmu-miR-3102-3p	56	61	52	54	44	25	31	39	48	38	31	26
mmu-miR-335-3p	25	38	25	46	30	27	51	51	41	76	40	28
mmu-miR-668-5p	1	0	1	0	1	0	0	0	0	0	0	0
mmu-miR-1983	5	1	2	7	3	1	5	7	4	11	14	3
mmu-miR-1298-5p	0	0	1	1	0	1	0	0	0	0	0	0
mmu-miR-346-5p	0	0	1	1	0	1	0	0	0	0	0	0
mmu-miR-713	0	0	1	1	1	0	0	0	0	0	0	0
mmu-miR-7651-5p	0	0	1	1	1	0	0	0	0	0	0	0
mmu-miR-139-3p	14	25	25	20	10	18	14	13	11	17	11	12
mmu-miR-212-5p	2	3	7	8	1	4	13	3	14	19	8	4
mmu-miR-6923-3p	1	0	2	0	0	1	0	0	0	0	0	0
mmu-miR-34b-5p	4	4	7	3	0	3	3	7	10	10	6	4
mmu-miR-301a-5p	1	1	1	1	0	0	0	0	0	0	1	0
mmu-miR-7218-3p	1	0	5	4	4	1	2	1	0	0	1	1
mmu-miR-92b-3p	11	9	6	11	13	10	8	15	14	12	20	26
mmu-miR-6912-3p	0	0	0	0	0	0	1	0	0	1	1	0
mmu-miR-466f-3p	1	1	2	1	4	0	0	0	0	0	0	1
mmu-miR-219a-2-3p	1	1	1	0	0	0	0	0	0	0	0	0
mmu-miR-6973a-5p	1	1	0	1	0	0	0	0	0	0	0	0
mmu-miR-7a-2-3p	1	1	0	1	0	0	0	0	0	0	0	0
mmu-miR-7242-3p	0	0	0	0	0	0	0	1	0	0	1	1
mmu-miR-488-5p	0	1	0	0	0	0	1	1	0	0	1	1
mmu-miR-7092-5p	0	0	0	0	0	0	2	0	0	1	0	1
mmu-miR-5113	5	1	1	5	1	4	1	1	0	1	3	1
mmu-miR-21a-5p	2173	3701	2418	3372	2868	3689	2728	4727	5427	10081	3932	4684
mmu-miR-30c-5p	8171	7626	7277	7493	4926	4553	7371	4924	4969	4685	4294	3681
mmu-miR-672-5p	1	2	1	0	3	1	1	1	6	3	3	4
mmu-miR-296-5p	2	1	0	0	1	0	3	3	3	0	3	1
mmu-miR-6968-3p	0	1	1	1	3	1	0	1	0	1	1	0
mmu-miR-1906	1	1	0	0	0	1	0	0	0	0	0	0
mmu-miR-6928-3p	1	1	0	0	0	1	0	0	0	0	0	0
mmu-miR-7018-5p	1	1	0	0	0	1	0	0	0	0	0	0
mmu-miR-182-3p	0	1	0	1	1	0	0	0	0	0	0	0
mmu-miR-6925-3p	0	1	0	1	1	0	0	0	0	0	0	0
mmu-miR-7062-3p	0	1	0	1	1	0	0	0	0	0	0	0
mmu-miR-30a-3p	1724	1897	2175	1360	2112	2741	1323	1817	1311	808	1589	2013
mmu-miR-338-5p	2	5	3	1	1	1	1	2	0	2	1	0
mmu-miR-378a-3p	13169	16683	15749	16727	18829	21395	9937	18074	13839	11786	13649	15497
mmu-miR-1907	0	1	0	0	1	1	0	0	0	0	0	0
mmu-miR-7089-3p	0	0	0	0	0	0	0	1	1	0	0	1
mmu-miR-369-5p	0	1	1	0	0	1	0	0	0	0	0	0
mmu-miR-6934-3p	0	1	1	0	0	1	0	0	0	0	0	0
mmu-miR-669a-3-3p	0	1	1	0	1	0	0	0	0	0	0	0
mmu-miR-221-5p	16	16	23	10	18	9	41	14	25	11	36	28
mmu-miR-3470a	5	11	9	4	24	27	9	3	4	0	7	7
mmu-miR-15b-3p	4	8	3	4	1	6	9	9	4	7	8	4
mmu-miR-7222-5p	2	1	4	0	1	6	0	1	0	0	0	2
mmu-miR-7073-3p	1	2	0	1	3	3	0	0	0	2	1	0
mmu-miR-34a-3p	0	1	0	0	0	0	1	1	1	0	0	1
mmu-miR-666-5p	0	0	0	0	0	0	1	1	3	1	0	0
mmu-miR-664-5p	5	9	16	13	12	6	6	17	17	18	16	16
mmu-miR-1943-5p	22	22	16	18	28	31	15	20	13	16	17	23
mmu-let-7e-5p	1116	1063	980	1181	925	947	1134	1064	1190	1773	1236	1165
mmu-miR-411-5p	11	15	19	9	15	9	9	14	24	26	17	25
mmu-miR-1957b	0	1	0	1	3	1	1	0	0	0	0	0
mmu-miR-470-5p	1	1	0	0	0	0	0	1	1	4	1	1
mmu-miR-135a-5p	6	3	6	4	4	3	1	5	3	3	1	4
mmu-miR-152-3p	203	198	189	193	183	169	190	193	194	219	225	206
mmu-miR-878-3p	78	36	63	79	93	106	18	32	48	55	59	84
mmu-miR-341-3p	1	1	0	0	0	1	1	1	3	2	1	1
mmu-let-7f-1-3p	25	25	19	25	25	16	19	22	15	20	18	18
mmu-miR-760-3p	1	1	0	2	4	1	1	0	0	0	1	0
mmu-miR-133a-5p	421	429	464	445	310	215	354	347	323	286	232	179
mmu-miR-29c-5p	7	7	6	8	1	6	4	5	3	3	3	5
mmu-miR-1843b-5p	32	35	36	45	38	43	33	45	39	74	49	67
mmu-miR-7240-3p	4	3	3	5	0	0	0	1	0	0	2	1
mmu-miR-7216-5p	0	0	0	1	3	1	0	0	0	0	0	0
mmu-miR-6996-5p	0	0	0	0	0	0	1	1	1	0	0	0
mmu-miR-185-5p	163	166	154	202	140	124	150	156	125	126	136	110
mmu-miR-18a-3p	0	0	0	0	0	0	2	1	0	0	2	0
mmu-miR-6907-5p	4	4	5	4	9	7	1	1	1	3	5	7
mmu-miR-155-5p	60	81	74	76	50	47	62	57	51	59	43	30
mmu-miR-7075-5p	0	1	1	2	3	0	0	0	1	0	0	0
mmu-miR-3083-5p	0	1	2	2	3	3	2	1	0	0	1	1
mmu-miR-1a-1-5p	21	11	14	11	12	16	6	9	15	11	12	3
mmu-miR-1191b-3p	0	0	2	0	1	1	1	0	0	0	0	0
mmu-miR-3473d	1	3	1	1	3	1	1	1	1	0	1	1
mmu-miR-450a-2-3p	2	1	0	0	0	1	0	0	0	0	0	0
mmu-miR-5101	0	1	2	2	1	3	1	0	0	0	2	1
mmu-miR-6388	1	0	1	0	0	3	0	0	0	0	0	0
mmu-miR-99b-3p	6	8	5	9	18	12	9	10	17	13	18	15
mmu-miR-3087-3p	0	1	1	3	1	3	0	1	0	1	1	0
mmu-miR-98-5p	1313	1420	1456	1732	1230	1458	1319	1762	1797	2168	1514	1558
mmu-miR-5134-3p	1	1	0	0	1	3	1	1	0	0	0	0
mmu-miR-1843a-5p	147	136	139	136	158	168	110	219	194	178	161	201
mmu-miR-7669-3p	2	1	1	0	1	4	0	0	1	0	0	1
mmu-miR-466c-5p	1	0	1	1	3	0	0	1	0	0	1	0
mmu-miR-5107-5p	1	0	2	0	4	0	0	0	0	0	0	0
mmu-miR-7647-5p	0	1	0	1	1	1	0	0	0	1	0	0
mmu-miR-877-3p	2	3	3	1	0	1	1	0	0	0	1	2
mmu-miR-3620-5p	2	1	0	0	1	0	0	1	0	0	0	0

NGS miR (WT = 1,3,5,7,9,11; KO = 2,4,6,8,10,12)

mmu-miR-7091-3p	0	0	2	0	1	1	0	0	0	0	1	0
mmu-miR-6236	11	10	18	3	44	63	2	1	1	2	1	28
mmu-miR-667-3p	1	1	0	0	3	1	1	1	0	0	1	0
mmu-miR-664-3p	12	15	13	12	13	4	13	15	13	17	17	13
mmu-miR-500-3p	0	3	2	3	1	3	4	2	8	3	3	2
mmu-miR-3102-5p.2-5	2	1	3	1	0	4	1	0	0	2	1	0
mmu-miR-34c-5p	55	25	40	38	16	25	29	42	49	75	39	55
mmu-miR-3960	0	1	1	0	1	0	1	2	1	0	1	2
mmu-miR-744-5p	14	24	25	25	13	32	18	11	13	9	23	21
mmu-miR-3470b	9	13	24	15	33	29	9	9	15	12	19	14
mmu-miR-23a-3p	936	771	730	832	733	585	1261	685	968	988	892	773
mmu-miR-132-3p	31	32	39	33	37	43	56	52	22	43	44	52
mmu-miR-1191	2	1	0	3	1	3	2	3	4	2	2	3
mmu-miR-1947-5p	5	9	8	2	3	3	7	6	7	6	7	9
mmu-miR-3102-3p.2-3	4	1	0	1	1	4	2	1	0	0	1	0
mmu-miR-340-3p	21	17	16	15	6	6	11	7	8	7	7	13
mmu-miR-6944-3p	1	3	2	0	3	1	2	3	3	9	2	3
mmu-miR-6244	911	418	831	468	621	488	782	287	344	295	414	464
mmu-miR-6910-3p	2	3	0	1	1	4	0	0	0	0	3	1
mmu-miR-22-3p	50522	46689	47283	37930	43153	40145	45485	40704	39260	27585	40648	40435
mmu-miR-592-5p	1	1	2	1	1	1	2	1	0	0	0	1
mmu-miR-8103	4	3	1	2	4	7	1	3	0	2	2	2
mmu-miR-669p-5p	5	5	4	0	4	1	2	4	0	0	2	1
mmu-miR-694	0	0	0	1	1	3	0	1	0	0	0	0
mmu-miR-3964	0	0	1	1	0	1	1	0	0	0	0	0
mmu-miR-7068-3p	32	42	35	42	41	43	29	61	45	49	43	55
mmu-miR-872-3p	9	8	3	8	3	10	11	9	10	8	11	7
mmu-miR-23b-5p	0	1	1	1	0	0	1	0	0	0	0	0
mmu-miR-1964-3p	4	3	4	2	7	1	1	1	0	1	1	5
mmu-miR-6390	14	11	10	16	33	19	6	12	14	8	14	12
mmu-miR-1930-5p	1	0	1	2	1	0	1	1	0	0	0	0
mmu-miR-673-5p	1	1	0	0	0	3	1	3	0	2	4	2
mmu-miR-5129-5p	0	1	0	0	3	3	0	1	0	0	0	0
mmu-miR-6941-3p	0	0	1	0	1	1	0	0	0	0	1	0
mmu-miR-6948-5p	0	0	1	0	1	1	0	0	0	0	1	0
mmu-miR-1199-3p	0	0	1	0	0	0	1	1	0	0	1	1
mmu-miR-126a-5p	2593	2879	2127	2907	2898	2864	2937	2875	2826	3375	2698	3136
mmu-miR-129-1-3p	5	3	3	3	7	6	2	2	4	1	6	2
mmu-miR-6919-5p	4	3	2	1	0	0	0	2	0	0	0	0
mmu-miR-714	0	0	1	0	4	1	0	0	0	0	0	0
mmu-miR-7685-5p	0	1	1	1	0	3	0	0	1	0	0	0
mmu-miR-7649-5p	1	1	1	0	3	4	0	1	0	1	1	0
mmu-miR-184-5p	1	3	1	0	0	0	1	0	0	0	0	0
mmu-miR-29c-3p	406	358	326	329	228	203	371	211	263	218	207	160
mmu-miR-10a-3p	2	3	5	5	1	1	3	5	3	7	6	4
mmu-miR-345-3p	92	67	94	48	75	96	60	89	32	33	77	63
mmu-miR-6415	1	0	1	0	1	0	0	0	0	0	1	0
mmu-miR-208a-3p	1793	1381	1396	1255	1102	860	1543	1035	878	639	1121	839
mmu-miR-503-3p	7	6	3	5	7	9	5	6	3	6	4	4
mmu-miR-6516-5p	10	8	7	7	10	15	5	6	7	6	7	11
mmu-miR-192-3p	0	0	1	5	4	1	0	1	0	0	1	1
mmu-miR-184-3p	4	6	4	2	3	6	2	4	4	2	1	3
mmu-miR-5619-3p	0	0	0	1	0	0	0	0	0	1	14	11
mmu-miR-223-3p	29	52	30	46	46	46	32	28	31	47	31	33
mmu-miR-1957a	2	1	7	2	6	10	2	3	0	0	4	4
mmu-miR-129b-5p	0	0	0	0	1	1	0	0	0	0	0	0
mmu-miR-302b-5p	0	0	0	0	1	1	0	0	0	0	0	0
mmu-miR-375-5p	0	0	0	0	1	1	0	0	0	0	0	0
mmu-miR-5625-5p	0	0	0	0	1	1	0	0	0	0	0	0
mmu-miR-6949-5p	0	0	0	0	1	1	0	0	0	0	0	0
mmu-miR-6998-5p	0	0	0	0	1	1	0	0	0	0	0	0
mmu-miR-7221-3p	0	0	0	0	1	1	0	0	0	0	0	0
mmu-miR-7235-5p	0	0	0	0	1	1	0	0	0	0	0	0
mmu-miR-882	0	0	0	0	1	1	0	0	0	0	0	0
mmu-miR-409-5p	0	0	0	0	0	0	0	1	0	1	0	0
mmu-miR-471-5p	0	0	0	0	0	0	0	0	1	0	1	0
mmu-miR-7080-5p	1	0	2	2	6	10	1	0	0	0	1	3
mmu-miR-128-2-5p	0	0	0	0	0	0	1	1	0	0	0	0
mmu-miR-6378	0	2	0	0	1	3	0	1	0	0	1	0
mmu-miR-7243-3p	0	0	1	1	0	0	0	0	0	0	0	0
mmu-miR-8105	0	0	1	1	0	0	0	0	0	0	0	0
mmu-miR-7118-5p	0	0	0	0	0	0	1	0	1	0	0	0
mmu-miR-3081-5p	1	0	0	1	0	0	0	0	0	0	0	0
mmu-miR-6384	1	0	0	1	0	0	0	0	0	0	0	0
mmu-miR-6921-5p	1	0	0	1	0	0	0	0	0	0	0	0
mmu-miR-7089-5p	1	0	0	1	0	0	0	0	0	0	0	0
mmu-miR-19b-3p	44	37	33	49	40	43	47	37	34	33	23	34
mmu-miR-3076-3p	0	0	0	0	0	0	1	0	0	0	0	1
mmu-miR-6360	0	1	0	1	0	0	0	0	0	0	0	0
mmu-miR-7116-5p	0	1	0	0	1	0	0	0	0	0	0	0
mmu-miR-141-5p	0	0	0	0	0	0	1	0	0	0	1	0
mmu-miR-465c-5p	0	0	0	0	0	0	1	0	0	0	1	0
mmu-miR-6937-3p	0	0	0	0	0	0	1	0	0	0	1	0
mmu-miR-6938-3p	0	0	0	0	0	0	1	0	0	0	1	0
mmu-miR-759	0	0	0	0	0	0	1	0	0	0	1	0
mmu-miR-1902	1	0	0	0	0	1	0	0	0	0	0	0
mmu-miR-1930-3p	1	0	0	0	0	1	0	0	0	0	0	0
mmu-miR-3106-5p	1	0	0	0	0	1	0	0	0	0	0	0
mmu-miR-465d-3p	1	0	0	0	0	1	0	0	0	0	0	0
mmu-miR-3087-5p	1	0	0	0	1	0	0	0	0	0	0	0
mmu-miR-7679-3p	0	3	1	0	4	4	1	0	0	1	1	1
mmu-miR-3547-5p	2	0	2	0	4	3	1	1	0	1	0	2
mmu-miR-6352	1	0	1	0	0	0	0	0	0	0	0	0
mmu-miR-6944-5p	1	0	1	0	0	0	0	0	0	0	0	0
mmu-miR-7069-5p	1	0	1	0	0	0	0	0	0	0	0	0
mmu-miR-344-5p	0	0	0	0	0	0	0	1	0	0	1	0
mmu-miR-6930-3p	0	0	0	0	0	0	0	1	0	0	1	0

NGS miR (WT = 1,3,5,7,9,11; KO = 2,4,6,8,10,12)

mmu-miR-7078-3p	1	1	1	2	7	3	2	2	0	1	1	0
mmu-miR-6359	0	1	1	0	0	0	0	0	0	0	0	0
mmu-miR-5100	1	0	0	0	0	1	1	1	0	2	1	3
mmu-miR-6901-3p	0	0	2	0	1	0	0	0	0	0	0	0
mmu-miR-7221-5p	0	0	2	0	0	1	0	0	0	0	0	0
mmu-miR-216c-3p	0	0	0	1	0	1	0	0	0	0	0	0
mmu-miR-7210-5p	0	0	0	1	0	1	0	0	0	0	0	0
mmu-miR-881-3p	0	0	0	1	0	1	0	0	0	0	0	0
mmu-miR-1970	0	0	0	1	1	0	0	0	0	0	0	0
mmu-miR-6345	0	0	0	1	1	0	0	0	0	0	0	0
mmu-miR-6358	0	0	0	1	1	0	0	0	0	0	0	0
mmu-miR-7070-5p	0	0	0	1	1	0	0	0	0	0	0	0
mmu-miR-7b-3p	0	0	0	1	1	0	0	0	0	0	0	0
mmu-miR-6974-3p	0	0	0	2	3	0	0	0	0	0	0	0
mmu-miR-6367	1	1	0	0	1	1	0	0	1	0	0	0
mmu-miR-340-5p	146	232	188	222	216	240	144	286	240	266	274	274
mmu-miR-7092-3p	0	2	1	0	4	4	1	1	0	0	0	2
mmu-miR-6913-3p	0	2	2	1	3	0	0	1	0	0	1	1
mmu-miR-494-3p	5	0	0	0	15	25	1	0	0	0	1	3
mmu-miR-181c-5p	1379	1764	1544	1703	1887	1816	1387	2144	1949	2168	1743	2030
mmu-miR-874-3p	22	23	14	24	24	9	22	16	15	8	14	10
mmu-miR-126b-3p	2552	2942	2221	3123	2663	2883	2939	2969	2754	3337	2732	3155
mmu-miR-7093-5p	2	0	0	1	0	3	0	1	0	0	1	0
mmu-miR-6373	0	0	0	0	0	0	0	0	0	0	1	1
mmu-miR-7027-5p	0	0	0	0	0	0	0	0	0	0	1	1
mmu-miR-7029-5p	0	0	0	0	0	0	0	0	0	0	1	1
mmu-miR-7064-3p	0	0	0	0	0	0	0	0	0	0	1	1
mmu-miR-7665-3p	0	0	0	0	0	0	0	0	0	0	1	1
mmu-miR-5124a	0	1	0	0	4	1	0	0	0	0	0	0
mmu-miR-6902-5p	0	0	1	0	0	1	0	0	0	0	0	0
mmu-miR-6931-3p	0	0	1	0	0	1	0	0	0	0	0	0
mmu-miR-6952-5p	0	0	1	0	0	1	0	0	0	0	0	0
mmu-miR-7660-5p	0	0	1	0	0	1	0	0	0	0	0	0
mmu-miR-7210-3p	0	0	0	0	3	4	0	0	0	0	0	0
mmu-miR-135b-5p	1	0	1	1	3	0	0	0	0	0	1	1
mmu-miR-5127	0	0	1	0	1	0	0	0	0	0	0	0
mmu-miR-5129-3p	0	0	1	0	1	0	0	0	0	0	0	0
mmu-miR-6988-5p	0	0	1	0	1	0	0	0	0	0	0	0
mmu-miR-7038-5p	0	0	1	0	1	0	0	0	0	0	0	0
mmu-miR-8097	0	0	1	0	1	0	0	0	0	0	0	0
mmu-miR-1963	0	0	0	0	0	0	0	0	0	1	1	0
mmu-miR-466m-3p	0	0	0	0	0	0	0	0	0	1	1	0
mmu-miR-5626-3p	0	0	0	0	0	0	0	0	0	1	1	0
mmu-miR-712-5p	0	0	0	0	0	0	0	0	0	1	1	0
mmu-miR-211-3p	0	0	0	2	1	1	0	0	0	1	0	0
mmu-miR-7035-5p	0	1	1	0	0	0	0	0	0	0	0	0
mmu-miR-7046-5p	0	1	1	0	0	0	0	0	0	0	0	0
mmu-miR-7222-3p	0	1	1	0	0	0	0	0	0	0	0	0
mmu-miR-6909-3p	0	1	0	1	0	1	1	0	0	0	0	0
mmu-miR-361-3p	40	63	72	66	69	49	57	78	77	75	61	68
mmu-miR-29a-5p	10	7	7	7	10	7	7	6	6	3	7	9
mmu-miR-7074-3p	0	2	3	0	1	3	0	0	0	1	0	2
mmu-miR-361-5p	93	118	67	73	86	74	120	81	107	105	103	85
mmu-miR-5098	0	0	1	2	3	1	0	1	0	1	0	1
mmu-miR-706	5	5	4	2	21	27	1	1	3	1	8	9
mmu-miR-6896-3p	0	1	1	2	0	0	1	0	1	1	3	4
mmu-miR-6973b-5p	0	1	0	1	0	0	0	0	0	0	0	0
mmu-miR-7675-5p	0	1	0	1	0	0	0	0	0	0	0	0
mmu-miR-497-3p	0	0	0	0	0	0	1	0	0	0	0	1
mmu-miR-6546-5p	0	0	0	0	0	0	1	0	0	0	0	1
mmu-miR-101a-5p	5	8	13	13	9	6	7	5	7	3	10	4
mmu-miR-292-3p	0	0	0	0	0	0	1	0	0	1	0	0
mmu-miR-466e-3p	0	0	0	0	0	0	1	0	0	1	0	0
mmu-miR-5135	0	0	0	0	0	0	1	0	0	1	0	0
mmu-miR-499-5p	982	849	790	1021	596	499	1198	955	935	951	914	724
mmu-miR-5623-5p	1	0	1	0	12	6	0	0	0	0	1	1
mmu-miR-27a-3p	1293	1120	1082	1262	983	773	1605	1097	1258	1357	1153	1063
mmu-miR-700-5p	5	1	1	4	0	0	2	2	3	9	4	5
mmu-miR-1966-3p	0	0	1	0	1	3	1	2	1	4	1	2
mmu-miR-6985-5p	0	1	0	0	1	1	0	0	0	0	1	0
mmu-miR-7022-3p	0	1	0	0	1	1	0	0	0	0	1	0
mmu-miR-30b-5p	1667	1716	1575	1658	962	931	1619	1058	1242	1173	928	783
mmu-miR-541-5p	31	42	32	43	41	41	35	43	45	90	45	47
mmu-miR-6541	0	0	0	0	0	0	1	1	0	0	0	0
mmu-miR-501-5p	0	0	0	0	0	0	0	1	0	0	0	1
mmu-miR-7231-3p	0	0	0	0	0	0	0	1	0	0	0	1
mmu-miR-7091-5p	0	0	0	0	0	0	0	1	0	0	2	0
mmu-miR-504-5p	6	9	8	4	3	4	8	5	1	3	2	2
mmu-miR-6516-3p	0	0	0	0	0	0	0	1	0	2	0	0
mmu-miR-465b-5p	0	0	0	0	0	0	0	1	0	1	0	0
mmu-miR-7678-3p	0	0	0	0	0	0	0	1	0	1	0	0
mmu-miR-6935-5p	0	2	2	0	0	3	0	0	0	0	1	1
mmu-let-7g-3p	2	4	3	3	1	3	5	3	6	4	2	2
mmu-miR-675-5p	0	0	0	0	0	0	0	0	0	1	0	2
mmu-miR-6937-5p	19	11	36	9	151	149	3	4	0	1	5	86
mmu-miR-1897-5p	1	1	0	0	0	0	0	0	0	0	0	0
mmu-miR-7033-3p	1	1	0	0	0	0	0	0	0	0	0	0
mmu-miR-7650-3p	1	1	0	0	0	0	0	0	0	0	0	0
mmu-miR-7661-3p	0	1	1	2	0	0	0	1	0	0	0	0
mmu-miR-211-5p	0	1	2	2	0	0	1	0	1	3	1	3
mmu-miR-669o-5p	1	4	1	1	0	4	0	1	3	0	1	0
mmu-miR-7087-3p	0	0	0	0	1	3	0	0	0	0	0	0
mmu-miR-6237	26	20	26	11	33	43	12	9	11	3	12	45
mmu-let-7b-5p	1109	1049	1076	1081	760	762	1088	1123	1251	1344	983	890
mmu-miR-302b-3p	16	11	9	4	13	12	16	5	1	2	7	10
mmu-miR-31-5p	90	79	71	73	55	60	92	86	62	86	87	75
mmu-miR-7015-3p	1	3	2	3	3	6	2	2	4	1	1	1

NGS miR (WT = 1,3,5,7,9,11; KO = 2,4,6,8,10,12)

mmu-miR-3064-5p	0	1	0	0	1	3	0	0	0	0	1	0
mmu-miR-7646-5p	0	0	1	0	6	7	1	1	0	0	0	1
mmu-miR-186-3p	0	1	0	0	0	0	0	1	0	0	1	3
mmu-miR-93-3p	0	1	0	0	0	0	3	0	0	3	1	0
mmu-miR-5126	4	2	4	2	6	7	2	1	0	0	7	3
mmu-miR-3063-5p	0	0	1	2	0	0	0	0	0	0	0	0
mmu-miR-6905-5p	0	0	0	0	0	0	1	0	1	0	0	0
mmu-miR-212-3p	9	5	12	12	4	10	16	12	8	22	9	8
mmu-miR-297b-3p	0	1	0	0	0	1	0	0	0	0	0	0
mmu-miR-6910-5p	0	1	0	0	0	1	0	0	0	0	0	0
mmu-miR-7040-5p	0	1	0	0	0	1	0	0	0	0	0	0
mmu-miR-7648-5p	0	1	0	0	0	1	0	0	0	0	0	0
mmu-miR-465c-3p	0	1	0	0	1	0	0	0	0	0	0	0
mmu-miR-6371	0	1	0	0	1	0	0	0	0	0	0	0
mmu-miR-691	0	1	0	0	1	0	0	0	0	0	0	0
mmu-miR-6942-3p	0	1	0	0	1	0	0	0	0	0	0	0
mmu-miR-7663-3p	0	1	0	0	1	0	0	0	0	0	0	0
mmu-miR-463-5p	1	0	0	0	0	3	0	0	0	0	0	0
mmu-miR-339-5p	29	29	23	21	24	19	24	26	20	16	18	21
mmu-miR-7232-5p	1	1	1	2	7	3	0	1	0	0	3	2
mmu-miR-1946b	0	1	0	1	3	0	1	0	0	0	0	0
mmu-miR-7034-3p	0	0	1	0	1	3	0	0	0	0	0	1
mmu-miR-7653-5p	1	1	0	1	3	0	0	0	0	0	1	1
mmu-miR-6540-5p	1	1	0	1	1	0	1	0	0	1	0	0
mmu-miR-3967	0	0	1	0	1	0	1	1	0	3	1	1
mmu-miR-196a-2-3p	0	1	1	0	0	1	0	0	0	0	1	0
mmu-miR-322-5p	847	872	674	649	749	622	995	810	797	681	868	769
mmu-miR-6954-3p	2	0	0	0	0	3	0	1	0	0	0	0
mmu-miR-126b-5p	209	183	187	212	124	140	196	202	223	276	196	149
mmu-miR-194-1-3p	0	2	3	0	1	3	0	0	1	1	1	1
mmu-miR-467h	0	1	1	2	1	0	1	2	4	1	1	3
mmu-miR-669d-5p	0	3	0	2	1	0	1	2	3	2	1	2
mmu-miR-3075-3p	0	1	4	0	1	3	1	0	0	0	0	2
mmu-miR-182-5p	10	10	6	7	13	27	11	14	13	12	33	29
mmu-miR-3061-5p	1	0	0	1	3	1	2	2	4	0	1	5
mmu-miR-6981-5p	1	0	1	1	1	6	0	0	3	0	0	0
mmu-miR-7648-3p	2	0	1	0	1	0	1	0	0	0	1	0
mmu-miR-1981-3p	6	1	3	2	4	1	3	4	1	0	1	1
mmu-miR-3095-5p	0	0	1	0	0	3	0	0	0	0	0	0
mmu-miR-547-5p	0	0	1	0	0	3	0	0	0	0	0	0
mmu-miR-6932-5p	0	0	1	0	0	3	0	0	0	0	0	0
mmu-miR-26b-3p	9	7	5	9	10	4	7	7	11	11	7	12
mmu-miR-331-3p	7	4	5	11	4	6	11	9	13	7	5	7
mmu-miR-1948-5p	0	0	0	1	0	0	2	0	0	1	0	1
mmu-miR-181a-5p	8044	8857	8083	8575	10108	10942	7788	10835	9428	10676	9911	12257
mmu-miR-6925-5p	0	1	2	0	0	0	0	0	0	0	0	0
mmu-miR-6948-3p	4	4	6	4	0	0	1	1	1	4	0	0
mmu-miR-25-5p	1	1	2	1	1	0	1	1	0	1	1	0
mmu-miR-7054-5p	1	1	2	0	0	1	1	1	0	0	0	1
mmu-miR-5615-3p	1	1	0	1	1	1	0	1	0	0	1	1
mmu-miR-21b	0	0	2	0	1	1	0	0	1	0	0	0
mmu-miR-8114	0	6	0	4	0	0	6	3	1	7	4	1
mmu-miR-151-3p	1818	2151	1846	2063	2466	2394	1806	2610	2161	2399	2346	2975
mmu-miR-1962	0	0	1	3	0	0	0	0	0	0	0	0
mmu-miR-135a-2-3p	1	3	2	1	3	1	1	1	1	1	1	2
mmu-miR-1194	4	1	0	4	3	7	0	2	3	2	0	2
mmu-miR-7042-3p	0	0	2	0	0	3	1	0	0	0	0	0
mmu-miR-1843b-3p	6	5	0	2	3	3	3	3	7	8	4	3
mmu-miR-8112	0	1	0	0	0	0	1	1	0	0	1	1
mmu-miR-7235-3p	1	0	0	2	3	4	1	1	0	0	3	0
mmu-miR-467c-5p	1	0	0	0	1	0	1	1	3	1	1	0
mmu-miR-764-5p	0	0	0	0	0	0	1	0	0	0	0	2
mmu-miR-200a-3p	4	6	5	11	4	4	3	4	0	3	8	4
mmu-miR-381-3p	9	10	12	3	7	16	6	12	13	12	17	16
mmu-let-7d-5p	3483	3032	3430	3432	2227	2170	3217	2278	2494	2881	2545	1924
mmu-miR-582-3p	4	1	3	0	13	4	9	3	3	2	18	18
mmu-miR-6238	72	79	172	129	928	1137	11	15	30	31	56	646
mmu-miR-330-5p	21	17	12	13	9	9	11	16	6	7	15	5
mmu-miR-143-5p	16	21	17	13	19	15	18	20	18	13	26	22
mmu-miR-693-3p	0	0	0	0	0	0	0	1	0	0	0	2
mmu-miR-133a-3p	114193	111868	98979	112065	78872	59659	117328	78395	81657	71506	71100	63449
mmu-miR-434-5p	10	11	18	11	12	12	12	6	11	10	13	10
mmu-miR-380-3p	1	4	1	0	1	0	1	0	1	0	1	0
mmu-miR-3970	4	6	9	7	13	3	6	7	3	6	39	33
mmu-miR-93-5p	419	476	397	499	432	539	493	518	500	510	472	454
mmu-miR-872-5p	88	84	78	92	55	71	76	74	65	65	62	78
mmu-miR-8110	0	0	1	0	0	0	2	0	0	1	1	0
mmu-miR-7003-5p	1	2	1	0	0	0	1	0	0	0	1	0
mmu-miR-337-3p	0	1	1	1	1	3	1	0	0	1	0	1
mmu-miR-6366	2	1	0	0	0	0	0	0	0	0	0	0
mmu-miR-378b	12	16	15	16	25	28	13	16	15	9	15	21
mmu-let-7b-3p	121	121	82	122	136	115	114	111	96	139	206	176
mmu-miR-7049-5p	0	0	2	0	3	0	0	0	0	0	1	0
mmu-miR-1982-3p	0	0	1	0	0	0	2	0	0	0	1	1
mmu-miR-365-1-5p	14	8	7	4	95	10	11	4	3	6	0	4
mmu-miR-6338	2	0	0	2	0	1	1	0	0	0	1	0
mmu-miR-7030-3p	0	0	2	0	1	0	0	1	0	0	0	0
mmu-miR-8118	0	0	2	0	1	0	0	1	0	0	0	0
mmu-miR-6991-5p	0	0	1	0	0	4	0	0	0	0	0	0
mmu-miR-653-3p	0	0	0	0	0	0	1	0	3	0	0	0
mmu-miR-6991-3p	0	1	1	0	0	0	1	0	1	0	3	1
mmu-miR-362-3p	5	3	6	5	4	6	6	5	1	2	4	5
mmu-miR-216b-5p	0	1	0	0	3	0	0	0	0	0	0	0
mmu-miR-6916-5p	0	1	0	0	3	0	0	0	0	0	0	0
mmu-miR-6940-5p	0	1	0	0	3	0	0	0	0	0	0	0
mmu-miR-7094-3p	0	0	0	0	3	3	0	0	0	0	0	1
mmu-miR-146a-5p	1389	1457	1214	1408	819	669	1622	1325	1691	1720	1129	904

NGS miR (WT = 1,3,5,7,9,11; KO = 2,4,6,8,10,12)

mmu-miR-3472	0	0	1	0	6	1	1	0	0	0	0	0
mmu-miR-1291	6	2	8	2	28	10	6	3	4	8	3	3
mmu-miR-7081-5p	1	1	1	0	0	0	0	0	0	1	0	0
mmu-miR-465d-5p	0	0	0	0	0	0	0	1	3	0	0	0
mmu-miR-7118-3p	0	0	0	0	3	1	1	0	0	0	0	0
mmu-miR-7039-3p	0	0	3	0	0	1	1	0	0	0	0	0
mmu-miR-574-5p	14	19	13	18	28	15	11	14	17	20	8	14
mmu-miR-3113-3p	0	0	2	1	1	0	0	0	1	0	0	0
mmu-miR-183-5p	1	3	0	1	3	7	1	3	0	0	2	0
mmu-miR-3963	56	10	17	3	38	62	17	13	6	13	32	26
mmu-miR-743b-3p	0	1	3	4	6	1	1	0	0	2	2	3
mmu-miR-7067-5p	0	0	0	0	1	1	0	1	0	0	0	0
mmu-miR-496a-3p	0	1	0	0	3	0	0	1	0	0	0	0
mmu-miR-1942	2	1	1	3	1	3	2	1	7	1	5	3
mmu-miR-10b-3p	1	1	0	0	1	0	4	1	1	1	1	0
mmu-miR-486-5p	47614	51448	49090	57156	56969	62190	47486	66626	56677	55332	56373	73579
mmu-miR-1955-3p	0	1	0	1	0	3	1	1	0	0	0	0
mmu-miR-6361	0	0	0	0	1	3	0	0	0	0	1	0
mmu-miR-468-3p	0	0	0	0	3	1	0	0	0	0	1	0
mmu-miR-6914-5p	0	0	0	0	1	1	1	0	0	0	0	0
mmu-miR-7220-5p	0	0	2	0	1	7	0	0	0	0	0	2
mmu-miR-134-5p	1	6	9	7	1	6	1	5	1	6	2	4
mmu-miR-770-5p	0	1	1	1	3	1	1	0	1	0	1	1
mmu-miR-433-5p	0	0	3	0	3	4	1	0	0	0	1	2
mmu-miR-7058-5p	0	1	2	0	0	3	2	1	1	1	1	5
mmu-miR-6240	108	71	187	105	931	1096	37	30	51	30	115	699
mmu-miR-30a-5p	37303	43257	38697	45280	37167	35516	31720	40597	38548	37457	35247	39290
mmu-miR-128-3p	136	185	136	173	95	93	134	111	117	135	97	105
mmu-miR-99a-3p	2	8	9	4	7	6	11	14	13	1	12	3
mmu-miR-7010-5p	0	0	0	0	0	1	0	1	1	1	1	0
mmu-miR-302c-3p	16	10	10	1	10	10	17	3	1	0	7	8
mmu-miR-1893	1	0	1	1	10	0	0	1	0	0	0	1
mmu-miR-540-3p	2	1	1	0	3	3	0	2	0	0	1	2
mmu-miR-344h-3p	1	1	0	0	1	0	0	0	0	0	0	1
mmu-miR-6933-3p	1	1	0	0	1	0	0	0	0	0	0	1
mmu-miR-540-5p	2	1	1	3	3	0	2	0	1	1	0	2
mmu-let-7c-2-3p	42	55	42	55	69	53	61	55	59	75	59	47
mmu-miR-344e-3p	0	0	0	1	0	1	0	1	0	0	0	0
mmu-let-7c-5p	5875	5017	5293	5304	2916	3174	5676	5107	5601	6805	4582	4133
mmu-miR-181b-2-3p	0	2	1	2	1	4	2	4	4	2	2	1
mmu-miR-101c	2	1	2	2	3	1	1	2	0	0	2	2
mmu-miR-767	0	0	0	0	0	0	0	0	0	0	1	7
mmu-miR-150-5p	270	263	253	207	171	141	343	171	142	104	144	119
mmu-miR-499-3p	4	4	6	3	7	6	7	3	11	4	8	5
mmu-miR-1192	2	1	3	2	10	6	2	2	3	1	4	3
mmu-miR-7044-5p	1	2	7	0	4	3	2	2	1	0	0	4
mmu-miR-29b-3p	87	91	70	81	55	37	117	66	77	99	67	75
mmu-miR-668-3p	1	1	0	2	0	1	0	0	1	1	1	0
mmu-miR-5616-5p	5	2	5	10	19	21	4	2	1	3	17	8
mmu-miR-3066-5p	0	6	3	1	0	0	2	2	4	4	1	3
mmu-miR-7014-5p	0	0	1	0	0	0	1	0	0	0	1	2
mmu-miR-532-3p	2	3	1	2	1	1	1	2	1	1	1	2
mmu-miR-770-3p	0	1	0	0	1	0	1	0	1	0	1	2
mmu-miR-103-3p	779	1021	860	1092	1113	1180	832	1311	1083	1241	1030	1176
mmu-miR-539-3p	0	0	0	0	1	1	0	0	0	0	1	0
mmu-miR-6715-3p	0	0	0	0	1	1	0	0	0	0	1	0
mmu-miR-6912-5p	0	0	0	0	1	1	0	0	0	0	1	0
mmu-miR-1953	0	0	1	0	1	0	0	1	0	0	0	0
mmu-miR-383-3p	0	2	1	0	0	0	1	0	0	0	0	0
mmu-miR-6972-5p	0	2	0	1	3	0	0	1	0	1	0	0
mmu-miR-3071-5p	15	15	20	22	25	16	17	21	28	49	15	20
mmu-miR-6949-3p	1	1	1	0	4	3	1	0	0	1	1	2
mmu-miR-6911-3p	0	4	0	1	3	0	2	2	3	2	1	2
mmu-miR-6903-3p	0	1	0	0	0	0	0	0	1	1	0	0
mmu-miR-5112	0	0	2	0	1	1	1	0	1	0	0	0
mmu-miR-10a-5p	8995	9951	9401	7889	8150	5780	7707	10743	7517	9443	10043	10019
mmu-miR-6924-3p	0	0	0	1	0	4	0	0	0	0	1	0
mmu-miR-3968	19	13	18	8	15	4	12	9	7	8	12	12
mmu-miR-6994-3p	0	1	1	0	0	1	0	1	0	2	1	1
mmu-miR-682	0	1	0	0	1	1	1	1	0	1	1	4
mmu-miR-7019-3p	1	0	1	0	0	0	2	0	3	0	1	0
mmu-miR-1955-5p	1	1	1	0	1	0	1	1	0	0	0	1
mmu-miR-7680-5p	0	1	0	0	0	0	0	0	1	0	0	1
mmu-miR-466i-5p	0	4	3	1	3	10	3	2	0	1	3	2
mmu-miR-100-3p	6	2	2	1	1	1	3	4	4	7	2	1
mmu-miR-1934-5p	0	1	0	0	1	3	1	1	0	1	0	0
mmu-miR-7011-5p	0	0	0	0	1	0	1	1	0	0	1	2
mmu-miR-5621-5p	2	1	5	1	13	22	1	0	0	0	0	16
mmu-miR-7678-5p	1	0	1	0	0	0	0	1	0	0	0	0
mmu-miR-7680-3p	0	1	1	0	0	1	0	0	0	0	0	1
mmu-miR-300-5p	0	0	0	1	1	0	0	0	1	1	1	1
mmu-miR-1895	10	4	18	4	9	7	4	6	6	7	9	7
mmu-miR-28b	0	0	0	1	0	0	0	1	0	0	1	1
mmu-miR-144-5p	146	176	135	207	129	217	244	199	205	211	155	137
mmu-miR-22-5p	29	23	27	14	31	12	29	17	13	14	23	11
mmu-miR-3089-5p	4	1	0	0	1	0	0	0	0	0	1	1
mmu-miR-149-3p	1	0	0	0	1	0	0	0	0	0	1	0
mmu-miR-3971	1	0	0	0	1	0	0	0	0	0	1	0
mmu-miR-6921-3p	1	0	0	0	0	1	0	0	0	0	1	0
mmu-miR-7093-3p	0	0	0	0	1	4	1	1	0	0	0	0
mmu-miR-130b-5p	1	1	0	3	0	1	1	1	0	2	0	0
mmu-miR-7228-5p	0	0	0	1	0	0	0	0	0	1	1	1
mmu-miR-3090-5p	1	0	0	0	1	1	0	0	0	1	1	0
mmu-miR-328-5p	0	0	3	0	0	4	0	0	1	0	1	0
mmu-miR-369-3p	1	1	1	0	6	4	1	1	0	1	2	2
mmu-miR-7087-5p	0	0	0	0	1	3	0	0	0	0	0	1
mmu-miR-1224-5p	0	0	0	0	3	1	0	0	0	0	0	1

NGS miR (WT = 1,3,5,7,9,11; KO = 2,4,6,8,10,12)

mmu-miR-7045-3p	0	0	1	1	1	0	1	2	1	0	1	0
mmu-miR-203-3p	88	122	84	88	77	82	70	107	96	72	72	66
mmu-miR-219a-5p	1	3	1	2	0	3	2	1	0	1	1	1
mmu-miR-17-3p	11	5	8	8	3	9	9	4	1	2	4	11
mmu-miR-1931	0	1	0	0	0	0	1	0	1	0	1	0
mmu-miR-223-5p	4	7	9	3	24	13	2	8	8	3	11	7
mmu-miR-5125	1	0	0	1	4	0	0	0	0	1	0	1
mmu-miR-20b-5p	0	1	0	0	0	1	0	2	0	0	2	2
mmu-miR-3060-3p	0	2	2	2	1	1	1	1	1	3	4	2
mmu-miR-568	0	1	0	0	0	0	0	0	0	1	0	1
mmu-miR-3473f	0	0	0	0	0	1	1	0	0	1	0	3
mmu-miR-8111	0	3	1	2	1	4	2	2	1	7	1	4
mmu-miR-679-3p	4	0	6	2	10	4	4	3	11	2	12	9
mmu-miR-384-3p	0	1	0	0	3	0	0	1	0	0	0	0
mmu-miR-6996-3p	0	0	0	1	0	1	0	0	0	0	1	0
mmu-miR-1940	14	4	10	3	46	31	6	5	10	10	10	23
mmu-miR-466i-3p	1	0	0	0	1	4	0	0	0	0	1	1
mmu-miR-574-3p	19	15	15	21	10	15	19	16	27	27	15	10
mmu-miR-1943-3p	0	1	1	0	4	1	2	0	0	0	0	1
mmu-miR-6372	0	0	2	0	0	0	0	1	3	1	1	0
mmu-miR-3086-5p	0	1	0	0	0	1	0	1	0	1	1	1
mmu-let-7i-5p	2628	2998	2949	2981	1241	1486	2548	3521	3239	3360	2017	2153
mmu-miR-194-2-3p	1	2	4	5	1	4	1	5	0	0	3	3
mmu-miR-20a-5p	151	192	175	180	127	97	160	140	150	138	110	124
mmu-miR-6343	1	0	0	1	0	0	0	1	1	1	1	0
mmu-miR-7043-3p	0	1	1	2	1	1	1	0	0	0	2	1
mmu-miR-106a-3p	0	0	0	0	0	0	1	0	0	0	0	0
mmu-miR-1247-3p	0	0	0	0	0	0	0	1	0	0	0	0
mmu-miR-1251-3p	0	0	0	0	0	0	0	0	0	0	1	0
mmu-miR-1258-5p	0	0	0	0	0	0	0	0	0	0	0	1
mmu-miR-1897-3p	0	0	0	0	0	0	0	0	0	0	0	1
mmu-miR-1904	0	0	0	0	0	0	0	0	0	0	1	0
mmu-miR-190b-3p	0	0	0	0	0	0	0	0	0	1	0	0
mmu-miR-1952	0	0	0	0	0	0	1	0	0	0	0	0
mmu-miR-1968-3p	0	0	0	0	0	0	0	0	0	0	2	0
mmu-miR-200c-5p	0	0	0	0	0	0	0	0	0	0	0	1
mmu-miR-205-5p	0	0	0	0	0	0	0	0	0	0	0	1
mmu-miR-2139	0	0	0	0	0	0	0	1	0	0	0	0
mmu-miR-216a-3p	0	0	0	0	0	0	0	0	0	0	1	0
mmu-miR-216b-3p	0	0	0	0	0	0	0	0	0	0	1	0
mmu-miR-217-3p	0	0	0	0	0	0	1	0	0	0	0	0
mmu-miR-217-5p	0	0	0	0	0	0	0	0	0	0	1	0
mmu-miR-291a-3p	0	0	0	0	0	0	0	0	0	0	0	1
mmu-miR-292b-3p	0	0	0	0	0	0	0	1	0	0	0	0
mmu-miR-292b-5p	0	0	0	0	0	0	0	0	0	0	0	1
mmu-miR-294-5p	0	0	0	0	0	0	0	0	0	0	0	1
mmu-miR-297c-5p	0	0	0	0	0	0	0	0	0	0	1	0
mmu-miR-301b-5p	0	0	0	0	0	0	1	0	0	0	0	0
mmu-miR-3067-5p	0	0	0	0	0	0	0	0	0	1	0	0
mmu-miR-3070a-3p	0	0	0	0	0	0	0	0	0	0	0	1
mmu-miR-3083-3p	0	0	0	0	0	0	1	0	0	0	0	0
mmu-miR-3085-5p	0	0	0	0	0	0	0	0	0	0	1	0
mmu-miR-3086-3p	0	0	0	0	0	0	0	0	0	0	0	1
mmu-miR-3094-3p	0	0	0	0	0	0	0	1	0	0	0	0
mmu-miR-3095-3p	0	0	0	0	0	0	0	0	0	2	0	0
mmu-miR-3099-3p	0	0	0	0	0	0	1	0	0	0	0	0
mmu-miR-3103-5p	0	0	0	0	0	0	0	0	0	0	1	0
mmu-miR-326-5p	0	0	0	0	0	0	0	1	0	0	0	0
mmu-miR-344b-5p	0	0	0	0	0	0	1	0	0	0	0	0
mmu-miR-344d-2-5p	0	0	0	0	0	0	1	0	0	0	0	0
mmu-miR-3471	0	0	0	0	0	0	0	0	0	0	1	0
mmu-miR-3475-5p	0	0	0	0	0	0	0	0	0	0	0	1
mmu-miR-363-5p	0	0	0	0	0	0	1	0	0	0	0	0
mmu-miR-367-3p	0	0	0	0	0	0	0	0	0	0	0	1
mmu-miR-377-5p	0	0	0	0	0	0	0	1	0	0	0	0
mmu-miR-381-5p	0	0	0	0	0	0	0	1	0	0	0	0
mmu-miR-421-5p	0	0	0	0	0	0	0	0	0	0	1	0
mmu-miR-432	0	0	0	0	0	0	0	1	0	0	0	0
mmu-miR-449c-5p	0	0	0	0	0	0	0	1	0	0	0	0
mmu-miR-465a-5p	0	0	0	0	0	0	0	0	0	0	1	0
mmu-miR-466a-3p	0	0	0	0	0	0	0	0	0	0	1	0
mmu-miR-466b-5p	0	0	0	0	0	0	1	0	0	0	0	0
mmu-miR-466h-5p	0	0	0	0	0	0	0	0	0	0	1	0
mmu-miR-466m-5p	0	0	0	0	0	0	0	0	1	0	0	0
mmu-miR-466o-5p	0	0	0	0	0	0	0	0	0	0	1	0
mmu-miR-467d-3p	0	0	0	0	0	0	1	0	0	0	0	0
mmu-miR-489-3p	0	0	0	0	0	0	0	0	0	0	0	1
mmu-miR-491-5p	0	0	0	0	0	0	0	1	0	0	0	0
mmu-miR-496a-5p	0	0	0	0	0	0	0	1	0	0	0	0
mmu-miR-5124b	0	0	0	0	0	0	0	0	0	0	0	1
mmu-miR-5136	0	0	0	0	0	0	0	0	0	0	1	0
mmu-miR-543-5p	0	0	0	0	0	0	0	0	0	1	0	0
mmu-miR-5615-5p	0	0	0	0	0	0	1	0	0	0	0	0
mmu-miR-5617-5p	0	0	0	0	0	0	0	0	0	0	1	0
mmu-miR-5619-5p	0	0	0	0	0	0	0	0	0	0	1	0
mmu-miR-5620-3p	0	0	0	0	0	0	0	0	0	1	0	0
mmu-miR-5620-5p	0	0	0	0	0	0	0	0	0	0	0	1
mmu-miR-5625-3p	0	0	0	0	0	0	1	0	0	0	0	0
mmu-miR-599	0	0	0	0	0	0	0	0	0	0	0	1
mmu-miR-6336	0	0	0	0	0	0	0	0	0	0	1	0
mmu-miR-6364	0	0	0	0	0	0	0	0	0	0	1	0
mmu-miR-6385	0	0	0	0	0	0	0	0	0	0	0	1
mmu-miR-6391	0	0	0	0	0	0	0	0	0	0	1	0
mmu-miR-6392-3p	0	0	0	0	0	0	0	1	0	0	0	0
mmu-miR-6402	0	0	0	0	0	0	0	0	0	0	1	0
mmu-miR-6410	0	0	0	0	0	0	0	0	0	0	1	0
mmu-miR-6414	0	0	0	0	0	0	0	0	1	0	0	0

NGS miR (WT = 1,3,5,7,9,11; KO = 2,4,6,8,10,12)

mmu-miR-6419	0	0	0	0	0	0	0	0	0	0	1	0
mmu-miR-6537-3p	0	0	0	0	0	0	1	0	0	0	0	0
mmu-miR-669i	0	0	0	0	0	0	0	0	0	0	1	0
mmu-miR-670-5p	0	0	0	0	0	0	0	1	0	0	0	0
mmu-miR-672-3p	0	0	0	0	0	0	0	0	0	0	0	1
mmu-miR-678	0	0	0	0	0	0	0	0	0	1	0	0
mmu-miR-684	0	0	0	0	0	0	1	0	0	0	0	0
mmu-miR-6920-3p	0	0	0	0	0	0	0	0	0	0	1	0
mmu-miR-6926-3p	0	0	0	0	0	0	1	0	0	0	0	0
mmu-miR-6939-3p	0	0	0	0	0	0	1	0	0	0	0	0
mmu-miR-6941-5p	0	0	0	0	0	0	0	0	0	0	0	1
mmu-miR-696	0	0	0	0	0	0	1	0	0	0	0	0
mmu-miR-6960-3p	0	0	0	0	0	0	1	0	0	0	0	0
mmu-miR-6967-3p	0	0	0	0	0	0	1	0	0	0	0	0
mmu-miR-6974-5p	0	0	0	0	0	0	0	0	0	0	1	0
mmu-miR-6977-5p	0	0	0	0	0	0	0	0	0	0	1	0
mmu-miR-6978-3p	0	0	0	0	0	0	0	0	0	0	1	0
mmu-miR-6979-3p	0	0	0	0	0	0	1	0	0	0	0	0
mmu-miR-6979-5p	0	0	0	0	0	0	0	0	0	1	0	0
mmu-miR-6983-5p	0	0	0	0	0	0	0	0	0	0	0	1
mmu-miR-698-3p	0	0	0	0	0	0	1	0	0	0	0	0
mmu-miR-6986-3p	0	0	0	0	0	0	0	0	0	0	1	0
mmu-miR-6986-5p	0	0	0	0	0	0	0	1	0	0	0	0
mmu-miR-6999-5p	0	0	0	0	0	0	0	1	0	0	0	0
mmu-miR-7004-3p	0	0	0	0	0	0	1	0	0	0	0	0
mmu-miR-7006-3p	0	0	0	0	0	0	1	0	0	0	0	0
mmu-miR-7008-5p	0	0	0	0	0	0	0	0	0	0	0	1
mmu-miR-7012-3p	0	0	0	0	0	0	0	0	0	1	0	0
mmu-miR-7013-3p	0	0	0	0	0	0	1	0	0	0	0	0
mmu-miR-702-5p	0	0	0	0	0	0	0	0	1	0	0	0
mmu-miR-7030-5p	0	0	0	0	0	0	0	0	0	0	1	0
mmu-miR-7035-3p	0	0	0	0	0	0	0	0	0	1	0	0
mmu-miR-7040-3p	0	0	0	0	0	0	0	0	0	0	0	1
mmu-miR-7041-5p	0	0	0	0	0	0	0	0	1	0	0	0
mmu-miR-7054-3p	0	0	0	0	0	0	0	1	0	0	0	0
mmu-miR-7061-5p	0	0	0	0	0	0	0	1	0	0	0	0
mmu-miR-7065-5p	0	0	0	0	0	0	0	0	0	0	1	0
mmu-miR-707	0	0	0	0	0	0	0	1	0	0	0	0
mmu-miR-7070-3p	0	0	0	0	0	0	0	0	0	0	0	1
mmu-miR-7076-5p	0	0	0	0	0	0	1	0	0	0	0	0
mmu-miR-7077-5p	0	0	0	0	0	0	0	0	0	0	1	0
mmu-miR-7084-3p	0	0	0	0	0	0	1	0	0	0	0	0
mmu-miR-7085-5p	0	0	0	0	0	0	0	1	0	0	0	0
mmu-miR-7094b-2-5p	0	0	0	0	0	0	0	1	0	0	0	0
mmu-miR-7119-5p	0	0	0	0	0	0	0	0	0	0	1	0
mmu-miR-718	0	0	0	0	0	0	0	0	0	0	1	0
mmu-miR-7216-3p	0	0	0	0	0	0	0	0	0	0	1	0
mmu-miR-7219-3p	0	0	0	0	0	0	0	0	0	1	0	0
mmu-miR-7224-5p	0	0	0	0	0	0	0	0	0	0	0	1
mmu-miR-7228-3p	0	0	0	0	0	0	0	0	0	0	0	1
mmu-miR-7229-5p	0	0	0	0	0	0	0	0	0	1	0	0
mmu-miR-7234-3p	0	0	0	0	0	0	0	1	0	0	0	0
mmu-miR-7241-3p	0	0	0	0	0	0	0	0	0	0	1	0
mmu-miR-741-5p	0	0	0	0	0	0	0	0	0	0	1	0
mmu-miR-743b-5p	0	0	0	0	0	0	1	0	0	0	0	0
mmu-miR-7647-3p	0	0	0	0	0	0	0	0	1	0	0	0
mmu-miR-7658-3p	0	0	0	0	0	0	0	1	0	0	0	0
mmu-miR-7664-5p	0	0	0	0	0	0	0	0	0	0	0	1
mmu-miR-7666-3p	0	0	0	0	0	0	0	1	0	0	0	0
mmu-miR-7668-3p	0	0	0	0	0	0	0	1	0	0	0	0
mmu-miR-7672-3p	0	0	0	0	0	0	0	1	0	0	0	0
mmu-miR-7676-5p	0	0	0	0	0	0	0	0	0	0	1	0
mmu-miR-7689-3p	0	0	0	0	0	0	1	0	0	0	0	0
mmu-miR-8092	0	0	0	0	0	0	0	0	0	0	1	0
mmu-miR-8096	0	0	0	0	0	0	1	0	0	0	0	0
mmu-miR-8100	0	0	0	0	0	0	0	0	0	0	0	1
mmu-miR-873b	0	0	0	0	0	0	0	0	0	0	1	0
mmu-miR-880-3p	0	0	0	0	0	0	0	0	0	1	0	0
mmu-miR-881-5p	0	0	0	0	0	0	0	0	0	0	1	0
mmu-miR-883a-5p	0	0	0	0	0	0	0	0	0	1	0	0
mmu-miR-92a-2-5p	0	0	0	0	0	0	0	1	0	0	0	0
mmu-miR-138-2-3p	0	0	1	0	0	0	0	0	0	0	0	0
mmu-miR-146a-3p	0	0	0	0	7	0	0	0	0	0	0	0
mmu-miR-146b-3p	0	0	0	0	1	0	0	0	0	0	0	0
mmu-miR-155-3p	0	1	0	0	0	0	0	0	0	0	0	0
mmu-miR-1896	0	0	0	0	1	0	0	0	0	0	0	0
mmu-miR-1899	0	0	0	0	0	1	0	0	0	0	0	0
mmu-miR-18b-3p	0	0	1	0	0	0	0	0	0	0	0	0
mmu-miR-18b-5p	0	1	0	0	0	0	0	0	0	0	0	0
mmu-miR-1912-5p	0	0	0	0	0	1	0	0	0	0	0	0
mmu-miR-1936	0	0	0	0	0	1	0	0	0	0	0	0
mmu-miR-193b-5p	0	1	0	0	0	0	0	0	0	0	0	0
mmu-miR-1951	0	0	0	0	3	0	0	0	0	0	0	0
mmu-miR-1966-5p	0	1	0	0	0	0	0	0	0	0	0	0
mmu-miR-196a-1-3p	0	0	0	0	0	1	0	0	0	0	0	0
mmu-miR-1971	0	1	0	0	0	0	0	0	0	0	0	0
mmu-miR-19b-1-5p	0	0	0	0	0	1	0	0	0	0	0	0
mmu-miR-202-5p	0	0	1	0	0	0	0	0	0	0	0	0
mmu-miR-216a-5p	0	1	0	0	0	0	0	0	0	0	0	0
mmu-miR-224-3p	0	0	1	0	0	0	0	0	0	0	0	0
mmu-miR-290b-5p	0	0	0	0	0	1	0	0	0	0	0	0
mmu-miR-291b-3p	0	0	0	2	0	0	0	0	0	0	0	0
mmu-miR-291b-5p	0	0	1	0	0	0	0	0	0	0	0	0
mmu-miR-293-3p	0	0	0	0	0	1	0	0	0	0	0	0
mmu-miR-294-3p	0	0	0	1	0	0	0	0	0	0	0	0
mmu-miR-3062-3p	0	0	0	0	1	0	0	0	0	0	0	0
mmu-miR-3063-3p	0	0	0	0	1	0	0	0	0	0	0	0

NGS miR (WT = 1,3,5,7,9,11; KO = 2,4,6,8,10,12)

mmu-miR-3070b-3p	0	0	1	0	0	0	0	0	0	0	0	0
mmu-miR-3073a-3p	1	0	0	0	0	0	0	0	0	0	0	0
mmu-miR-3077-3p	0	0	1	0	0	0	0	0	0	0	0	0
mmu-miR-3077-5p	0	0	1	0	0	0	0	0	0	0	0	0
mmu-miR-3079-3p	0	1	0	0	0	0	0	0	0	0	0	0
mmu-miR-3080-3p	0	0	1	0	0	0	0	0	0	0	0	0
mmu-miR-3080-5p	0	0	1	0	0	0	0	0	0	0	0	0
mmu-miR-3088-3p	0	0	0	1	0	0	0	0	0	0	0	0
mmu-miR-3088-5p	0	0	0	0	1	0	0	0	0	0	0	0
mmu-miR-3090-3p	0	0	0	0	0	1	0	0	0	0	0	0
mmu-miR-3097-5p	0	0	1	0	0	0	0	0	0	0	0	0
mmu-miR-3099-5p	0	0	2	0	0	0	0	0	0	0	0	0
mmu-miR-3100-5p	0	1	0	0	0	0	0	0	0	0	0	0
mmu-miR-3101-3p	0	0	1	0	0	0	0	0	0	0	0	0
mmu-miR-3105-5p	0	1	0	0	0	0	0	0	0	0	0	0
mmu-miR-3110-3p	0	0	0	0	1	0	0	0	0	0	0	0
mmu-miR-3110-5p	0	0	0	0	1	0	0	0	0	0	0	0
mmu-miR-3113-5p	0	1	0	0	0	0	0	0	0	0	0	0
mmu-miR-329-3p	0	0	0	0	0	3	0	0	0	0	0	0
mmu-miR-343	0	0	1	0	0	0	0	0	0	0	0	0
mmu-miR-344-3p	1	0	0	0	0	0	0	0	0	0	0	0
mmu-miR-344f-3p	0	0	0	0	1	0	0	0	0	0	0	0
mmu-miR-3475-3p	0	1	0	0	0	0	0	0	0	0	0	0
mmu-miR-3572-5p	0	1	0	0	0	0	0	0	0	0	0	0
mmu-miR-3620-3p	0	1	0	0	0	0	0	0	0	0	0	0
mmu-miR-376c-5p	0	0	0	0	3	0	0	0	0	0	0	0
mmu-miR-380-5p	0	0	0	0	0	1	0	0	0	0	0	0
mmu-miR-3961	0	0	0	1	0	0	0	0	0	0	0	0
mmu-miR-465b-3p	0	0	0	0	0	1	0	0	0	0	0	0
mmu-miR-466d-3p	0	0	0	1	0	0	0	0	0	0	0	0
mmu-miR-466n-3p	0	0	0	0	0	1	0	0	0	0	0	0
mmu-miR-467b-3p	0	0	0	0	1	0	0	0	0	0	0	0
mmu-miR-467e-3p	0	0	0	0	0	1	0	0	0	0	0	0
mmu-miR-470-3p	0	0	0	1	0	0	0	0	0	0	0	0
mmu-miR-496b	0	0	1	0	0	0	0	0	0	0	0	0
mmu-miR-5103	0	1	0	0	0	0	0	0	0	0	0	0
mmu-miR-5118	0	0	0	1	0	0	0	0	0	0	0	0
mmu-miR-5130	0	0	0	0	0	1	0	0	0	0	0	0
mmu-miR-5134-5p	0	0	0	0	0	1	0	0	0	0	0	0
mmu-miR-539-5p	0	1	0	0	0	0	0	0	0	0	0	0
mmu-miR-541-3p	0	0	0	0	0	1	0	0	0	0	0	0
mmu-miR-546	0	0	1	0	0	0	0	0	0	0	0	0
mmu-miR-5627-5p	0	1	0	0	0	0	0	0	0	0	0	0
mmu-miR-590-5p	0	0	0	0	0	1	0	0	0	0	0	0
mmu-miR-615-5p	0	0	0	0	1	0	0	0	0	0	0	0
mmu-miR-6340	0	0	1	0	0	0	0	0	0	0	0	0
mmu-miR-6347	0	0	0	0	3	0	0	0	0	0	0	0
mmu-miR-6353	0	1	0	0	0	0	0	0	0	0	0	0
mmu-miR-6365	0	0	0	0	1	0	0	0	0	0	0	0
mmu-miR-6375	0	0	1	0	0	0	0	0	0	0	0	0
mmu-miR-6376	0	0	0	0	1	0	0	0	0	0	0	0
mmu-miR-6381	0	0	0	0	6	0	0	0	0	0	0	0
mmu-miR-6392-5p	0	1	0	0	0	0	0	0	0	0	0	0
mmu-miR-6394	0	0	0	0	1	0	0	0	0	0	0	0
mmu-miR-6404	0	0	0	0	0	1	0	0	0	0	0	0
mmu-miR-6413	1	0	0	0	0	0	0	0	0	0	0	0
mmu-miR-6416-3p	0	0	1	0	0	0	0	0	0	0	0	0
mmu-miR-6418-5p	1	0	0	0	0	0	0	0	0	0	0	0
mmu-miR-6537-5p	0	1	0	0	0	0	0	0	0	0	0	0
mmu-miR-654-3p	0	0	0	0	1	0	0	0	0	0	0	0
mmu-miR-654-5p	0	0	0	0	1	0	0	0	0	0	0	0
mmu-miR-667-5p	0	0	0	0	3	0	0	0	0	0	0	0
mmu-miR-669e-3p	0	0	0	0	1	0	0	0	0	0	0	0
mmu-miR-669e-5p	0	0	2	0	0	0	0	0	0	0	0	0
mmu-miR-669k-3p	1	0	0	0	0	0	0	0	0	0	0	0
mmu-miR-669l-3p	0	0	0	1	0	0	0	0	0	0	0	0
mmu-miR-669m-3p	0	0	1	0	0	0	0	0	0	0	0	0
mmu-miR-670-3p	0	0	0	0	1	0	0	0	0	0	0	0
mmu-miR-675-3p	0	1	0	0	0	0	0	0	0	0	0	0
mmu-miR-681	0	0	0	0	1	0	0	0	0	0	0	0
mmu-miR-6898-5p	1	0	0	0	0	0	0	0	0	0	0	0
mmu-miR-6900-5p	1	0	0	0	0	0	0	0	0	0	0	0
mmu-miR-6905-3p	0	0	0	0	1	0	0	0	0	0	0	0
mmu-miR-6907-3p	0	1	0	0	0	0	0	0	0	0	0	0
mmu-miR-6915-3p	0	0	0	0	1	0	0	0	0	0	0	0
mmu-miR-6917-5p	0	0	1	0	0	0	0	0	0	0	0	0
mmu-miR-6924-5p	0	0	1	0	0	0	0	0	0	0	0	0
mmu-miR-6926-5p	0	0	1	0	0	0	0	0	0	0	0	0
mmu-miR-6947-3p	0	0	0	0	0	1	0	0	0	0	0	0
mmu-miR-6951-5p	0	0	1	0	0	0	0	0	0	0	0	0
mmu-miR-6956-5p	0	1	0	0	0	0	0	0	0	0	0	0
mmu-miR-6965-3p	0	0	0	0	0	1	0	0	0	0	0	0
mmu-miR-6966-5p	0	0	0	1	0	0	0	0	0	0	0	0
mmu-miR-6967-5p	0	0	0	1	0	0	0	0	0	0	0	0
mmu-miR-6970-3p	0	0	0	0	1	0	0	0	0	0	0	0
mmu-miR-6971-5p	0	0	0	0	0	1	0	0	0	0	0	0
mmu-miR-6972-3p	0	0	0	0	1	0	0	0	0	0	0	0
mmu-miR-6982-5p	0	0	1	0	0	0	0	0	0	0	0	0
mmu-miR-6983-3p	0	0	1	0	0	0	0	0	0	0	0	0
mmu-miR-6984-5p	0	0	1	0	0	0	0	0	0	0	0	0
mmu-miR-6993-5p	0	1	0	0	0	0	0	0	0	0	0	0
mmu-miR-6995-5p	0	0	1	0	0	0	0	0	0	0	0	0
mmu-miR-6997-3p	0	1	0	0	0	0	0	0	0	0	0	0
mmu-miR-7000-3p	0	1	0	0	0	0	0	0	0	0	0	0
mmu-miR-7004-5p	0	0	0	0	0	1	0	0	0	0	0	0
mmu-miR-7005-5p	0	1	0	0	0	0	0	0	0	0	0	0
mmu-miR-7007-3p	0	0	1	0	0	0	0	0	0	0	0	0

NGS miR (WT = 1,3,5,7,9,11; KO = 2,4,6,8,10,12)

mmu-miR-7008-3p	0	0	0	0	0	1	0	0	0	0	0	0
mmu-miR-7016-5p	0	1	0	0	0	0	0	0	0	0	0	0
mmu-miR-7019-5p	0	0	0	0	0	1	0	0	0	0	0	0
mmu-miR-702-3p	0	0	0	0	1	0	0	0	0	0	0	0
mmu-miR-7024-3p	0	0	1	0	0	0	0	0	0	0	0	0
mmu-miR-7024-5p	0	1	0	0	0	0	0	0	0	0	0	0
mmu-miR-7026-5p	0	1	0	0	0	0	0	0	0	0	0	0
mmu-miR-7032-5p	1	0	0	0	0	0	0	0	0	0	0	0
mmu-miR-7041-3p	0	0	0	1	0	0	0	0	0	0	0	0
mmu-miR-7047-3p	0	0	0	0	1	0	0	0	0	0	0	0
mmu-miR-7050-3p	0	0	1	0	0	0	0	0	0	0	0	0
mmu-miR-7051-5p	0	0	1	0	0	0	0	0	0	0	0	0
mmu-miR-7052-3p	0	0	0	0	1	0	0	0	0	0	0	0
mmu-miR-7052-5p	0	0	0	0	1	0	0	0	0	0	0	0
mmu-miR-7053-5p	0	0	0	0	1	0	0	0	0	0	0	0
mmu-miR-7055-3p	0	1	0	0	0	0	0	0	0	0	0	0
mmu-miR-7060-3p	0	0	0	0	1	0	0	0	0	0	0	0
mmu-miR-7065-3p	0	0	0	1	0	0	0	0	0	0	0	0
mmu-miR-7067-3p	0	0	0	1	0	0	0	0	0	0	0	0
mmu-miR-7071-3p	0	0	0	0	1	0	0	0	0	0	0	0
mmu-miR-7074-5p	0	0	1	0	0	0	0	0	0	0	0	0
mmu-miR-7079-5p	0	1	0	0	0	0	0	0	0	0	0	0
mmu-miR-7080-3p	0	0	0	1	0	0	0	0	0	0	0	0
mmu-miR-7086-5p	0	0	0	0	0	1	0	0	0	0	0	0
mmu-miR-710	0	0	1	0	0	0	0	0	0	0	0	0
mmu-miR-712-3p	0	0	0	0	3	0	0	0	0	0	0	0
mmu-miR-7211-3p	1	0	0	0	0	0	0	0	0	0	0	0
mmu-miR-7211-5p	0	0	1	0	0	0	0	0	0	0	0	0
mmu-miR-7212-3p	0	0	0	0	1	0	0	0	0	0	0	0
mmu-miR-7212-5p	0	0	0	0	1	0	0	0	0	0	0	0
mmu-miR-7213-3p	0	1	0	0	0	0	0	0	0	0	0	0
mmu-miR-7218-5p	0	0	0	1	0	0	0	0	0	0	0	0
mmu-miR-7230-3p	0	0	0	0	0	3	0	0	0	0	0	0
mmu-miR-7231-5p	0	0	0	0	1	0	0	0	0	0	0	0
mmu-miR-7233-5p	0	0	0	0	1	0	0	0	0	0	0	0
mmu-miR-7236-5p	0	0	0	0	0	4	0	0	0	0	0	0
mmu-miR-7239-5p	0	1	0	0	0	0	0	0	0	0	0	0
mmu-miR-7241-5p	0	0	0	0	1	0	0	0	0	0	0	0
mmu-miR-7242-5p	0	0	0	0	1	0	0	0	0	0	0	0
mmu-miR-760-5p	0	0	0	0	1	0	0	0	0	0	0	0
mmu-miR-7654-5p	0	0	0	0	0	1	0	0	0	0	0	0
mmu-miR-7655-5p	0	0	0	0	1	0	0	0	0	0	0	0
mmu-miR-7662-3p	0	0	1	0	0	0	0	0	0	0	0	0
mmu-miR-7664-3p	0	0	0	0	0	1	0	0	0	0	0	0
mmu-miR-7666-5p	0	0	0	0	0	1	0	0	0	0	0	0
mmu-miR-7671-5p	0	1	0	0	0	0	0	0	0	0	0	0
mmu-miR-7673-5p	0	1	0	0	0	0	0	0	0	0	0	0
mmu-miR-7674-3p	1	0	0	0	0	0	0	0	0	0	0	0
mmu-miR-7681-3p	0	0	0	0	1	0	0	0	0	0	0	0
mmu-miR-7681-5p	0	0	0	0	1	0	0	0	0	0	0	0
mmu-miR-7682-5p	0	0	0	0	0	1	0	0	0	0	0	0
mmu-miR-7685-3p	1	0	0	0	0	0	0	0	0	0	0	0
mmu-miR-7686-5p	0	0	0	1	0	0	0	0	0	0	0	0
mmu-miR-8090	0	0	1	0	0	0	0	0	0	0	0	0
mmu-miR-8099	0	0	0	0	1	0	0	0	0	0	0	0
mmu-miR-8115	0	1	0	0	0	0	0	0	0	0	0	0
mmu-miR-8120	1	0	0	0	0	0	0	0	0	0	0	0
mmu-miR-873a-3p	0	0	0	1	0	0	0	0	0	0	0	0
mmu-miR-880-5p	0	1	0	0	0	0	0	0	0	0	0	0
mmu-miR-96-3p	0	0	1	0	0	0	0	0	0	0	0	0
mmu-miR-96-5p	0	0	0	0	1	0	0	0	0	0	0	0
mmu-miR-7677-5p	0	1	0	1	4	0	1	0	0	0	1	1
mmu-miR-467e-5p	4	4	5	3	4	3	5	2	1	3	4	4
mmu-miR-669p-3p	0	0	1	0	0	4	2	1	1	3	1	1
mmu-miR-15b-5p	83	85	65	79	65	81	134	59	94	108	76	61
mmu-miR-1898	0	0	1	0	1	0	0	0	0	0	1	0
mmu-miR-450a-1-3p	0	0	1	0	1	0	0	0	0	0	1	0
mmu-miR-6962-5p	0	0	1	0	1	0	0	0	0	0	1	0
mmu-miR-6998-3p	0	0	1	0	1	0	0	0	0	0	1	0
mmu-miR-3474	1	0	0	0	0	0	1	1	1	0	0	0
mmu-miR-3069-3p	0	0	1	0	0	1	0	0	0	0	1	0
mmu-miR-6975-3p	0	0	1	0	0	1	0	0	0	0	1	0
mmu-miR-763	0	0	1	0	0	1	0	0	0	0	1	0
mmu-miR-423-5p	56	55	56	59	64	60	56	67	55	62	43	45
mmu-miR-3084-3p	1	1	0	0	0	0	1	1	1	1	1	0
mmu-miR-376a-5p	0	1	0	1	3	0	1	0	0	0	0	1
mmu-miR-669c-3p	0	1	0	1	3	1	1	1	0	0	1	0
mmu-miR-5132-5p	0	0	0	1	0	0	1	0	3	0	0	0
mmu-miR-376c-3p	0	3	1	0	0	1	1	1	0	0	0	1
mmu-miR-7227-5p	4	0	0	3	3	0	0	0	1	0	1	2
mmu-miR-7017-3p	0	1	0	0	0	0	1	0	0	0	2	0
mmu-miR-3075-5p	0	1	0	0	0	0	1	0	0	0	0	2
mmu-miR-7226-3p	0	0	1	0	0	1	0	0	0	0	3	4
mmu-miR-1191b-5p	4	1	5	0	6	4	2	5	0	2	1	3
mmu-miR-7002-3p	0	1	0	0	0	1	0	1	0	0	0	0
mmu-miR-148a-3p	5829	7315	6072	4059	7330	9780	5999	10013	7323	4594	8994	10606
mmu-miR-7056-5p	0	0	1	0	0	0	1	0	0	0	1	1
mmu-miR-452-5p	0	2	0	0	1	0	1	0	1	0	1	2
mmu-miR-3076-5p	1	4	0	0	4	7	1	0	0	0	6	1
mmu-miR-24-2-5p	478	301	326	354	216	184	505	343	379	364	339	243
mmu-miR-6344	1	1	1	0	0	0	0	1	0	0	0	1
mmu-miR-467d-5p	1	0	0	1	0	3	0	1	0	1	0	0
mmu-miR-3112-3p	0	1	0	1	1	0	0	0	0	1	1	0
mmu-miR-325-3p	0	1	2	0	1	0	0	0	0	0	1	1
mmu-miR-6976-3p	2	1	0	0	0	0	0	0	0	0	1	0
mmu-miR-6942-5p	0	1	1	0	0	0	1	0	1	0	1	0
mmu-miR-344i	0	0	1	1	0	1	1	0	0	0	0	1

NGS miR (WT = 1,3,5,7,9,11; KO = 2,4,6,8,10,12)

mmu-miR-1927	0	1	0	0	0	1	1	0	0	0	0	0
mmu-miR-5116	0	1	0	0	0	1	1	0	0	0	0	0
mmu-miR-5132-3p	0	1	0	0	0	1	1	0	0	0	0	0
mmu-miR-1199-5p	0	0	0	0	0	3	1	1	0	0	1	4
mmu-miR-142-3p	39	29	22	25	19	27	34	30	21	13	12	24
mmu-miR-6995-3p	0	0	0	1	1	3	0	0	1	0	0	1
mmu-miR-6935-3p	1	2	0	0	1	3	0	0	1	0	1	2
mmu-miR-344d-3p	0	1	0	0	1	1	1	1	1	3	1	0
mmu-miR-298-5p	4	6	11	10	4	10	6	6	10	20	9	9
mmu-miR-6943-3p	0	0	1	0	0	1	0	1	3	0	1	1
mmu-miR-652-3p	31	26	28	27	19	25	42	23	34	22	25	29
mmu-miR-7020-5p	0	0	1	1	0	0	0	0	0	0	1	0
mmu-miR-804	1	1	1	0	3	0	1	1	0	0	1	1
mmu-miR-342-5p	7	6	8	2	4	6	4	2	8	6	3	4
mmu-miR-370-5p	0	1	0	0	0	0	0	0	0	1	1	0
mmu-miR-106b-3p	36	36	45	42	47	68	51	51	39	38	35	28
mmu-miR-351-3p	1	0	1	0	0	0	1	0	1	3	0	0
mmu-miR-543-3p	2	1	1	0	1	0	1	0	1	0	0	1
mmu-miR-7063-5p	1	4	0	0	0	1	0	1	0	1	1	0
mmu-miR-200a-5p	0	1	0	0	0	0	0	1	0	0	1	0
mmu-miR-615-3p	0	1	0	0	0	0	0	0	0	0	1	1
mmu-miR-7085-3p	0	0	0	0	1	0	1	0	3	0	1	0
mmu-miR-484	70	74	65	89	55	60	79	58	65	71	56	49
mmu-miR-7039-5p	0	0	2	0	0	3	0	0	0	0	5	8
mmu-miR-21c	0	1	0	0	4	0	0	0	0	1	0	0
mmu-miR-107-5p	0	0	0	1	0	0	0	0	0	1	0	2
mmu-miR-511-3p	11	14	9	14	19	4	12	13	18	20	12	10
mmu-miR-653-5p	1	1	2	2	6	3	2	2	3	2	4	7
mmu-miR-7007-5p	0	0	1	2	0	6	1	1	0	0	1	0
mmu-miR-299a-3p	0	2	0	3	1	1	2	1	0	0	1	1
mmu-miR-495-3p	0	0	1	0	0	1	1	0	0	1	3	0
mmu-let-7c-1-3p	7	6	9	7	7	7	6	8	10	9	11	5
mmu-miR-6957-5p	0	0	0	0	1	1	0	0	0	0	0	1
mmu-miR-365-3p	26	29	28	24	25	28	36	34	25	27	30	21
mmu-miR-7075-3p	1	1	2	1	6	7	0	1	1	1	1	7
mmu-miR-7058-3p	1	3	2	1	3	0	1	1	1	0	2	1
mmu-miR-1956	1	1	0	0	0	1	0	0	0	0	1	0
mmu-miR-677-5p	156	112	144	170	297	287	157	124	247	175	390	355
mmu-miR-5709-3p	0	1	0	1	1	0	0	0	1	0	0	0
mmu-miR-1946a	0	3	6	2	6	0	2	1	3	2	9	12
mmu-miR-181b-5p	407	273	356	227	365	374	287	327	247	218	339	389
mmu-miR-7656-5p	0	1	0	1	0	1	0	0	1	0	0	0
mmu-miR-7234-5p	1	0	0	0	3	0	0	0	1	0	0	0
mmu-miR-511-5p	0	1	0	2	1	0	1	2	1	0	3	0
mmu-miR-497b	0	0	1	1	0	0	1	1	0	1	1	0
mmu-miR-6363	0	0	1	1	0	1	0	0	0	1	1	0
mmu-miR-879-5p	1	4	0	0	0	4	0	1	1	2	0	1
mmu-miR-486-3p	800	804	669	910	903	1161	665	1065	906	915	1030	1150
mmu-miR-7652-3p	0	1	0	0	0	0	1	0	0	1	0	0
mmu-miR-1960	4	5	3	4	0	3	1	3	6	6	4	4
mmu-miR-139-5p	42	39	39	41	33	24	36	30	34	37	30	35
mmu-miR-7048-3p	0	1	0	0	1	0	0	0	0	0	1	0
mmu-miR-1a-2-5p	4	0	3	0	0	3	0	0	0	0	3	2
mmu-miR-1932	0	1	0	0	0	1	0	0	0	0	1	0
mmu-miR-6929-5p	0	1	0	0	0	1	0	0	0	0	1	0
mmu-miR-676-5p	0	7	10	10	7	9	4	10	10	8	7	14
mmu-let-7e-3p	5	3	7	2	3	7	4	5	4	3	7	9
mmu-miR-219a-1-3p	4	6	2	1	4	6	3	4	7	6	4	4
mmu-miR-6953-3p	0	1	0	0	0	0	1	0	0	0	0	1
mmu-miR-425-3p	7	9	12	9	10	18	12	11	3	9	9	11
mmu-miR-5046	0	0	0	1	0	0	0	0	3	0	1	0
mmu-miR-145a-3p	208	218	157	204	126	103	210	124	166	176	122	87
mmu-let-7j	10172	12481	12158	12256	12380	14741	9805	13879	12886	14050	13496	14581
mmu-miR-6399	5	2	10	9	25	46	6	5	10	4	20	16
mmu-miR-489-5p	2	1	2	0	10	6	4	0	1	1	4	2
mmu-miR-493-5p	0	0	2	3	0	0	0	0	0	1	0	1
mmu-miR-7053-3p	0	1	1	0	0	0	1	0	1	1	0	0
mmu-miR-483-5p	0	0	0	1	1	1	0	0	1	0	1	0
mmu-miR-5621-3p	1	1	0	2	1	1	1	1	1	1	1	1
mmu-miR-367-5p	0	1	1	2	0	0	2	0	3	2	0	0
mmu-miR-140-3p	632	452	621	241	773	928	742	779	516	318	930	1096
mmu-miR-7042-5p	0	1	0	1	0	1	0	0	0	0	1	0
mmu-miR-6985-3p	5	4	2	3	3	6	2	4	4	3	4	14
mmu-miR-101a-3p	2346	2639	2890	2069	2638	3412	1962	3143	2236	1438	2714	2941
mmu-miR-6966-3p	0	1	1	0	0	0	0	1	0	0	1	2
mmu-miR-6957-3p	0	0	1	1	1	0	1	1	3	2	0	0
mmu-miR-3108-3p	0	0	0	0	1	1	0	1	0	0	1	0
mmu-miR-7061-3p	1	1	0	0	0	1	1	0	4	0	1	1
mmu-miR-181a-1-3p	24	27	15	18	25	28	27	30	25	27	21	20
mmu-miR-3082-5p	0	0	1	0	1	0	1	1	0	1	2	0
mmu-miR-1195	31	25	35	11	40	29	27	24	21	13	40	20
mmu-miR-6961-3p	0	0	1	1	3	0	0	0	0	0	2	0
mmu-miR-6904-3p	0	0	0	0	3	0	0	1	0	0	0	0
mmu-miR-466h-3p	0	0	0	0	0	3	0	1	0	0	0	0
mmu-miR-450b-3p	1	1	0	0	0	1	0	0	0	1	0	1
mmu-miR-1231-5p	1	1	0	1	0	0	0	0	1	0	0	0
mmu-miR-101b-3p	406	459	498	397	485	547	395	648	495	380	523	579
mmu-miR-375-3p	0	0	0	0	1	0	1	0	1	1	0	0
mmu-miR-18a-5p	1	4	4	2	6	3	6	3	1	6	4	5
mmu-miR-6955-5p	1	1	1	0	0	0	1	1	1	2	0	0
mmu-miR-466a-5p	1	1	0	0	0	0	0	0	0	0	1	0
mmu-miR-3473c	0	0	1	0	0	0	1	0	0	2	0	0
mmu-miR-6923-5p	0	0	0	0	1	1	1	0	0	0	1	0
mmu-miR-30c-2-3p	125	112	135	83	176	164	82	140	100	52	145	169
mmu-miR-551b-5p	0	0	0	0	1	0	1	0	1	0	0	1
mmu-miR-6997-5p	0	0	0	0	1	0	1	0	1	0	0	1
mmu-miR-3060-5p	0	0	0	0	3	0	1	0	0	0	0	0

NGS miR (WT = 1,3,5,7,9,11; KO = 2,4,6,8,10,12)

mmu-miR-1933-5p	0	1	0	0	1	0	1	1	0	0	1	1
mmu-miR-1968-5p	1	1	0	1	3	3	1	2	0	0	2	0
mmu-miR-301b-3p	2	9	5	10	3	3	6	9	11	12	1	2
mmu-miR-6950-3p	0	1	1	0	6	0	0	0	0	1	1	1
mmu-miR-5107-3p	0	0	0	0	0	1	0	1	1	1	0	0
mmu-miR-5110	0	1	3	0	1	0	1	0	0	0	1	1
mmu-miR-1933-3p	0	1	4	2	1	3	1	1	3	2	3	4
mmu-miR-191-3p	7	5	6	1	4	3	6	4	10	4	7	2
mmu-miR-7015-5p	0	0	1	0	0	0	1	0	0	0	0	1
mmu-miR-196a-5p	0	0	1	1	0	1	0	0	0	1	3	2
mmu-miR-302d-3p	10	10	14	0	10	10	16	4	4	1	11	5
mmu-miR-200c-3p	0	0	0	1	0	1	0	0	0	0	0	1
mmu-miR-7009-5p	0	0	0	1	0	1	0	0	0	0	0	1
mmu-miR-7073-5p	0	0	0	1	0	1	0	0	0	0	0	1
mmu-miR-8113	0	0	0	1	0	1	0	0	0	0	0	1
mmu-miR-30f	2	6	12	7	18	15	6	9	6	3	11	12
mmu-miR-542-5p	1	2	1	1	4	15	3	2	1	1	4	3
mmu-miR-6481	2	2	4	3	10	3	2	2	6	1	0	7
mmu-miR-24-1-5p	6	7	7	5	4	1	8	5	3	4	3	3
mmu-miR-485-5p	1	1	0	0	0	0	0	1	0	1	0	1
mmu-miR-5622-3p	0	1	0	5	1	0	4	1	0	1	2	4
mmu-miR-376a-3p	1	1	0	0	1	0	0	0	0	1	1	0
mmu-miR-376b-5p	2	4	3	9	0	0	2	2	1	1	4	2
mmu-miR-145b	0	1	2	1	3	1	1	1	0	0	1	3
mmu-miR-6951-3p	1	0	1	0	0	0	1	1	1	0	0	1
mmu-miR-1967	0	0	0	0	4	0	0	0	0	0	0	1
mmu-miR-3107-3p	245	99	76	83	296	401	106	107	97	74	248	280
mmu-miR-135a-1-3p	0	0	0	1	1	0	0	0	0	1	0	0
mmu-miR-181d-3p	0	0	0	1	1	0	0	0	0	1	0	0
mmu-miR-6906-5p	0	1	0	1	0	0	0	0	0	0	1	0
mmu-miR-202-3p	0	0	0	1	0	1	0	0	0	1	0	0
mmu-miR-374c-3p	0	0	0	1	0	1	0	0	0	1	0	0
mmu-miR-448-5p	0	0	0	1	0	1	0	0	0	1	0	0
mmu-miR-6977-3p	0	0	0	1	0	1	0	0	0	1	0	0
mmu-miR-1190	0	0	0	0	3	0	0	0	0	0	1	0
mmu-miR-6409	0	0	0	0	3	0	0	0	0	0	1	0
mmu-miR-7661-5p	1	2	2	3	7	0	1	1	3	2	1	3
mmu-miR-3074-1-3p	0	1	1	1	1	3	2	0	0	1	1	1
mmu-miR-21a-3p	26	8	11	4	22	28	22	14	4	7	40	47
mmu-miR-99b-5p	2526	2431	1723	2484	2258	1209	2741	1913	2611	2689	2357	1567
mmu-miR-6922-5p	0	0	1	0	1	0	0	0	0	0	0	1
mmu-miR-762	0	0	1	0	1	0	0	0	0	0	0	1
mmu-miR-210-3p	62	89	76	77	90	99	62	99	76	79	56	86
mmu-miR-148a-5p	44	41	35	21	46	60	29	63	60	38	43	46
mmu-miR-6993-3p	1	0	0	0	1	0	1	0	0	0	1	0
mmu-miR-3107-5p	50719	62080	49027	57886	58636	68033	49736	66882	60398	56763	57951	73547
mmu-miR-6992-3p	25	1	11	2	40	37	14	2	0	2	31	29
mmu-miR-16-2-3p	6	6	9	11	7	4	10	5	7	4	6	7
mmu-miR-3473e	0	3	1	1	1	1	2	0	0	1	3	4
mmu-miR-2137	9	2	11	3	24	28	7	3	3	1	11	27
mmu-miR-3072-3p	0	0	1	0	1	0	0	0	0	1	0	0
mmu-miR-149-5p	507	327	357	319	273	206	469	298	266	180	284	256
mmu-miR-1954	1	1	1	0	1	0	0	0	0	1	1	1
mmu-miR-669f-5p	1	1	0	3	0	0	0	2	0	0	1	0
mmu-miR-7215-3p	1	0	0	0	0	1	1	0	0	0	0	0
mmu-miR-129-5p	1	0	3	2	0	1	3	3	1	0	1	2
mmu-miR-6918-3p	4	1	2	1	1	0	1	1	0	1	1	2
mmu-miR-5119	0	1	1	0	0	0	0	0	0	0	1	0
mmu-miR-7652-5p	0	1	1	0	0	0	0	0	0	0	1	0
mmu-miR-7665-5p	0	0	0	2	0	0	1	0	0	0	0	0
mmu-miR-6239	4660	2203	4338	5017	11147	7570	2568	1963	4519	3080	7846	8039
mmu-miR-434-3p	22	10	11	4	13	15	16	16	15	14	13	11
mmu-miR-181c-3p	30	37	36	34	58	47	27	44	38	13	26	61
mmu-miR-208a-5p	40	24	22	30	18	15	39	21	17	13	21	15
mmu-miR-7079-3p	1	0	0	1	0	0	0	0	0	0	0	1
mmu-miR-1981-5p	2	1	2	2	1	4	2	3	0	10	3	2
mmu-miR-6962-3p	2	1	0	0	0	0	1	0	0	0	1	0
mmu-miR-6927-3p	0	1	0	0	0	0	0	0	0	0	0	2
mmu-miR-6901-5p	2	0	0	0	0	0	0	0	0	0	1	0
mmu-miR-3102-5p	0	0	1	0	0	3	0	0	0	0	1	1
mmu-miR-190a-5p	97	100	119	117	111	105	107	120	111	109	94	80
mmu-miR-6958-3p	1	1	2	0	0	4	1	1	0	1	1	2
mmu-miR-1231-3p	1	0	0	0	0	0	0	0	0	0	1	2
mmu-miR-877-5p	4	3	3	2	0	1	2	2	1	2	2	1
mmu-miR-6899-3p	4	2	2	2	0	0	1	3	0	1	1	0
mmu-miR-431-5p	2	3	3	2	1	4	2	3	3	4	3	3
mmu-miR-6929-3p	1	3	1	3	1	6	2	3	0	2	7	7
mmu-miR-421-3p	26	42	36	29	30	41	52	42	28	32	37	29
mmu-miR-423-3p	142	96	149	53	105	113	133	97	53	47	130	116
mmu-miR-8119	0	1	1	0	0	0	0	0	0	0	0	1
mmu-miR-142-5p	1014	1385	873	1172	978	1298	1530	1153	1026	1508	1030	978
mmu-miR-7668-5p	1	0	0	0	0	0	0	0	0	1	1	0
mmu-miR-6973b-3p	1	0	1	0	0	0	0	0	0	0	0	1
mmu-miR-5709-5p	2	2	2	1	4	0	1	3	3	0	1	1
mmu-miR-6989-3p	1	0	1	0	1	0	0	1	0	0	1	1
mmu-miR-370-3p	0	1	0	0	1	4	1	0	0	0	2	0
mmu-miR-142b	6	4	3	2	10	12	11	6	3	2	4	4
mmu-miR-3061-3p	9	5	2	3	6	3	4	6	0	2	8	2
mmu-miR-15a-5p	138	173	135	180	130	134	185	123	149	157	115	102
mmu-miR-7a-5p	54	40	48	56	38	28	41	47	44	68	51	38
mmu-miR-344g-3p	0	0	0	1	1	1	0	0	0	2	0	0
mmu-miR-669f-3p	0	1	0	0	0	0	1	0	0	2	0	0
mmu-miR-3057-5p	21	18	23	27	35	28	20	32	17	33	30	35
mmu-miR-207	0	0	0	0	3	4	2	0	0	0	1	0
mmu-miR-6975-5p	0	1	0	1	6	1	1	1	0	0	1	2
mmu-miR-674-5p	4	5	7	3	10	1	7	6	6	3	2	1
mmu-miR-3097-3p	1	0	1	0	0	1	0	1	0	1	1	0

NGS miR (WT = 1,3,5,7,9,11; KO = 2,4,6,8,10,12)

mmu-miR-196b-5p	6	5	6	1	3	4	2	1	1	3	7	7
mmu-miR-32-5p	10	10	11	12	9	7	11	9	15	4	5	8
mmu-miR-154-5p	1	3	5	4	0	0	1	2	1	1	1	3
mmu-miR-101b-5p	0	1	0	0	0	0	1	1	0	1	0	0
mmu-miR-190b-5p	12	13	16	11	15	13	14	17	10	10	12	13
mmu-miR-140-5p	83	102	83	91	98	110	106	102	104	82	102	92
mmu-miR-34a-5p	333	294	333	277	226	171	371	307	327	286	240	229
mmu-miR-6909-5p	0	0	0	0	1	3	1	0	0	0	1	1
mmu-miR-6946-5p	0	0	0	0	1	0	0	1	3	0	0	0
mmu-miR-676-3p	148	169	166	99	185	172	141	208	164	98	192	217
mmu-miR-3058-3p	1	1	1	2	0	0	1	1	1	0	1	4
mmu-miR-302a-3p	0	0	1	0	0	0	1	0	0	0	1	0
mmu-miR-7006-5p	0	0	1	0	0	0	1	0	1	0	0	0
mmu-miR-7059-3p	0	1	0	0	0	0	0	0	1	0	0	1
mmu-miR-6355	10	10	15	9	22	13	12	2	24	12	17	26
mmu-miR-7020-3p	0	0	3	0	0	0	0	0	0	0	0	1
mmu-miR-501-3p	10	6	4	8	7	1	7	6	7	10	8	3
mmu-miR-137-3p	1	0	0	1	1	0	0	0	0	0	0	2
mmu-miR-3544-5p	29	16	23	38	117	162	14	20	44	28	92	86
mmu-miR-3105-3p	0	0	2	0	0	0	0	0	0	0	1	0
mmu-miR-1b-3p	2	1	1	0	1	0	1	1	1	0	1	0
mmu-miR-329-5p	4	1	4	1	4	6	2	2	0	4	3	4
mmu-miR-130c	0	0	1	1	0	0	0	0	0	0	0	1
mmu-miR-6897-3p	0	0	1	1	0	0	0	0	0	0	0	1
mmu-miR-875-3p	0	0	0	0	3	0	0	0	0	1	0	0
mmu-miR-449a-5p	6	1	1	2	1	1	2	1	6	7	4	0
mmu-miR-3473g	0	1	1	0	1	0	1	1	0	0	1	0
mmu-miR-6969-3p	1	0	0	0	1	1	1	0	0	0	1	0
mmu-miR-6406	1	0	0	0	0	0	0	0	0	0	0	3
mmu-miR-466k	0	1	0	0	0	0	1	1	0	0	0	0
mmu-miR-6978-5p	0	2	0	0	0	0	0	0	0	0	1	0
mmu-miR-467g	0	1	2	0	0	1	0	0	0	0	1	1
mmu-miR-1839-3p	0	3	2	4	3	0	3	1	1	7	3	1
mmu-miR-200b-3p	0	1	1	0	0	0	1	1	0	0	1	0
mmu-miR-188-5p	0	0	0	1	1	0	0	0	1	0	1	1
mmu-miR-6945-3p	0	0	0	0	1	0	0	1	0	0	0	0
mmu-miR-7083-3p	1	0	1	0	0	0	2	1	0	1	0	0
mmu-miR-297a-5p	0	0	0	0	0	1	0	1	0	0	0	0
mmu-miR-7060-5p	0	0	0	0	0	1	0	1	0	0	0	0
mmu-miR-721	0	0	0	0	0	1	0	1	0	0	0	0
mmu-miR-7650-5p	0	0	0	0	0	1	0	1	0	0	0	0
mmu-miR-17-5p	41	65	40	46	47	32	58	38	46	43	37	29
mmu-miR-7117-5p	2	0	1	1	0	0	0	1	0	1	1	0
mmu-miR-215-3p	0	0	1	2	0	1	0	2	1	1	0	2
mmu-miR-5624-3p	0	1	0	0	1	0	0	0	0	0	0	1
mmu-miR-741-3p	0	1	0	0	1	0	0	0	0	0	0	1
mmu-miR-345-5p	15	17	22	19	9	9	16	16	14	12	12	12
mmu-miR-7674-5p	0	0	0	1	1	0	0	0	0	0	1	4
mmu-miR-206-3p	4	4	1	1	21	18	7	4	7	10	15	18
mmu-miR-1958	0	0	0	0	3	3	0	0	0	1	1	1
mmu-miR-224-5p	14	1	4	4	4	1	7	2	6	11	8	2
mmu-miR-6953-5p	0	0	0	1	1	0	0	0	1	0	0	0
mmu-miR-708-5p	0	1	0	1	0	0	2	0	0	0	0	1
mmu-miR-26b-5p	3120	3880	3571	3920	3392	4108	3262	3921	3693	4882	3353	3783
mmu-miR-6341	0	0	2	1	3	0	1	0	0	2	5	1
mmu-miR-6950-5p	0	1	1	0	0	0	1	0	0	0	1	0
mmu-miR-669a-3p	1	0	0	1	1	0	2	0	1	0	0	2
mmu-miR-6538	2	3	7	0	4	6	0	1	0	1	0	13
mmu-miR-7023-5p	0	0	0	0	1	0	1	0	0	0	0	0
mmu-miR-7676-3p	0	0	0	0	1	0	1	0	0	0	0	0
mmu-miR-3969	7	1	7	9	13	10	2	4	14	8	13	16
mmu-miR-6769b-5p	0	0	0	0	0	1	1	0	0	0	0	0
mmu-miR-6927-5p	0	0	0	0	0	1	1	0	0	0	0	0
mmu-miR-7662-5p	0	0	0	0	0	1	1	0	0	0	0	0
mmu-miR-7682-3p	1	0	1	0	0	0	1	0	0	0	1	0
mmu-miR-679-5p	0	1	1	0	1	3	2	1	0	0	3	2
mmu-miR-1187	1	0	1	1	4	0	2	2	0	0	1	1
mmu-miR-376b-3p	0	2	1	0	0	0	1	0	0	0	0	1
mmu-miR-299a-5p	0	1	1	0	0	0	1	0	0	2	1	0
mmu-miR-3068-5p	1	4	0	2	12	7	2	3	4	2	7	1
mmu-miR-3100-3p	0	0	0	1	3	0	0	0	0	0	0	2
mmu-miR-3098-3p	0	0	0	0	1	0	1	0	0	0	2	0
mmu-miR-3079-5p	1	0	0	0	1	0	0	1	0	0	0	1
mmu-miR-125a-5p	2608	2278	1780	2023	1493	881	2698	1461	1637	1739	1542	930
mmu-miR-488-3p	5	14	7	11	13	24	6	14	13	4	8	17
mmu-miR-3109-3p	0	2	3	0	4	0	1	1	0	1	2	2
mmu-miR-467a-5p	0	3	1	0	1	1	2	1	1	0	1	0
mmu-miR-6337	0	0	2	0	0	1	1	0	0	0	1	0
mmu-miR-30b-3p	17	25	20	25	24	35	20	22	14	23	23	33
mmu-miR-206-5p	1	1	2	2	6	1	1	1	1	1	10	3
mmu-miR-296-3p	5	1	2	0	3	6	1	1	1	1	4	4
mmu-miR-7214-3p	0	1	0	0	0	0	0	0	1	0	0	0
mmu-miR-6405	0	0	0	0	1	1	0	0	0	1	1	0
mmu-miR-1949	1	0	1	1	6	6	1	1	4	0	4	2
mmu-miR-697	0	0	0	0	1	0	0	0	0	1	1	0
mmu-miR-3078-5p	0	1	0	0	0	1	1	0	0	0	0	1
mmu-miR-6357	0	3	1	0	0	1	1	1	0	0	0	2
mmu-miR-3058-5p	0	1	0	0	0	0	0	1	0	0	0	0
mmu-miR-3089-3p	0	0	0	0	1	0	0	1	1	0	0	0
mmu-miR-468-5p	0	0	0	0	1	0	0	0	0	0	1	1
mmu-miR-19a-5p	0	1	0	0	0	1	1	0	1	1	0	0
mmu-miR-1945	1	0	0	0	0	0	0	1	0	0	0	0
mmu-miR-493-3p	1	0	0	0	0	0	0	1	0	0	0	0
mmu-miR-330-3p	1	0	1	0	3	0	0	1	0	0	1	2
mmu-miR-6963-3p	0	0	1	1	1	0	0	0	0	2	0	0
mmu-miR-1249-3p	12	7	11	4	9	18	7	8	10	8	9	14
mmu-miR-5128	5	6	3	8	6	9	9	4	6	9	7	5

NGS miR (WT = 1,3,5,7,9,11; KO = 2,4,6,8,10,12)

mmu-miR-491-3p	0	0	0	0	1	0	2	0	0	0	1	0
mmu-miR-9-3p	0	1	1	1	0	3	2	0	1	0	0	0
mmu-miR-29b-1-5p	2	3	1	0	1	1	1	2	1	1	2	1
mmu-miR-33-5p	2	1	0	1	0	1	1	1	0	0	1	1
mmu-miR-451b	1	0	0	0	0	0	0	0	1	0	1	0
mmu-miR-409-3p	1	1	1	1	3	1	1	1	1	6	1	1
mmu-miR-7044-3p	1	1	0	1	1	1	0	0	1	0	2	1
mmu-miR-98-3p	6	9	11	7	9	6	10	8	8	6	4	9
mmu-miR-222-5p	10	3	3	3	6	0	4	5	7	0	7	8
mmu-miR-6539	0	1	0	0	1	1	0	0	1	0	0	1
mmu-miR-7660-3p	1	0	0	1	0	0	0	0	1	0	0	0
mmu-miR-6911-5p	0	0	1	0	0	1	0	0	0	0	1	0
mmu-miR-1938	1	1	0	0	1	0	1	0	0	0	1	1
mmu-miR-5099	27301	15430	20821	21624	40820	49812	19059	17288	37681	24121	52433	47576
mmu-miR-6917-3p	5	1	2	0	0	0	1	1	1	0	2	1
mmu-miR-8095	0	0	0	0	3	0	0	0	0	0	1	0
mmu-miR-290a-3p	0	0	0	0	1	0	0	0	0	0	1	0
mmu-miR-298-3p	0	0	0	0	1	0	0	0	0	0	1	0
mmu-miR-7115-5p	0	0	0	0	1	0	0	0	0	0	1	0
mmu-miR-7659-3p	0	0	0	0	1	0	0	0	0	0	1	0
mmu-miR-875-5p	0	0	0	0	1	0	0	0	0	0	1	0
mmu-miR-6964-3p	0	1	3	0	1	1	2	0	0	0	1	2
mmu-miR-23a-5p	0	0	0	0	0	1	0	0	0	0	1	0
mmu-miR-28c	0	0	0	0	0	1	0	0	0	0	1	0
mmu-miR-3059-3p	0	0	0	0	0	1	0	0	0	0	1	0
mmu-miR-3569-3p	0	0	0	0	0	1	0	0	0	0	1	0
mmu-miR-463-3p	0	0	0	0	0	1	0	0	0	0	1	0
mmu-miR-487b-5p	0	0	0	0	0	1	0	0	0	0	1	0
mmu-miR-6416-5p	0	0	0	0	0	1	0	0	0	0	1	0
mmu-miR-6955-3p	0	0	0	0	0	1	0	0	0	0	1	0
mmu-miR-150-3p	1	3	3	1	3	0	2	2	1	2	1	3
mmu-miR-374c-5p	2	0	0	0	0	0	0	1	0	0	1	0
mmu-miR-451a	936	886	719	999	1071	1125	1648	698	732	859	706	644
mmu-miR-297b-5p	0	0	0	1	0	0	0	0	0	0	1	1
mmu-miR-7048-5p	0	0	0	1	0	0	0	0	0	0	1	1
mmu-miR-1224-3p	1	0	0	0	0	0	1	0	0	0	0	0
mmu-miR-1929-5p	1	0	0	0	0	0	1	0	0	0	0	0
mmu-miR-2183	1	0	0	0	0	0	1	0	0	0	0	0
mmu-miR-6965-5p	1	0	0	0	0	0	1	0	0	0	0	0
mmu-miR-342-3p	71	67	61	65	31	41	95	55	60	56	58	39
mmu-miR-7036-3p	0	1	0	0	1	0	1	0	0	0	0	0
mmu-miR-6931-5p	0	0	0	0	4	0	0	0	0	2	0	0
mmu-miR-384-5p	1	1	2	1	0	1	1	1	1	2	0	2
mmu-miR-6342	0	1	0	1	0	0	0	0	1	0	2	0
mmu-miR-1198-3p	0	0	1	1	0	0	1	1	0	1	0	0
mmu-miR-378d	12595	14670	15971	16530	16399	17945	10410	18719	15632	13370	14776	17442
mmu-miR-130a-3p	531	583	405	459	448	436	601	489	534	476	418	445
mmu-miR-5106	4	3	3	0	10	3	1	3	0	1	8	4
mmu-miR-411-3p	0	1	1	0	0	0	1	1	0	0	0	1
mmu-miR-455-5p	1	3	0	1	1	4	2	3	1	4	1	1
mmu-miR-106a-5p	0	1	0	0	0	0	1	1	0	0	1	0
mmu-miR-100-5p	451	516	356	443	470	346	514	316	509	341	459	321
mmu-miR-6968-5p	0	1	2	1	1	0	1	0	0	0	2	1
mmu-miR-5616-3p	1	1	0	1	10	7	0	0	4	1	12	11
mmu-miR-450a-5p	42	28	24	25	22	15	31	29	25	37	26	20
mmu-miR-7651-3p	0	2	1	3	0	1	0	2	0	1	7	1
mmu-miR-125b-2-3p	431	400	463	228	433	352	486	431	412	222	436	452
mmu-miR-6992-5p	0	0	0	0	0	1	1	0	0	1	0	0
mmu-miR-450b-5p	16	16	19	27	19	31	21	26	15	32	25	18
mmu-miR-7219-5p	0	0	0	0	1	0	1	0	0	1	0	0
mmu-miR-7213-5p	2	2	3	2	0	1	1	1	3	4	2	2
mmu-miR-466e-5p	0	0	0	1	0	0	0	1	0	0	0	0
mmu-miR-6934-5p	0	0	0	1	0	0	0	1	0	0	0	0
mmu-miR-802-3p	0	0	0	1	0	0	0	1	0	0	0	0
mmu-miR-16-5p	4808	5461	4051	5540	4361	4082	6294	4446	5150	5517	3996	4042
mmu-miR-7005-3p	0	0	0	0	1	1	0	1	1	0	0	0
mmu-miR-7026-3p	0	0	0	0	3	1	0	0	0	0	1	2
mmu-miR-6902-3p	0	0	0	0	0	3	2	0	0	0	1	1
mmu-miR-8093	0	1	0	1	0	0	0	0	0	0	0	1
mmu-miR-701-5p	0	0	1	0	1	1	1	0	0	0	0	2
mmu-miR-5120	0	0	1	0	0	1	0	1	0	0	0	1
mmu-miR-1894-3p	0	0	0	1	0	0	1	0	0	1	0	0
mmu-miR-7016-3p	0	1	0	0	0	0	0	1	0	0	1	0
mmu-miR-532-5p	51	42	71	34	75	72	46	77	51	42	70	85
mmu-miR-147-5p	0	0	0	1	3	0	1	0	1	0	1	0
mmu-miR-300-3p	15	14	17	9	12	15	4	9	20	11	10	21
mmu-miR-7083-5p	0	1	1	0	1	0	0	1	1	0	1	0
mmu-miR-6369	0	1	0	1	0	0	0	0	0	1	0	0
mmu-miR-6356	0	0	1	0	1	0	1	0	0	0	0	1
mmu-miR-1258-3p	0	1	0	0	3	0	0	0	0	0	2	0
mmu-miR-7068-5p	27	4	18	10	10	18	18	9	11	3	22	13
mmu-miR-3094-5p	0	0	0	0	3	1	0	1	0	0	1	1
mmu-miR-7038-3p	0	0	2	0	0	0	0	0	0	1	0	0
mmu-miR-1a-3p	227393	233955	235519	255857	166415	165774	213043	229406	222575	222668	199232	153896
mmu-miR-7226-5p	1	11	7	10	3	3	7	6	4	13	5	4
mmu-miR-669n	0	0	0	1	0	0	1	0	0	0	0	0
mmu-miR-466n-5p	0	1	0	0	0	0	0	0	0	1	0	0
mmu-miR-500-5p	0	1	0	0	0	0	0	0	0	1	0	0
mmu-miR-5617-3p	0	1	0	0	0	0	0	0	0	1	0	0
mmu-miR-5627-3p	0	1	0	0	0	0	0	0	0	1	0	0
mmu-miR-6916-3p	0	1	0	0	0	0	0	0	0	1	0	0
mmu-miR-7240-5p	0	1	0	0	0	0	0	0	0	1	0	0
mmu-miR-7670-5p	0	1	0	0	0	0	0	0	0	1	0	0
mmu-miR-383-5p	1	1	1	0	0	1	0	0	0	2	0	1
mmu-miR-1912-3p	0	1	0	1	1	0	0	0	0	0	2	0
mmu-miR-29a-3p	2268	1642	1692	1743	1241	1056	2455	1325	1477	1417	1200	1186
mmu-miR-3535	4836	1897	4191	3921	5320	6788	2651	2086	4030	2784	7411	5607

NGS miR (WT = 1,3,5,7,9,11; KO = 2,4,6,8,10,12)

mmu-miR-126a-3p	3474	3042	2884	3234	2001	1899	3820	2703	3006	3069	2629	2092
mmu-miR-2136	0	1	0	0	0	0	0	0	0	0	1	0
mmu-miR-325-5p	0	1	0	0	0	0	0	0	0	0	1	0
mmu-miR-1948-3p	2	1	1	0	0	0	2	0	0	1	0	0
mmu-miR-1969	0	0	0	1	0	0	0	1	0	0	0	1
mmu-miR-145a-5p	36	46	40	45	35	29	37	40	32	47	30	39
mmu-miR-5122	1	3	3	1	0	0	3	0	4	0	0	3
mmu-miR-665-5p	0	1	0	0	0	0	0	0	0	0	0	1
mmu-miR-8101	0	1	0	0	0	0	0	0	0	0	0	1
mmu-miR-3064-3p	0	0	1	0	0	0	0	1	0	0	0	0
mmu-miR-449a-3p	0	0	1	0	0	0	0	1	0	0	0	0
mmu-miR-205-3p	1	0	0	0	0	0	0	0	0	0	1	0
mmu-miR-341-5p	1	0	0	0	0	0	0	0	0	0	1	0
mmu-miR-5108	1	0	0	0	0	0	0	0	0	0	1	0
mmu-miR-6408	1	0	0	0	0	0	0	0	0	0	1	0
mmu-miR-6418-3p	1	0	0	0	0	0	0	0	0	0	1	0
mmu-miR-719	1	0	0	0	0	0	0	0	0	0	1	0
mmu-miR-7215-5p	1	0	0	0	0	0	0	0	0	0	1	0
mmu-miR-7217-5p	1	0	0	0	0	0	0	0	0	0	1	0
mmu-miR-331-5p	1	4	2	0	3	3	2	0	3	0	4	2
mmu-miR-6919-3p	0	0	0	1	0	1	0	0	0	1	1	0
mmu-miR-6980-3p	1	1	0	0	0	0	1	0	0	0	0	0
mmu-miR-26a-5p	41780	43608	36669	41547	29210	25345	46498	31615	35872	37899	29934	27568
mmu-miR-3572-3p	0	0	0	0	0	3	0	2	0	0	0	0
mmu-miR-7021-3p	0	1	1	0	0	0	0	0	0	0	0	1
mmu-miR-7687-5p	0	1	1	0	0	0	0	0	0	0	0	1
mmu-miR-346-3p	0	1	0	0	1	1	1	0	0	0	1	0
mmu-miR-6984-3p	1	0	0	0	0	0	0	0	0	1	1	0
mmu-miR-7088-3p	1	0	0	0	0	0	0	0	0	1	1	0
mmu-miR-122-5p	2	3	1	0	1	1	1	1	6	1	1	2
mmu-miR-674-3p	16	15	22	12	21	12	19	17	15	21	16	13
mmu-miR-186-5p	1177	1251	1066	1258	1116	1122	1219	1206	1157	1182	1172	1127
mmu-miR-6945-5p	0	1	0	0	0	1	0	0	1	0	0	0
mmu-let-7k	2	4	6	3	4	9	6	3	4	9	7	3
mmu-miR-5114	0	1	0	1	1	0	1	1	0	1	0	1
mmu-miR-27a-5p	17	18	13	12	16	13	16	17	8	11	15	18
mmu-miR-193a-5p	0	1	1	0	1	0	1	1	0	0	2	0
mmu-miR-324-5p	2	3	1	3	0	0	1	0	4	3	1	2
mmu-miR-187-5p	0	1	0	1	1	0	1	2	0	0	0	0
mmu-miR-7b-5p	15	16	18	31	4	4	17	15	14	28	3	0
mmu-miR-6932-3p	1	2	0	0	0	1	1	1	1	1	0	1
mmu-miR-339-3p	9	13	9	13	13	10	13	13	7	8	12	12
mmu-miR-7237-3p	0	0	1	0	1	0	0	0	0	0	1	1
mmu-miR-3473b	67	49	68	53	105	90	48	28	73	76	81	100
mmu-miR-7057-3p	0	1	0	1	1	1	0	2	1	0	1	0
mmu-miR-219c-3p	1	0	0	1	0	0	0	1	0	0	1	1
mmu-miR-3070a-5p	0	0	1	0	0	0	1	0	0	0	0	0
mmu-miR-7037-5p	0	0	1	0	0	0	1	0	0	0	0	0
mmu-miR-7077-3p	0	0	1	0	0	0	1	0	0	0	0	0
mmu-miR-7649-3p	0	0	1	0	0	0	1	0	0	0	0	0
mmu-miR-190a-3p	1	2	0	0	1	1	2	1	0	3	1	0
mmu-miR-1298-3p	0	0	1	0	1	0	0	0	0	1	1	0
mmu-miR-3965	1	1	1	1	1	1	0	3	0	2	1	2
mmu-miR-344c-3p	0	1	0	0	1	0	0	0	0	0	1	2
mmu-let-7f-2-3p	14	17	27	35	21	22	17	26	20	27	21	18
mmu-miR-3966	121	71	143	71	194	290	71	89	86	40	216	293
mmu-miR-32-3p	5	1	2	5	0	1	1	2	4	4	1	0
mmu-miR-7217-3p	0	0	0	0	0	1	1	0	0	0	1	0
mmu-miR-324-3p	4	3	3	3	3	3	2	1	0	6	4	8
mmu-miR-3059-5p	0	0	0	0	3	1	0	0	0	1	2	0
mmu-miR-3057-3p	1	1	1	1	0	1	0	1	0	0	0	3
mmu-miR-218-1-3p	0	1	1	0	0	0	0	1	0	0	0	0
mmu-miR-3103-3p	0	1	0	0	1	1	0	0	0	1	1	1
mmu-miR-6386	0	0	1	1	1	0	0	1	0	0	2	0
mmu-miR-3104-5p	0	1	1	0	0	0	0	0	1	0	1	0
mmu-miR-5121	952	408	766	733	912	912	769	333	825	399	1123	974
mmu-miR-873a-5p	0	0	1	0	0	0	1	0	0	0	1	0
mmu-miR-505-5p	0	0	1	0	1	0	1	0	0	0	1	1
mmu-miR-3473a	2	1	1	1	1	4	2	1	0	3	3	1
mmu-miR-6241	93	98	110	77	194	308	106	71	98	60	124	330
mmu-miR-374b-5p	91	84	54	87	59	62	98	68	76	79	69	61
mmu-miR-1901	0	0	0	1	0	0	0	0	0	0	1	0
mmu-miR-452-3p	0	0	0	1	0	0	0	0	0	0	1	0
mmu-miR-6411	0	0	0	1	0	0	0	0	0	0	1	0
mmu-miR-6981-3p	0	0	0	1	0	0	0	0	0	0	1	0
mmu-miR-7047-5p	0	0	0	1	0	0	0	0	0	0	1	0
mmu-miR-7227-3p	0	0	0	1	0	0	0	0	0	0	1	0
mmu-miR-7233-3p	0	0	0	0	0	1	0	1	0	0	1	0
mmu-miR-106b-5p	37	34	37	41	34	19	54	21	30	33	28	27
mmu-miR-6348	0	0	0	0	0	1	1	0	1	0	0	0
mmu-miR-16-1-3p	1	4	2	4	6	3	3	5	3	3	3	2
mmu-miR-141-3p	7	10	9	15	22	9	17	6	14	12	12	8
mmu-miR-7009-3p	1	1	2	0	1	0	0	2	0	0	6	0
mmu-miR-7090-5p	0	0	0	0	1	0	0	0	0	0	0	2
mmu-miR-7670-3p	0	0	0	0	1	0	0	0	0	0	0	2
mmu-miR-1903	0	0	1	0	3	1	1	1	0	0	2	1
mmu-miR-192-5p	998	1163	1111	1374	1184	1171	902	1298	1102	1331	1051	1186
mmu-miR-874-5p	1	1	3	4	1	0	5	1	1	1	0	1
mmu-miR-1198-5p	46	35	36	31	30	34	39	42	28	40	29	27
mmu-let-7f-5p	45771	53182	49420	55672	42756	48072	39891	48958	49252	59207	46279	46398
mmu-miR-8104	0	0	1	0	0	0	0	1	0	0	1	0
mmu-miR-7059-5p	1	0	0	0	1	0	0	0	3	0	1	0
mmu-miR-6956-3p	1	0	0	2	0	0	0	1	0	1	1	1
mmu-miR-7117-3p	1	1	1	0	0	0	0	1	0	1	1	0
mmu-miR-5133	1	1	1	0	0	0	0	0	0	2	0	0
mmu-miR-7014-3p	0	0	0	0	0	4	1	0	0	0	3	2
mmu-miR-338-3p	22	14	15	16	6	15	17	16	10	22	9	10

mmu-miR-3069-5p	0	1	0	0	1	0	0	1	0	0	0	1
mmu-miR-153-3p	6	6	11	7	4	4	10	8	8	3	3	8
mmu-miR-410-3p	9	11	17	8	6	10	8	8	17	6	14	11
mmu-miR-6964-5p	0	1	0	0	0	1	0	1	0	0	0	1
mmu-let-7g-5p	12836	12760	13343	14371	9662	10135	11272	12608	13031	15856	12070	9864
mmu-miR-7653-3p	0	1	1	1	0	0	1	1	1	0	0	0
mmu-miR-136-5p	2	8	9	11	4	7	5	6	3	13	9	9
mmu-miR-1934-3p	0	1	1	0	0	0	1	0	0	0	0	0
mmu-miR-143-3p	50925	38061	54614	24534	59952	72522	47787	58284	39293	22651	70115	77050
mmu-miR-669g	0	1	0	2	10	1	0	2	1	1	7	1
mmu-miR-7045-5p	1	3	0	1	1	0	2	1	1	0	1	1
mmu-miR-382-5p	0	0	2	0	3	0	0	0	0	2	1	2
mmu-miR-127-5p	0	1	2	0	1	3	1	0	1	0	5	1
mmu-miR-7088-5p	1	0	0	2	1	0	1	0	0	2	1	0
mmu-miR-363-3p	1	0	0	1	3	1	3	1	0	3	1	0
mmu-miR-6999-3p	0	0	0	0	1	0	0	1	0	0	1	0
mmu-miR-148b-5p	26	10	17	18	22	21	15	20	17	21	12	24
mmu-miR-2861	0	0	0	0	1	0	0	0	0	0	0	1
mmu-miR-3081-3p	0	0	0	0	1	0	0	0	0	0	0	1
mmu-miR-7034-5p	0	0	0	0	1	0	0	0	0	0	0	1
mmu-miR-3068-3p	9	13	12	15	28	19	8	19	18	19	13	15
mmu-miR-323-3p	0	0	0	0	0	1	0	0	0	0	0	1
mmu-miR-6896-5p	0	0	0	0	0	1	0	0	0	0	0	1
mmu-miR-7069-3p	0	0	0	0	0	1	0	0	0	0	0	1
mmu-miR-7659-5p	0	0	0	0	0	1	0	0	0	0	0	1
mmu-miR-7684-5p	0	0	0	0	0	1	0	0	0	0	0	1
mmu-miR-25-3p	811	865	745	744	1043	1276	853	1073	816	784	942	1144
mmu-miR-146b-5p	417	468	394	435	1216	972	488	591	624	821	611	577
mmu-miR-7236-3p	0	3	1	1	1	0	1	1	3	0	1	0
mmu-miR-193a-3p	0	1	0	2	0	0	1	0	1	0	1	0
mmu-miR-28a-3p	91	76	118	50	134	119	79	128	90	56	108	152
mmu-miR-34b-3p	0	1	1	0	0	1	0	0	1	1	1	0
mmu-miR-3072-5p	0	0	1	0	0	0	0	0	0	0	1	0
mmu-miR-344b-3p	0	0	1	0	0	0	0	0	0	0	1	0
mmu-miR-34c-3p	0	0	1	0	0	0	0	0	0	0	1	0
mmu-miR-431-3p	0	0	1	0	0	0	0	0	0	0	1	0
mmu-miR-6922-3p	0	0	1	0	0	0	0	0	0	0	1	0
mmu-miR-6980-5p	0	0	1	0	0	0	0	0	0	0	1	0
mmu-miR-7679-5p	0	0	1	0	0	0	0	0	0	0	1	0
mmu-miR-673-3p	1	0	0	0	0	1	2	0	1	0	0	0
mmu-miR-7667-3p	1	1	2	0	3	4	1	2	3	0	5	2
mmu-miR-138-5p	4	1	3	1	0	3	2	1	1	3	2	1
mmu-miR-487b-3p	1	0	0	0	3	0	1	1	0	1	0	0
mmu-miR-6351	1	0	1	0	3	0	1	0	1	0	1	3
mmu-miR-181d-5p	75	100	98	70	123	109	86	125	77	68	95	110
mmu-miR-195b	0	0	1	1	1	0	1	0	1	0	0	1
mmu-miR-6380	0	0	0	1	1	0	0	0	1	0	1	0
mmu-miR-6769b-3p	1	0	3	0	1	3	1	1	1	1	1	2
mmu-miR-130b-3p	14	22	12	15	25	18	18	13	13	11	22	25
mmu-miR-1306-3p	1	1	1	1	3	1	1	1	0	3	1	1
mmu-miR-3082-3p	7	9	8	3	7	6	7	2	8	10	4	7
mmu-miR-6994-5p	0	0	0	0	0	1	0	0	0	1	0	0
mmu-miR-7017-5p	0	0	0	0	0	1	0	0	0	1	0	0
mmu-miR-6970-5p	0	0	1	1	0	1	0	1	1	0	1	0
mmu-miR-6908-3p	0	0	0	0	1	0	0	0	0	1	1	0
mmu-miR-6379	1	0	0	0	1	0	0	0	0	0	2	0
mmu-miR-3109-5p	1	0	0	0	0	1	0	0	0	0	2	0
mmu-miR-379-5p	10	18	9	21	9	12	11	16	17	12	10	15
mmu-miR-350-3p	2	3	6	7	3	4	6	3	4	4	4	3
mmu-miR-7082-3p	0	0	1	0	0	0	1	1	0	0	0	0
mmu-miR-671-5p	6	8	6	8	4	6	8	7	11	4	2	3
mmu-miR-6339	0	1	3	0	0	1	0	1	0	2	1	2
mmu-miR-125b-5p	1605	1354	1151	1340	820	644	1787	886	1234	1129	1041	623
mmu-miR-3092-5p	0	0	0	0	1	1	0	0	0	0	1	1
mmu-miR-187-3p	4	9	7	7	7	4	9	9	1	9	4	4
mmu-miR-1941-3p	0	1	0	0	1	3	0	0	0	0	4	2
mmu-miR-133b-3p	470	428	394	455	526	364	481	448	434	422	440	434
mmu-miR-152-5p	16	25	37	19	33	49	22	42	24	20	38	38
mmu-miR-326-3p	1	1	4	4	1	1	2	2	3	0	2	5
mmu-miR-144-3p	315	272	226	374	232	286	543	228	208	280	234	156
mmu-miR-350-5p	4	4	3	2	0	6	2	3	3	1	7	4
mmu-miR-92a-1-5p	2	3	2	3	1	3	4	2	1	4	1	0
mmu-miR-6900-3p	2	2	1	1	0	0	1	1	0	1	1	1
mmu-miR-433-3p	0	3	1	2	0	3	1	1	4	0	3	1
mmu-miR-133c	0	2	3	5	3	1	4	1	1	3	2	2
mmu-miR-1b-5p	8366	9505	7562	9439	4160	4145	8451	8469	9175	8001	4312	3559
mmu-miR-33-3p	0	1	0	0	0	1	1	1	0	0	0	0
mmu-miR-1249-5p	0	0	0	0	1	0	1	0	0	1	0	0
mmu-miR-3073b-5p	0	1	0	0	1	0	0	0	0	1	1	0
mmu-miR-876-5p	0	1	0	0	1	0	0	0	0	1	1	0
mmu-miR-26a-2-3p	2	3	4	0	4	7	2	2	1	7	6	4
mmu-miR-669c-5p	1	0	0	0	1	3	0	0	0	1	3	2
mmu-miR-7654-3p	0	2	0	1	1	4	0	0	0	0	6	2
mmu-miR-705	0	0	0	1	1	0	0	0	0	0	1	2
mmu-miR-362-5p	2	2	3	1	0	1	0	1	6	2	2	0
mmu-miR-20a-3p	1	1	2	1	0	1	1	2	1	2	1	0
mmu-miR-698-5p	0	0	0	0	0	1	0	1	0	1	0	0
mmu-miR-7090-3p	0	0	0	0	0	1	0	1	0	1	0	0
mmu-miR-125a-3p	6	7	9	3	7	7	4	6	4	9	7	10
mmu-miR-201-3p	1	0	0	0	0	0	0	0	0	0	1	0
mmu-miR-871-5p	1	0	0	0	0	0	0	0	0	0	1	0
mmu-miR-7010-3p	1	0	1	0	1	9	4	2	0	3	4	1
mmu-miR-542-3p	44	40	39	44	98	88	29	59	66	78	44	68
mmu-miR-6989-5p	2	1	0	0	0	1	1	0	0	0	2	2
mmu-miR-337-5p	11	6	4	11	4	3	5	5	6	16	4	5
mmu-let-7a-1-3p	67	55	49	68	65	50	56	54	89	61	46	53
mmu-miR-1947-3p	0	1	0	0	0	0	0	0	0	0	1	0

NGS miR (WT = 1,3,5,7,9,11; KO = 2,4,6,8,10,12)

mmu-miR-598-5p	0	1	0	0	0	0	0	0	0	0	1	0
mmu-miR-7022-5p	0	1	0	0	0	0	0	0	0	0	1	0
mmu-miR-7033-5p	0	1	0	0	0	0	0	0	0	0	1	0
mmu-miR-7232-3p	0	1	0	0	0	0	0	0	0	0	1	0
mmu-miR-7673-3p	0	1	0	0	0	0	0	0	0	0	1	0
mmu-miR-136-3p	2	3	3	1	6	3	2	5	3	3	3	2
mmu-miR-147-3p	2	1	0	2	0	0	0	1	0	3	1	1
mmu-miR-7237-5p	0	0	1	0	0	1	1	1	0	0	1	0
mmu-miR-26a-1-3p	0	0	0	0	1	0	0	1	0	0	1	0
mmu-miR-669o-3p	0	0	0	0	1	0	0	1	0	0	1	0
mmu-miR-1188-3p	1	0	0	0	0	0	0	0	0	0	0	1
mmu-miR-1892	1	0	0	0	0	0	0	0	0	0	0	1
mmu-miR-687	1	0	0	0	0	0	0	0	0	0	0	1
mmu-miR-6915-5p	1	0	0	0	0	0	0	0	0	0	0	1
mmu-miR-871-3p	1	1	3	0	3	1	2	2	1	1	3	0
mmu-miR-185-3p	10	8	15	8	21	15	14	12	11	6	18	14
mmu-miR-6982-3p	5	2	2	0	1	3	1	3	6	1	1	1
mmu-miR-132-5p	7	1	3	1	1	0	4	1	4	1	1	3
mmu-miR-92b-5p	2	0	0	0	0	0	1	0	0	0	0	2
mmu-miR-505-3p	1	0	0	1	1	1	0	2	0	1	0	2
mmu-miR-129b-3p	4	6	3	8	9	12	6	2	6	6	8	13
mmu-miR-6946-3p	0	1	0	0	1	1	0	1	0	0	0	2
mmu-miR-1843a-3p	11	7	3	3	4	10	4	5	7	6	6	11
mmu-miR-1950	0	1	0	1	0	1	0	0	0	0	3	0
mmu-miR-467b-5p	0	0	0	1	0	0	1	1	0	0	0	0
mmu-miR-6990-5p	0	1	1	0	0	1	0	0	0	2	0	1
mmu-miR-124-5p	1	0	0	0	0	0	0	0	0	1	0	0
mmu-miR-320-3p	573	551	558	597	499	482	504	543	601	592	531	477
mmu-miR-15a-3p	4	8	4	7	4	1	7	3	1	3	5	8
mmu-miR-3071-3p	1	0	2	1	0	0	1	0	1	1	1	0
mmu-miR-215-5p	55	48	52	68	96	85	48	96	70	62	62	62
mmu-miR-379-3p	2	2	2	1	1	0	0	1	3	1	1	2
mmu-miR-7021-5p	0	0	0	0	0	1	1	0	0	0	1	0
mmu-miR-7036b-3p	0	0	0	0	0	1	1	0	0	0	1	0
mmu-miR-219c-5p	0	0	1	0	0	0	0	0	0	0	0	1
mmu-miR-293-5p	0	0	1	0	0	0	0	0	0	0	0	1
mmu-miR-466p-5p	0	0	1	0	0	0	0	0	0	0	0	1
mmu-miR-7056-3p	0	0	1	0	0	0	0	0	0	0	0	1
mmu-miR-876-3p	0	0	1	0	0	0	0	0	0	0	0	1
mmu-miR-669l-5p	1	1	0	1	3	0	2	1	0	4	0	0
mmu-miR-6952-3p	1	2	2	0	4	4	2	1	1	3	2	4
mmu-miR-6395	1	1	0	0	3	0	1	1	0	1	0	2
mmu-miR-449c-3p	0	1	0	0	0	0	0	1	0	0	0	0
mmu-miR-669m-5p	0	1	0	0	0	0	0	1	0	0	0	0
mmu-miR-7675-3p	0	1	0	0	0	0	0	1	0	0	0	0
mmu-miR-8098	0	1	0	0	0	0	0	1	0	0	0	0
mmu-miR-382-3p	0	0	0	3	1	0	0	1	3	1	0	0
mmu-miR-704	0	1	0	0	0	1	1	1	0	0	0	1
mmu-miR-652-5p	0	1	0	0	0	0	1	0	0	0	1	0
mmu-miR-7027-3p	1	0	1	1	0	0	1	0	0	1	0	1
mmu-miR-1982-5p	0	2	1	1	1	0	1	0	1	1	0	2
mmu-miR-99a-5p	854	798	629	799	771	440	993	560	809	761	676	464
mmu-miR-3074-2-3p	0	3	2	3	1	0	2	3	0	2	1	1
mmu-miR-466q	0	1	1	0	1	0	1	0	0	1	1	0
mmu-miR-7003-3p	1	0	2	0	1	0	0	1	0	1	1	1
mmu-miR-7115-3p	2	6	7	3	0	6	8	5	1	2	4	3
mmu-miR-598-3p	10	4	5	4	3	7	2	6	8	2	7	8
mmu-miR-19b-2-5p	0	1	0	0	1	0	0	0	0	1	0	1
mmu-miR-1197-3p	0	1	0	1	0	0	0	1	0	0	0	1
mmu-miR-191-5p	4846	5971	4311	5610	4812	4456	5573	4546	4941	5450	4556	5015
mmu-let-7a-2-3p	0	1	0	0	0	1	1	0	1	0	0	0
mmu-miR-105	0	0	0	0	3	0	1	0	0	0	2	0
mmu-miR-665-3p	0	0	0	0	1	0	0	0	1	0	0	0
mmu-miR-6954-5p	0	0	0	0	1	0	0	0	1	0	0	0
mmu-miR-203-5p	1	1	0	0	0	0	0	0	0	0	1	1
mmu-let-7a-5p	10574	10366	10115	10866	6479	6909	9575	9019	9559	11677	8193	7498
mmu-miR-709	12	15	22	15	22	31	15	10	20	27	29	15
mmu-miR-466o-3p	0	0	0	0	0	1	0	0	1	0	0	0
mmu-miR-6396	22	11	13	10	19	16	11	8	15	10	18	28
mmu-miR-377-3p	1	0	0	0	0	0	1	1	0	0	0	0
mmu-miR-744-3p	6	5	2	2	1	3	4	3	8	2	2	0
mmu-miR-210-5p	2	3	5	2	4	1	2	1	6	7	1	2
mmu-let-7i-3p	7	3	3	0	9	6	4	2	3	4	7	9
mmu-miR-7224-3p	2	2	0	5	3	1	0	2	0	8	1	3
mmu-miR-19a-3p	2	7	6	8	3	3	6	5	1	4	8	4
mmu-miR-503-5p	0	1	0	1	0	0	0	1	0	1	0	0
mmu-miR-802-5p	10	3	3	2	13	1	5	2	7	3	8	8
mmu-miR-6988-3p	1	2	1	0	1	1	1	2	0	2	1	1
mmu-miR-151-5p	2017	1813	1554	2027	1197	983	2328	1399	1640	1699	1450	1104
mmu-miR-378c	770	922	1020	1019	1198	1346	724	1259	1024	924	1044	1312
mmu-miR-6959-3p	0	1	0	1	0	0	1	0	0	0	0	1
mmu-miR-466c-3p	0	1	0	0	0	0	1	0	0	0	0	0
mmu-miR-7013-5p	0	1	0	0	0	0	1	0	0	0	0	0
mmu-miR-7028-5p	0	1	0	0	0	0	1	0	0	0	0	0
mmu-miR-7043-5p	0	1	0	0	0	0	1	0	0	0	0	0
mmu-miR-7225-3p	0	1	0	0	0	0	1	0	0	0	0	0
mmu-miR-7229-3p	0	1	0	0	0	0	1	0	0	0	0	0
mmu-miR-92a-3p	7167	7327	7215	3747	8888	10917	7627	9348	5805	3196	8252	11109
mmu-miR-3065-5p	0	0	1	1	0	3	2	1	0	0	1	1
mmu-miR-137-5p	0	0	0	1	0	0	0	0	0	0	0	1
mmu-miR-7081-3p	0	0	0	1	0	0	0	0	0	0	0	1
mmu-miR-1668	0	1	1	0	1	1	1	1	0	1	1	2
mmu-miR-130a-5p	0	0	0	0	0	1	0	0	0	0	1	0
mmu-miR-6398	0	0	0	0	0	1	0	0	0	0	1	0
mmu-miR-127-3p	402	477	456	553	494	479	336	467	399	632	473	553
mmu-miR-204-3p	0	1	1	1	0	0	0	1	1	0	1	0
mmu-miR-7230-5p	0	2	1	1	0	0	0	0	1	1	1	0

mmu-miR-103-1-5p	0	0	0	0	0	0	0	0	0	0	0	0
mmu-miR-103-2-5p	0	0	0	0	0	0	0	0	0	0	0	0
mmu-miR-1188-5p	0	0	0	0	0	0	0	0	0	0	0	0
mmu-miR-1193-3p	0	0	0	0	0	0	0	0	0	0	0	0
mmu-miR-1193-5p	0	0	0	0	0	0	0	0	0	0	0	0
mmu-miR-1197-5p	0	0	0	0	0	0	0	0	0	0	0	0
mmu-miR-124-3p	0	0	0	0	0	0	0	0	0	0	0	0
mmu-miR-1251-5p	0	0	0	0	0	0	0	0	0	0	0	0
mmu-miR-1264-3p	0	0	0	0	0	0	0	0	0	0	0	0
mmu-miR-1264-5p	0	0	0	0	0	0	0	0	0	0	0	0
mmu-miR-133b-5p	0	0	0	0	0	0	0	0	0	0	0	0
mmu-miR-134-3p	0	0	0	0	0	0	0	0	0	0	0	0
mmu-miR-138-1-3p	0	0	0	0	0	0	0	0	0	0	0	0
mmu-miR-153-5p	0	0	0	0	0	0	0	0	0	0	0	0
mmu-miR-154-3p	0	0	0	0	0	0	0	0	0	0	0	0
mmu-miR-181b-1-3p	0	0	0	0	0	0	0	0	0	0	0	0
mmu-miR-183-3p	0	0	0	0	0	0	0	0	0	0	0	0
mmu-miR-1894-5p	0	0	0	0	0	0	0	0	0	0	0	0
mmu-miR-1900	0	0	0	0	0	0	0	0	0	0	0	0
mmu-miR-1905	0	0	0	0	0	0	0	0	0	0	0	0
mmu-miR-1928	0	0	0	0	0	0	0	0	0	0	0	0
mmu-miR-1929-3p	0	0	0	0	0	0	0	0	0	0	0	0
mmu-miR-1961	0	0	0	0	0	0	0	0	0	0	0	0
mmu-miR-1964-5p	0	0	0	0	0	0	0	0	0	0	0	0
mmu-miR-196b-3p	0	0	0	0	0	0	0	0	0	0	0	0
mmu-miR-200b-5p	0	0	0	0	0	0	0	0	0	0	0	0
mmu-miR-20b-3p	0	0	0	0	0	0	0	0	0	0	0	0
mmu-miR-216c-5p	0	0	0	0	0	0	0	0	0	0	0	0
mmu-miR-218-2-3p	0	0	0	0	0	0	0	0	0	0	0	0
mmu-miR-219b-5p	0	0	0	0	0	0	0	0	0	0	0	0
mmu-miR-290a-5p	0	0	0	0	0	0	0	0	0	0	0	0
mmu-miR-290b-3p	0	0	0	0	0	0	0	0	0	0	0	0
mmu-miR-291a-5p	0	0	0	0	0	0	0	0	0	0	0	0
mmu-miR-292-5p	0	0	0	0	0	0	0	0	0	0	0	0
mmu-miR-295-3p	0	0	0	0	0	0	0	0	0	0	0	0
mmu-miR-295-5p	0	0	0	0	0	0	0	0	0	0	0	0
mmu-miR-297a-3p	0	0	0	0	0	0	0	0	0	0	0	0
mmu-miR-297c-3p	0	0	0	0	0	0	0	0	0	0	0	0
mmu-miR-299b-3p	0	0	0	0	0	0	0	0	0	0	0	0
mmu-miR-299b-5p	0	0	0	0	0	0	0	0	0	0	0	0
mmu-miR-302a-5p	0	0	0	0	0	0	0	0	0	0	0	0
mmu-miR-302c-5p	0	0	0	0	0	0	0	0	0	0	0	0
mmu-miR-302d-5p	0	0	0	0	0	0	0	0	0	0	0	0
mmu-miR-3062-5p	0	0	0	0	0	0	0	0	0	0	0	0
mmu-miR-3067-3p	0	0	0	0	0	0	0	0	0	0	0	0
mmu-miR-3070b-5p	0	0	0	0	0	0	0	0	0	0	0	0
mmu-miR-3073a-5p	0	0	0	0	0	0	0	0	0	0	0	0
mmu-miR-3078-3p	0	0	0	0	0	0	0	0	0	0	0	0
mmu-miR-3085-3p	0	0	0	0	0	0	0	0	0	0	0	0
mmu-miR-3091-5p	0	0	0	0	0	0	0	0	0	0	0	0
mmu-miR-3092-3p	0	0	0	0	0	0	0	0	0	0	0	0
mmu-miR-3093-3p	0	0	0	0	0	0	0	0	0	0	0	0
mmu-miR-3093-5p	0	0	0	0	0	0	0	0	0	0	0	0
mmu-miR-3101-5p	0	0	0	0	0	0	0	0	0	0	0	0
mmu-miR-3104-3p	0	0	0	0	0	0	0	0	0	0	0	0
mmu-miR-3106-3p	0	0	0	0	0	0	0	0	0	0	0	0
mmu-miR-3108-5p	0	0	0	0	0	0	0	0	0	0	0	0
mmu-miR-3112-5p	0	0	0	0	0	0	0	0	0	0	0	0
mmu-miR-323-5p	0	0	0	0	0	0	0	0	0	0	0	0
mmu-miR-327	0	0	0	0	0	0	0	0	0	0	0	0
mmu-miR-344c-5p	0	0	0	0	0	0	0	0	0	0	0	0
mmu-miR-344e-5p	0	0	0	0	0	0	0	0	0	0	0	0
mmu-miR-344f-5p	0	0	0	0	0	0	0	0	0	0	0	0
mmu-miR-344g-5p	0	0	0	0	0	0	0	0	0	0	0	0
mmu-miR-344h-5p	0	0	0	0	0	0	0	0	0	0	0	0
mmu-miR-3544-3p	0	0	0	0	0	0	0	0	0	0	0	0
mmu-miR-3547-3p	0	0	0	0	0	0	0	0	0	0	0	0
mmu-miR-365-2-5p	0	0	0	0	0	0	0	0	0	0	0	0
mmu-miR-374b-3p	0	0	0	0	0	0	0	0	0	0	0	0
mmu-miR-410-5p	0	0	0	0	0	0	0	0	0	0	0	0
mmu-miR-412-3p	0	0	0	0	0	0	0	0	0	0	0	0
mmu-miR-412-5p	0	0	0	0	0	0	0	0	0	0	0	0
mmu-miR-429-5p	0	0	0	0	0	0	0	0	0	0	0	0
mmu-miR-448-3p	0	0	0	0	0	0	0	0	0	0	0	0
mmu-miR-449b	0	0	0	0	0	0	0	0	0	0	0	0
mmu-miR-453	0	0	0	0	0	0	0	0	0	0	0	0
mmu-miR-465a-3p	0	0	0	0	0	0	0	0	0	0	0	0
mmu-miR-466b-3p	0	0	0	0	0	0	0	0	0	0	0	0
mmu-miR-466d-5p	0	0	0	0	0	0	0	0	0	0	0	0
mmu-miR-466f	0	0	0	0	0	0	0	0	0	0	0	0
mmu-miR-466f-5p	0	0	0	0	0	0	0	0	0	0	0	0
mmu-miR-466j	0	0	0	0	0	0	0	0	0	0	0	0
mmu-miR-466l-3p	0	0	0	0	0	0	0	0	0	0	0	0
mmu-miR-466l-5p	0	0	0	0	0	0	0	0	0	0	0	0
mmu-miR-466p-3p	0	0	0	0	0	0	0	0	0	0	0	0
mmu-miR-467a-3p	0	0	0	0	0	0	0	0	0	0	0	0
mmu-miR-467c-3p	0	0	0	0	0	0	0	0	0	0	0	0
mmu-miR-467f	0	0	0	0	0	0	0	0	0	0	0	0
mmu-miR-471-3p	0	0	0	0	0	0	0	0	0	0	0	0
mmu-miR-485-3p	0	0	0	0	0	0	0	0	0	0	0	0
mmu-miR-494-5p	0	0	0	0	0	0	0	0	0	0	0	0
mmu-miR-495-5p	0	0	0	0	0	0	0	0	0	0	0	0
mmu-miR-504-3p	0	0	0	0	0	0	0	0	0	0	0	0
mmu-miR-509-5p	0	0	0	0	0	0	0	0	0	0	0	0
mmu-miR-5104	0	0	0	0	0	0	0	0	0	0	0	0
mmu-miR-5131	0	0	0	0	0	0	0	0	0	0	0	0
mmu-miR-544-3p	0	0	0	0	0	0	0	0	0	0	0	0

mmu-miR-544-5p	0	0	0	0	0	0	0	0	0	0	0	0
mmu-miR-551b-3p	0	0	0	0	0	0	0	0	0	0	0	0
mmu-miR-5618-3p	0	0	0	0	0	0	0	0	0	0	0	0
mmu-miR-5618-5p	0	0	0	0	0	0	0	0	0	0	0	0
mmu-miR-5622-5p	0	0	0	0	0	0	0	0	0	0	0	0
mmu-miR-5623-3p	0	0	0	0	0	0	0	0	0	0	0	0
mmu-miR-5624-5p	0	0	0	0	0	0	0	0	0	0	0	0
mmu-miR-5626-5p	0	0	0	0	0	0	0	0	0	0	0	0
mmu-miR-5710	0	0	0	0	0	0	0	0	0	0	0	0
mmu-miR-590-3p	0	0	0	0	0	0	0	0	0	0	0	0
mmu-miR-592-3p	0	0	0	0	0	0	0	0	0	0	0	0
mmu-miR-6335	0	0	0	0	0	0	0	0	0	0	0	0
mmu-miR-6349	0	0	0	0	0	0	0	0	0	0	0	0
mmu-miR-6350	0	0	0	0	0	0	0	0	0	0	0	0
mmu-miR-6354	0	0	0	0	0	0	0	0	0	0	0	0
mmu-miR-6362	0	0	0	0	0	0	0	0	0	0	0	0
mmu-miR-6368	0	0	0	0	0	0	0	0	0	0	0	0
mmu-miR-6370	0	0	0	0	0	0	0	0	0	0	0	0
mmu-miR-6374	0	0	0	0	0	0	0	0	0	0	0	0
mmu-miR-6382	0	0	0	0	0	0	0	0	0	0	0	0
mmu-miR-6383	0	0	0	0	0	0	0	0	0	0	0	0
mmu-miR-6387	0	0	0	0	0	0	0	0	0	0	0	0
mmu-miR-6389	0	0	0	0	0	0	0	0	0	0	0	0
mmu-miR-6393	0	0	0	0	0	0	0	0	0	0	0	0
mmu-miR-6397	0	0	0	0	0	0	0	0	0	0	0	0
mmu-miR-6400	0	0	0	0	0	0	0	0	0	0	0	0
mmu-miR-6401	0	0	0	0	0	0	0	0	0	0	0	0
mmu-miR-6403	0	0	0	0	0	0	0	0	0	0	0	0
mmu-miR-6407	0	0	0	0	0	0	0	0	0	0	0	0
mmu-miR-6417	0	0	0	0	0	0	0	0	0	0	0	0
mmu-miR-6420	0	0	0	0	0	0	0	0	0	0	0	0
mmu-miR-6540-3p	0	0	0	0	0	0	0	0	0	0	0	0
mmu-miR-6546-3p	0	0	0	0	0	0	0	0	0	0	0	0
mmu-miR-666-3p	0	0	0	0	0	0	0	0	0	0	0	0
mmu-miR-669b-3p	0	0	0	0	0	0	0	0	0	0	0	0
mmu-miR-669b-5p	0	0	0	0	0	0	0	0	0	0	0	0
mmu-miR-669d-2-3p	0	0	0	0	0	0	0	0	0	0	0	0
mmu-miR-669d-3p	0	0	0	0	0	0	0	0	0	0	0	0
mmu-miR-669h-3p	0	0	0	0	0	0	0	0	0	0	0	0
mmu-miR-669h-5p	0	0	0	0	0	0	0	0	0	0	0	0
mmu-miR-669j	0	0	0	0	0	0	0	0	0	0	0	0
mmu-miR-669k-5p	0	0	0	0	0	0	0	0	0	0	0	0
mmu-miR-6715-5p	0	0	0	0	0	0	0	0	0	0	0	0
mmu-miR-677-3p	0	0	0	0	0	0	0	0	0	0	0	0
mmu-miR-680	0	0	0	0	0	0	0	0	0	0	0	0
mmu-miR-683	0	0	0	0	0	0	0	0	0	0	0	0
mmu-miR-686	0	0	0	0	0	0	0	0	0	0	0	0
mmu-miR-688	0	0	0	0	0	0	0	0	0	0	0	0
mmu-miR-6897-5p	0	0	0	0	0	0	0	0	0	0	0	0
mmu-miR-6899-5p	0	0	0	0	0	0	0	0	0	0	0	0
mmu-miR-6903-5p	0	0	0	0	0	0	0	0	0	0	0	0
mmu-miR-6904-5p	0	0	0	0	0	0	0	0	0	0	0	0
mmu-miR-6908-5p	0	0	0	0	0	0	0	0	0	0	0	0
mmu-miR-6913-5p	0	0	0	0	0	0	0	0	0	0	0	0
mmu-miR-6918-5p	0	0	0	0	0	0	0	0	0	0	0	0
mmu-miR-692	0	0	0	0	0	0	0	0	0	0	0	0
mmu-miR-6920-5p	0	0	0	0	0	0	0	0	0	0	0	0
mmu-miR-6928-5p	0	0	0	0	0	0	0	0	0	0	0	0
mmu-miR-6930-5p	0	0	0	0	0	0	0	0	0	0	0	0
mmu-miR-6933-5p	0	0	0	0	0	0	0	0	0	0	0	0
mmu-miR-693-5p	0	0	0	0	0	0	0	0	0	0	0	0
mmu-miR-6936-3p	0	0	0	0	0	0	0	0	0	0	0	0
mmu-miR-6936-5p	0	0	0	0	0	0	0	0	0	0	0	0
mmu-miR-6938-5p	0	0	0	0	0	0	0	0	0	0	0	0
mmu-miR-6940-3p	0	0	0	0	0	0	0	0	0	0	0	0
mmu-miR-6943-5p	0	0	0	0	0	0	0	0	0	0	0	0
mmu-miR-6947-5p	0	0	0	0	0	0	0	0	0	0	0	0
mmu-miR-695	0	0	0	0	0	0	0	0	0	0	0	0
mmu-miR-6958-5p	0	0	0	0	0	0	0	0	0	0	0	0
mmu-miR-6959-5p	0	0	0	0	0	0	0	0	0	0	0	0
mmu-miR-6961-5p	0	0	0	0	0	0	0	0	0	0	0	0
mmu-miR-6963-5p	0	0	0	0	0	0	0	0	0	0	0	0
mmu-miR-6969-5p	0	0	0	0	0	0	0	0	0	0	0	0
mmu-miR-6973a-3p	0	0	0	0	0	0	0	0	0	0	0	0
mmu-miR-6976-5p	0	0	0	0	0	0	0	0	0	0	0	0
mmu-miR-6987-3p	0	0	0	0	0	0	0	0	0	0	0	0
mmu-miR-6987-5p	0	0	0	0	0	0	0	0	0	0	0	0
mmu-miR-6990-3p	0	0	0	0	0	0	0	0	0	0	0	0
mmu-miR-7000-5p	0	0	0	0	0	0	0	0	0	0	0	0
mmu-miR-7001-3p	0	0	0	0	0	0	0	0	0	0	0	0
mmu-miR-7001-5p	0	0	0	0	0	0	0	0	0	0	0	0
mmu-miR-7002-5p	0	0	0	0	0	0	0	0	0	0	0	0
mmu-miR-7011-3p	0	0	0	0	0	0	0	0	0	0	0	0
mmu-miR-7012-5p	0	0	0	0	0	0	0	0	0	0	0	0
mmu-miR-701-3p	0	0	0	0	0	0	0	0	0	0	0	0
mmu-miR-7018-3p	0	0	0	0	0	0	0	0	0	0	0	0
mmu-miR-7023-3p	0	0	0	0	0	0	0	0	0	0	0	0
mmu-miR-7025-3p	0	0	0	0	0	0	0	0	0	0	0	0
mmu-miR-7025-5p	0	0	0	0	0	0	0	0	0	0	0	0
mmu-miR-7028-3p	0	0	0	0	0	0	0	0	0	0	0	0
mmu-miR-7029-3p	0	0	0	0	0	0	0	0	0	0	0	0
mmu-miR-703	0	0	0	0	0	0	0	0	0	0	0	0
mmu-miR-7031-3p	0	0	0	0	0	0	0	0	0	0	0	0
mmu-miR-7031-5p	0	0	0	0	0	0	0	0	0	0	0	0
mmu-miR-7032-3p	0	0	0	0	0	0	0	0	0	0	0	0
mmu-miR-7036-5p	0	0	0	0	0	0	0	0	0	0	0	0
mmu-miR-7036b-5p	0	0	0	0	0	0	0	0	0	0	0	0

mmu-miR-7037-3p	0	0	0	0	0	0	0	0	0	0	0	0
mmu-miR-7049-3p	0	0	0	0	0	0	0	0	0	0	0	0
mmu-miR-7050-5p	0	0	0	0	0	0	0	0	0	0	0	0
mmu-miR-7051-3p	0	0	0	0	0	0	0	0	0	0	0	0
mmu-miR-7055-5p	0	0	0	0	0	0	0	0	0	0	0	0
mmu-miR-7057-5p	0	0	0	0	0	0	0	0	0	0	0	0
mmu-miR-7063-3p	0	0	0	0	0	0	0	0	0	0	0	0
mmu-miR-7064-5p	0	0	0	0	0	0	0	0	0	0	0	0
mmu-miR-7066-3p	0	0	0	0	0	0	0	0	0	0	0	0
mmu-miR-7066-5p	0	0	0	0	0	0	0	0	0	0	0	0
mmu-miR-7071-5p	0	0	0	0	0	0	0	0	0	0	0	0
mmu-miR-7072-3p	0	0	0	0	0	0	0	0	0	0	0	0
mmu-miR-7072-5p	0	0	0	0	0	0	0	0	0	0	0	0
mmu-miR-7076-3p	0	0	0	0	0	0	0	0	0	0	0	0
mmu-miR-7078-5p	0	0	0	0	0	0	0	0	0	0	0	0
mmu-miR-7082-5p	0	0	0	0	0	0	0	0	0	0	0	0
mmu-miR-7084-5p	0	0	0	0	0	0	0	0	0	0	0	0
mmu-miR-7086-3p	0	0	0	0	0	0	0	0	0	0	0	0
mmu-miR-7094-1-5p	0	0	0	0	0	0	0	0	0	0	0	0
mmu-miR-711	0	0	0	0	0	0	0	0	0	0	0	0
mmu-miR-7119-3p	0	0	0	0	0	0	0	0	0	0	0	0
mmu-miR-717	0	0	0	0	0	0	0	0	0	0	0	0
mmu-miR-7220-3p	0	0	0	0	0	0	0	0	0	0	0	0
mmu-miR-7223-3p	0	0	0	0	0	0	0	0	0	0	0	0
mmu-miR-7223-5p	0	0	0	0	0	0	0	0	0	0	0	0
mmu-miR-7238-3p	0	0	0	0	0	0	0	0	0	0	0	0
mmu-miR-7238-5p	0	0	0	0	0	0	0	0	0	0	0	0
mmu-miR-7239-3p	0	0	0	0	0	0	0	0	0	0	0	0
mmu-miR-7243-5p	0	0	0	0	0	0	0	0	0	0	0	0
mmu-miR-742-3p	0	0	0	0	0	0	0	0	0	0	0	0
mmu-miR-742-5p	0	0	0	0	0	0	0	0	0	0	0	0
mmu-miR-743a-3p	0	0	0	0	0	0	0	0	0	0	0	0
mmu-miR-743a-5p	0	0	0	0	0	0	0	0	0	0	0	0
mmu-miR-758-3p	0	0	0	0	0	0	0	0	0	0	0	0
mmu-miR-758-5p	0	0	0	0	0	0	0	0	0	0	0	0
mmu-miR-761	0	0	0	0	0	0	0	0	0	0	0	0
mmu-miR-764-3p	0	0	0	0	0	0	0	0	0	0	0	0
mmu-miR-7646-3p	0	0	0	0	0	0	0	0	0	0	0	0
mmu-miR-7656-3p	0	0	0	0	0	0	0	0	0	0	0	0
mmu-miR-7657-3p	0	0	0	0	0	0	0	0	0	0	0	0
mmu-miR-7657-5p	0	0	0	0	0	0	0	0	0	0	0	0
mmu-miR-7658-5p	0	0	0	0	0	0	0	0	0	0	0	0
mmu-miR-7663-5p	0	0	0	0	0	0	0	0	0	0	0	0
mmu-miR-7671-3p	0	0	0	0	0	0	0	0	0	0	0	0
mmu-miR-7672-5p	0	0	0	0	0	0	0	0	0	0	0	0
mmu-miR-7677-3p	0	0	0	0	0	0	0	0	0	0	0	0
mmu-miR-7683-3p	0	0	0	0	0	0	0	0	0	0	0	0
mmu-miR-7683-5p	0	0	0	0	0	0	0	0	0	0	0	0
mmu-miR-7684-3p	0	0	0	0	0	0	0	0	0	0	0	0
mmu-miR-7686-3p	0	0	0	0	0	0	0	0	0	0	0	0
mmu-miR-7687-3p	0	0	0	0	0	0	0	0	0	0	0	0
mmu-miR-7688-3p	0	0	0	0	0	0	0	0	0	0	0	0
mmu-miR-7689-5p	0	0	0	0	0	0	0	0	0	0	0	0
mmu-miR-8091	0	0	0	0	0	0	0	0	0	0	0	0
mmu-miR-8102	0	0	0	0	0	0	0	0	0	0	0	0
mmu-miR-8106	0	0	0	0	0	0	0	0	0	0	0	0
mmu-miR-8107	0	0	0	0	0	0	0	0	0	0	0	0
mmu-miR-8108	0	0	0	0	0	0	0	0	0	0	0	0
mmu-miR-8109	0	0	0	0	0	0	0	0	0	0	0	0
mmu-miR-8116	0	0	0	0	0	0	0	0	0	0	0	0
mmu-miR-8117	0	0	0	0	0	0	0	0	0	0	0	0
mmu-miR-878-5p	0	0	0	0	0	0	0	0	0	0	0	0
mmu-miR-879-3p	0	0	0	0	0	0	0	0	0	0	0	0
mmu-miR-883a-3p	0	0	0	0	0	0	0	0	0	0	0	0
mmu-miR-883b-3p	0	0	0	0	0	0	0	0	0	0	0	0
mmu-miR-883b-5p	0	0	0	0	0	0	0	0	0	0	0	0
